# Supplementary material for: Synthesis and Antifungal Activity of New butenolide Containing Methoxyacrylate Scaffold
Source: Molecules. 2022 Oct 3;27(19):6541. doi: 10.3390/molecules27196541 (PMC9573425; doi:10.3390/molecules27196541)
Supplement: Supplementary file 1 [file molecules-27-06541-s001.zip › molecules-1940002-supplementary.pdf]

Supporting Information

Synthesis and antifungal activity of novel butenolide containing methoxyacrylate scaffold

Zhang, Qian Li, Yihao Zhao, Bin Xu, Leichuan Ma, Haoyun Wang, Mingan*

Innovation Center of Pesticide Research, College of Science, China Agricultural University, Beijing 100193, China

* Corresponding author. M. A. Wang, Tel.(Fax):86-10-62734093; E-mail: wangma@cau.edu.cn

Contents

1. The ^{1}H and ^{13}C NMR spectra of intermediates and target compounds **III-7~III-10, III-12, III-14, IV-7~IV-10, IV-12, IV-14** and **V-1~VI-14**……S2
2. The high reslution mass spectrometry report spectra of compounds **VI-3**, **VI-8** and **VI-13**……S41
3. The X-ray crystal structure of target compound **IV-6**……S42
4. The cell variance of *S. sclerotiorum* treated with **VI-3** and TRI observed by TEM……S47
5. The mycelium growth inhibition of *S. sclerotiorum* treated with **VI-3** and TRI by SEM……S48
6. Interaction of compounds **V-3**, **V-6**, **VI-7** and TRI with target by molecular docking……S49

1. The ^{1}H and ^{13}C NMR spectra of intermediates and target compounds **III-7~III-10, III-12, III-14, IV-7~IV-10, IV-12, IV-14 and V-1~VI-14**

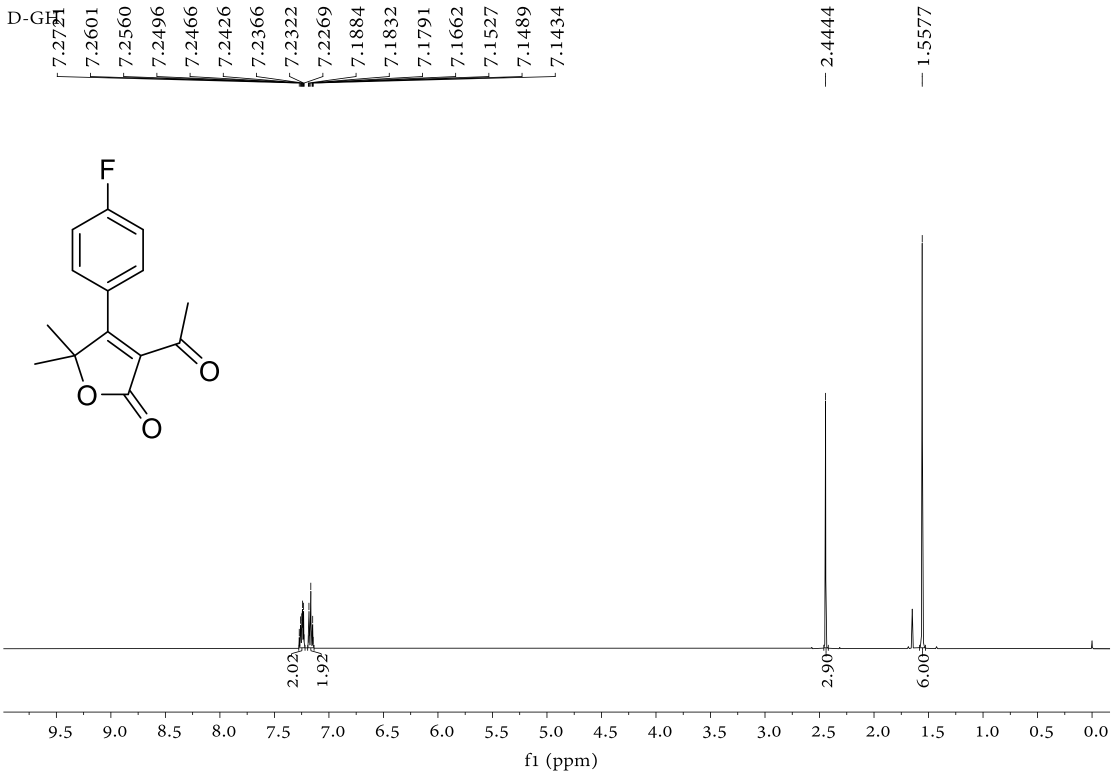

Figure S1 ^{1}H NMR of compound **III-7** (500 MHz, $CDCl_3$)

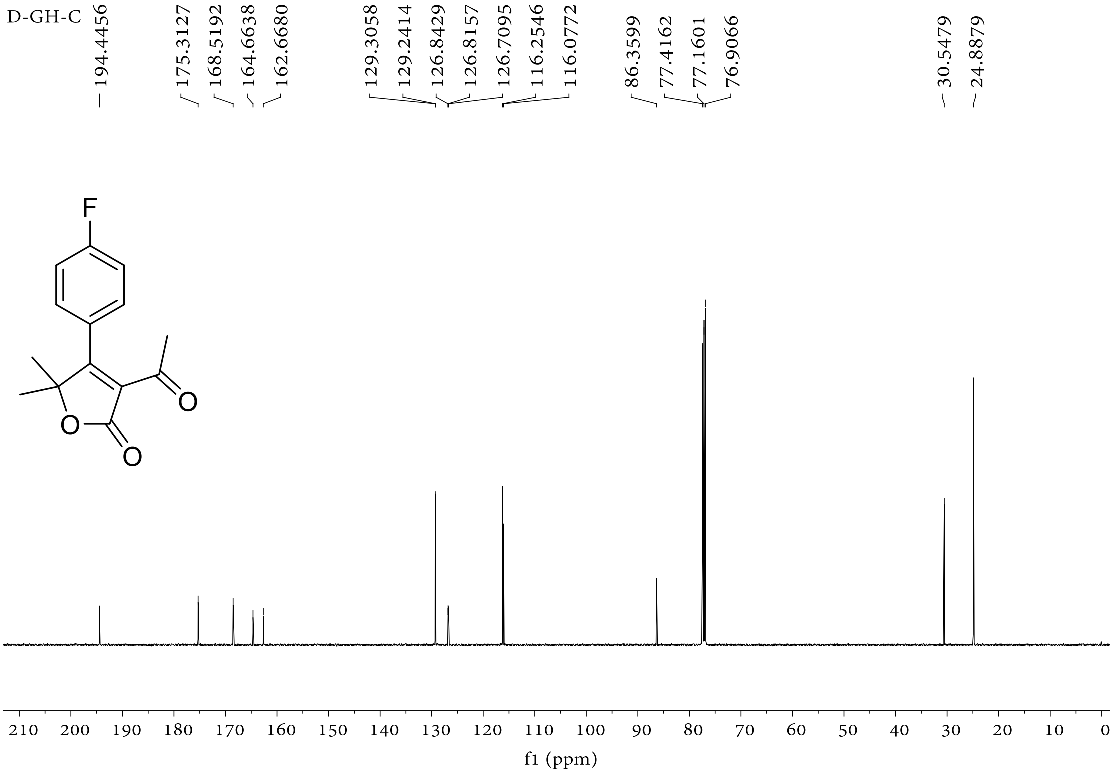

Figure S2 ^{13}C NMR of compound **III-7** (126 MHz, $CDCl_3$)

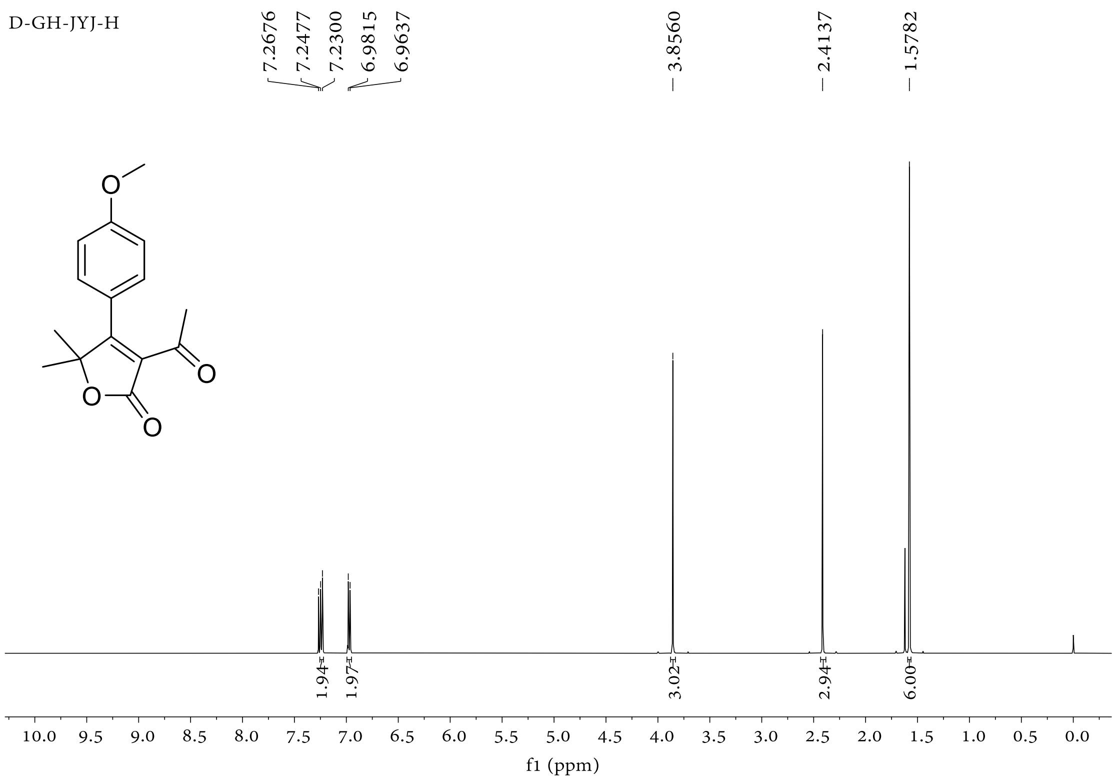

Figure S3 ^{1}H NMR of compound **III-8** (500 MHz, $CDCl_3$)

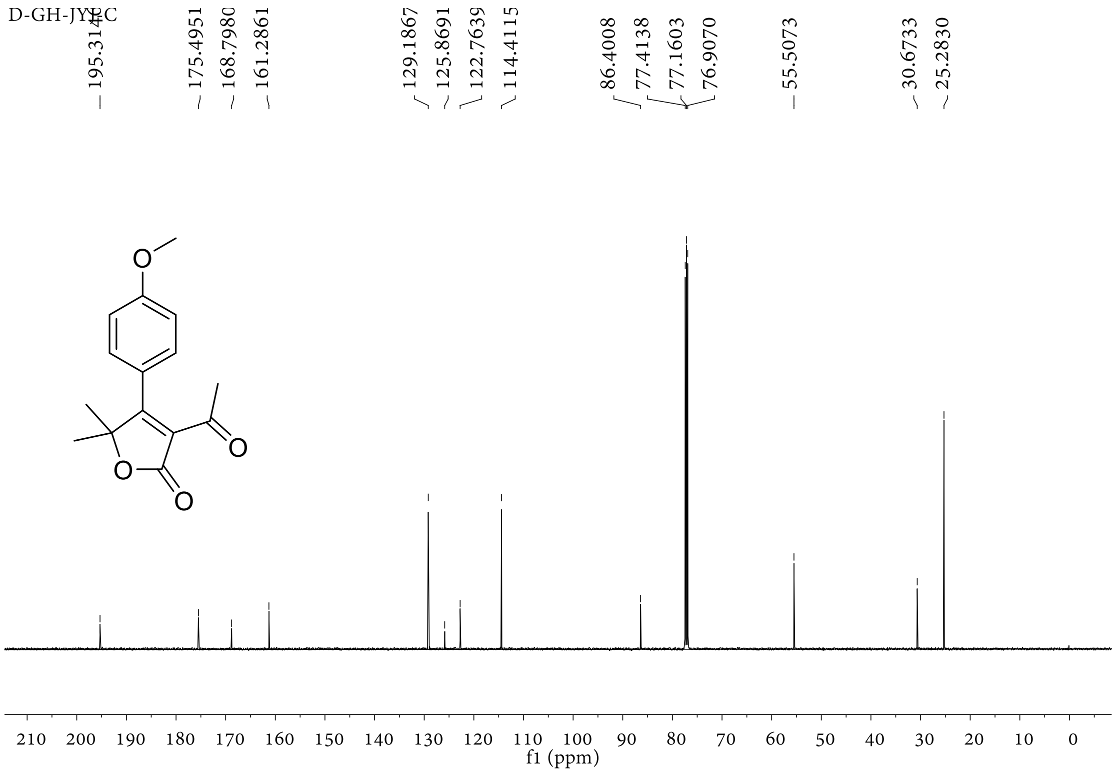

Figure S4 ^{13}C NMR of compound **III-8** (126 MHz, $CDCl_3$)

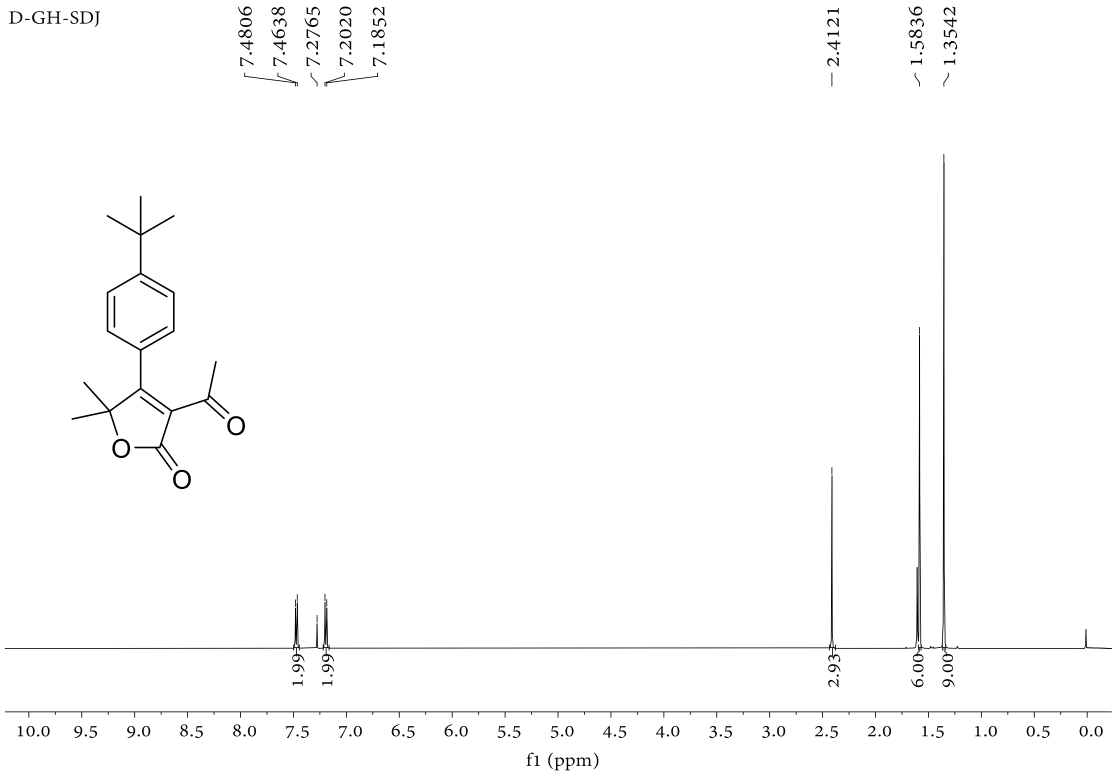

Figure S5 ^1H NMR of compound **III-9** (500 MHz, $CDCl_3$)

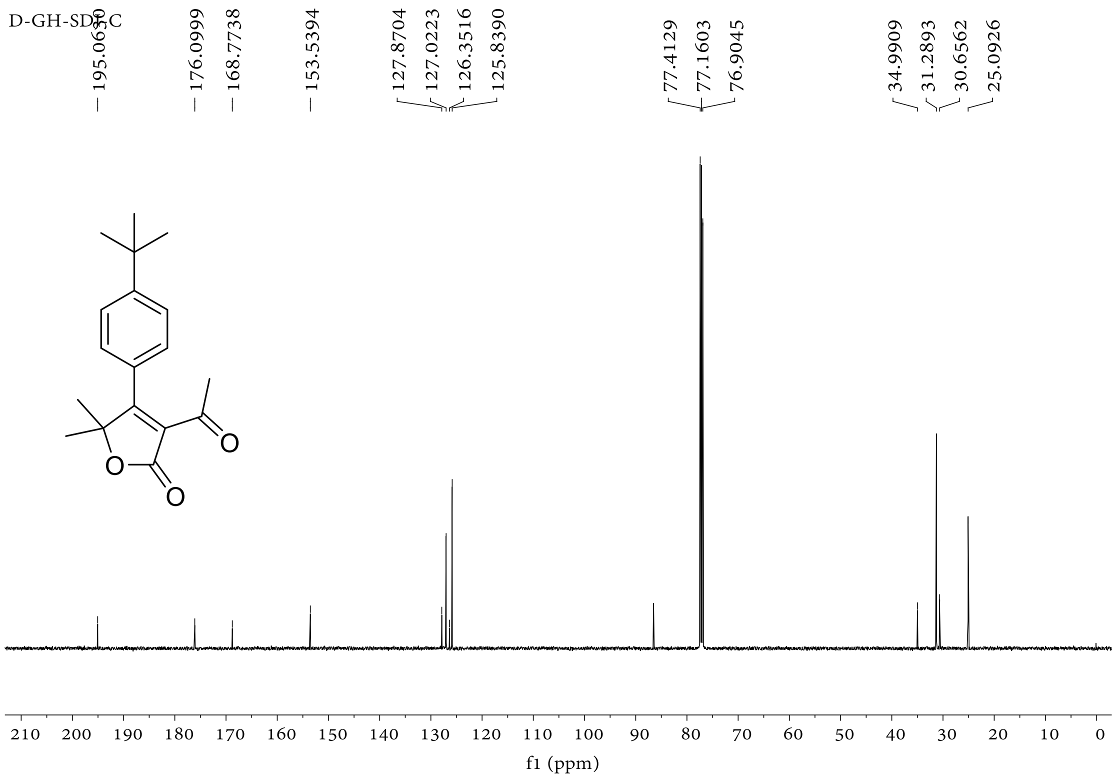

Figure S6 ^{13}C NMR of compound **III-9** (126 MHz, $CDCl_3$)

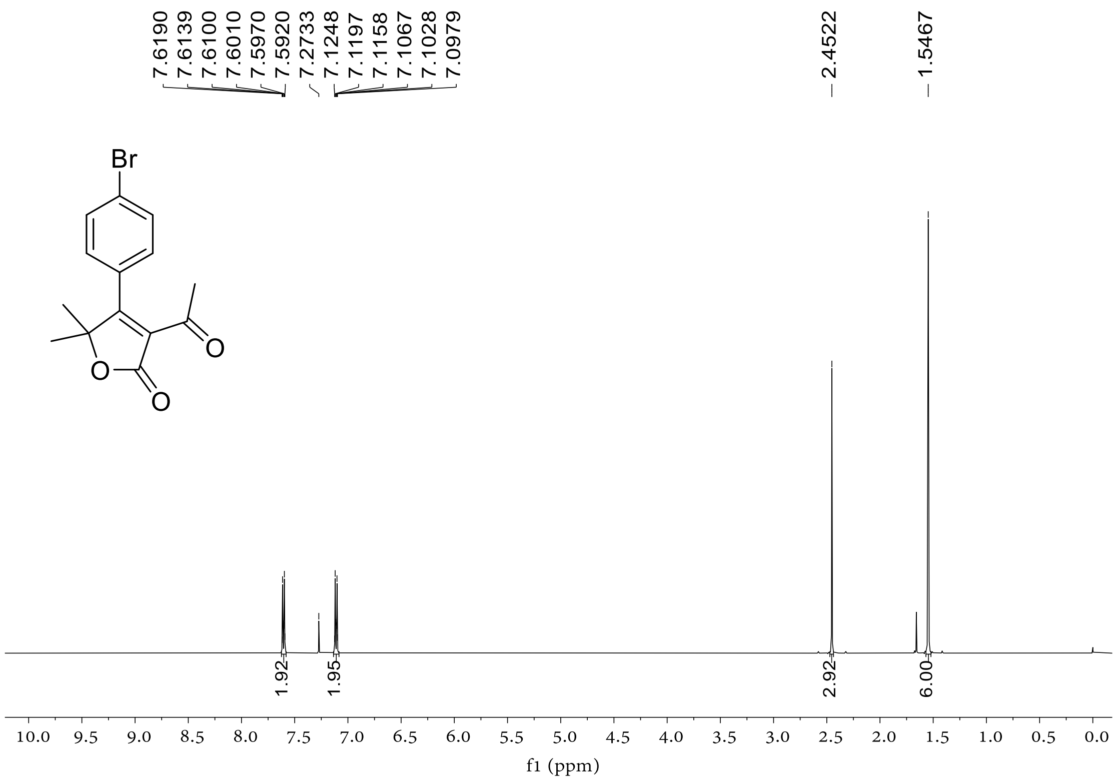

Figure S7 ^{1}H NMR of compound **III-10** (500 MHz, $CDCl_3$)

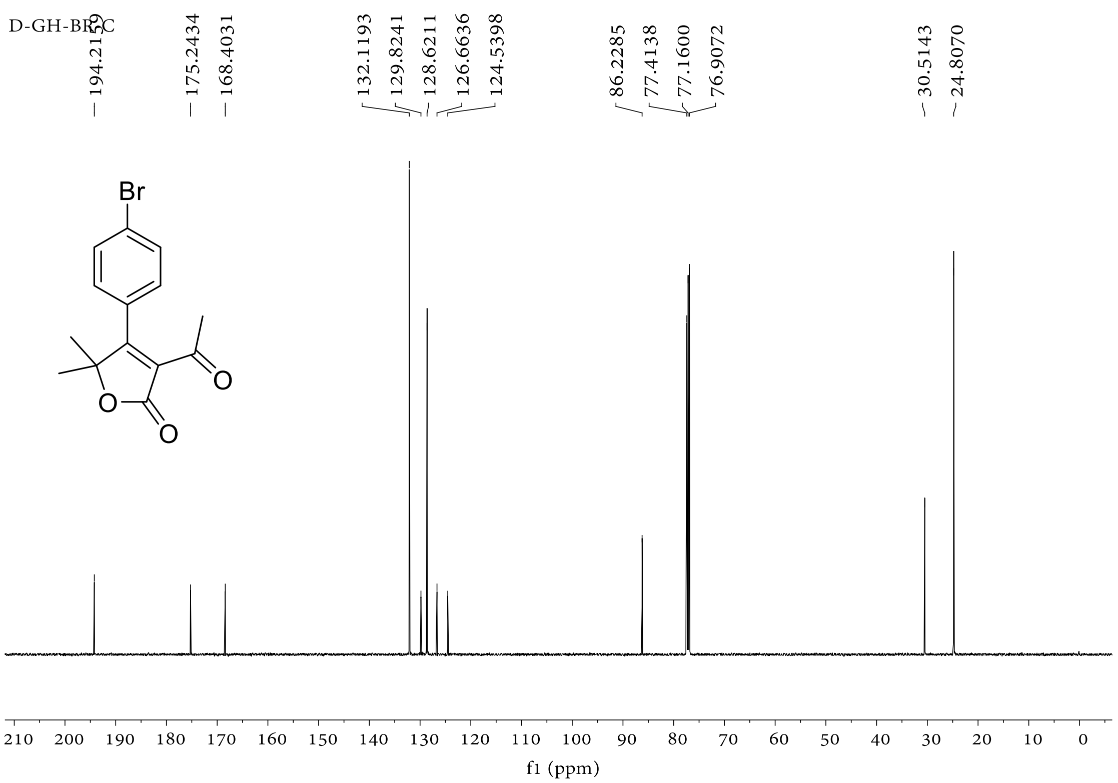

Figure S8 ^{13}C NMR of compound **III-10** (126 MHz, $CDCl_3$)

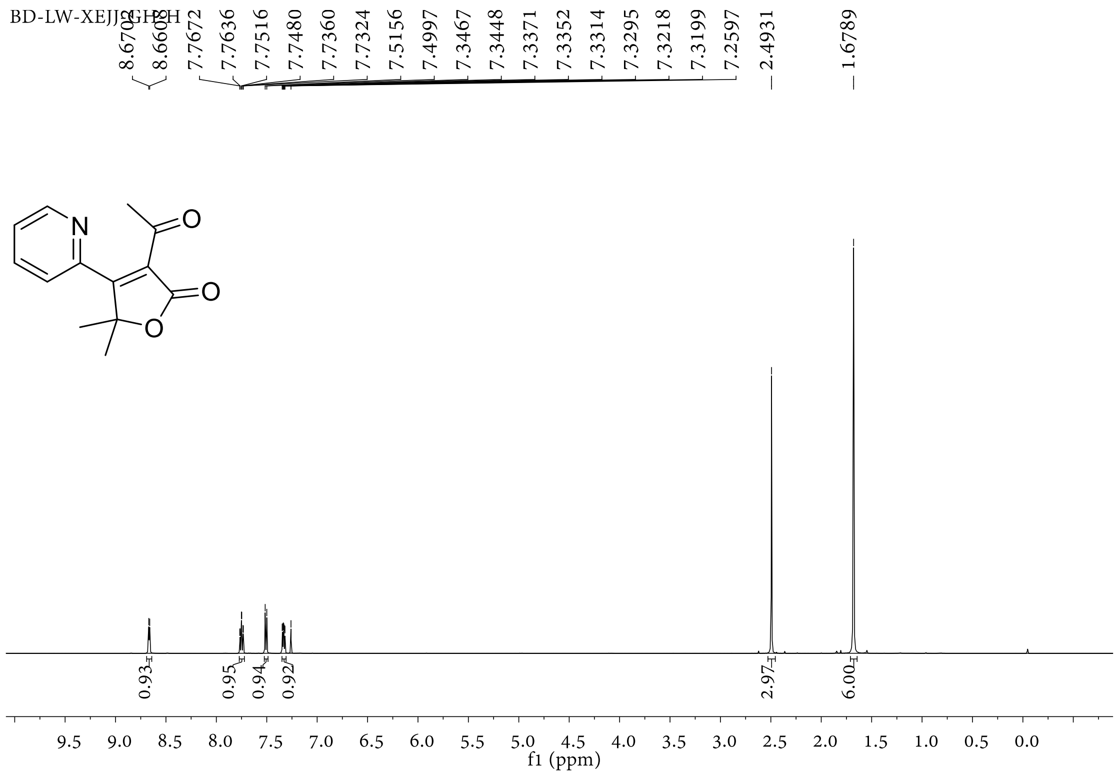

Figure S9 ^{1}H NMR of compound **III-12** (500 MHz, $CDCl_3$)

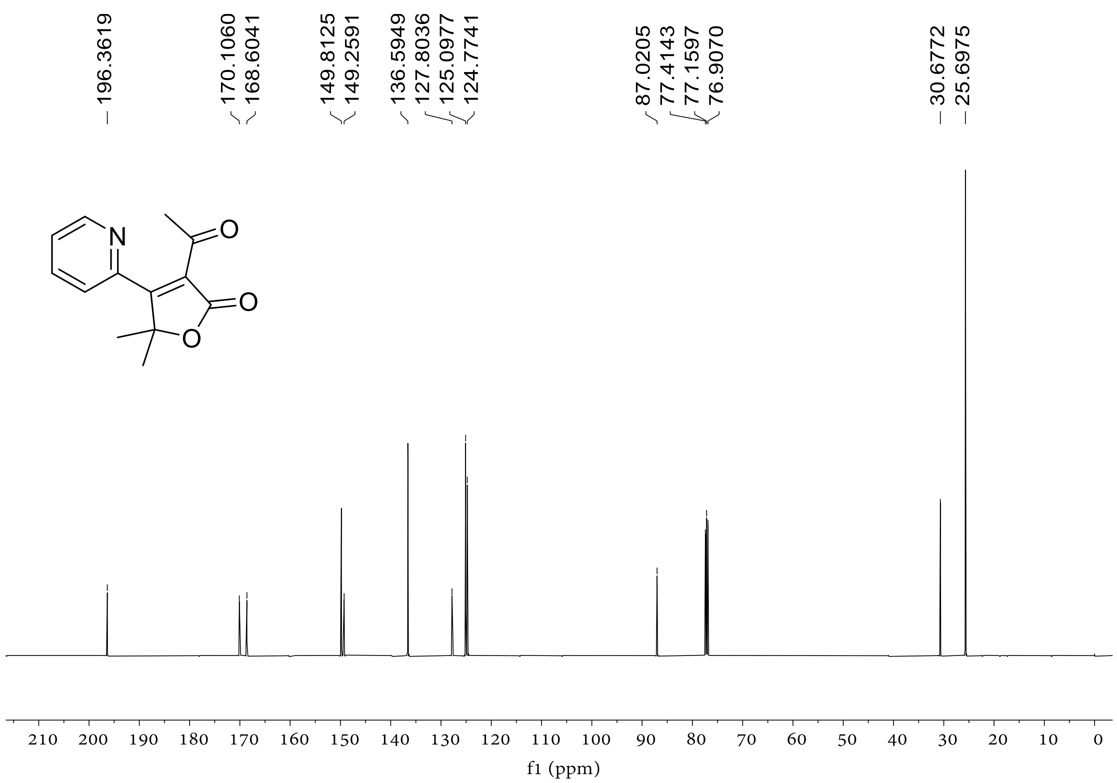

Figure S10 ^{13}C NMR of compound **III-12** (126 MHz, $CDCl_3$)

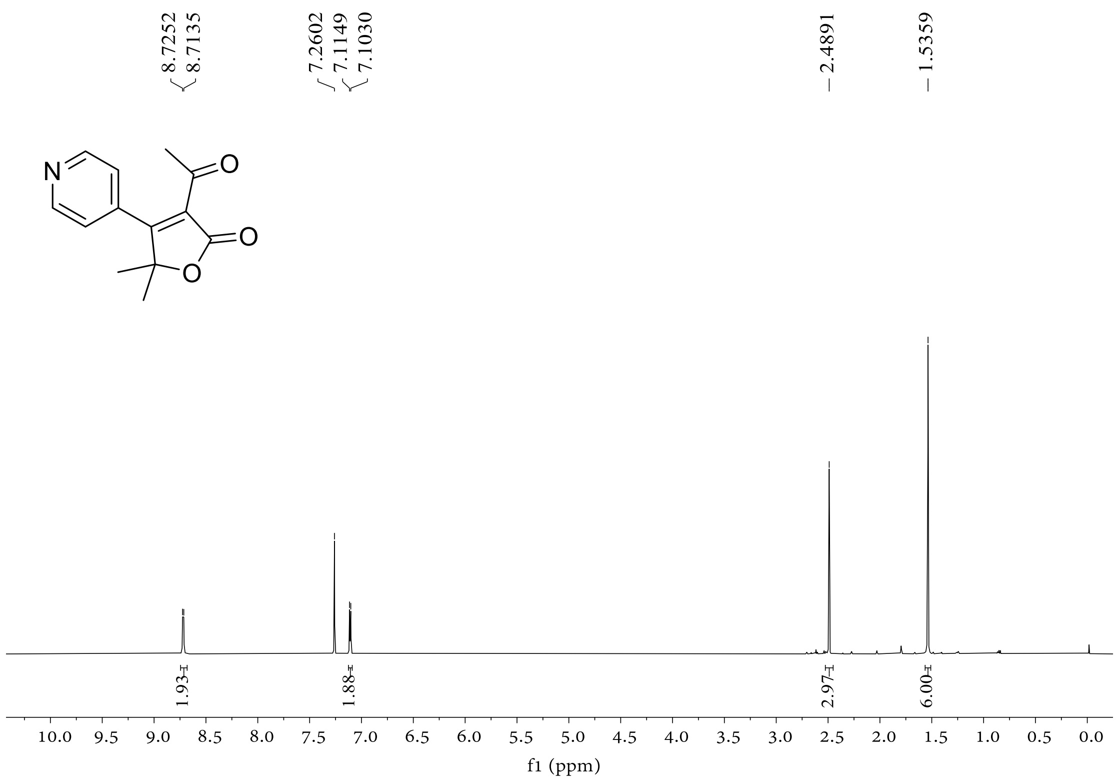

Figure S11 ^{1}H NMR of compound **III-14** (500 MHz, $CDCl_3$)

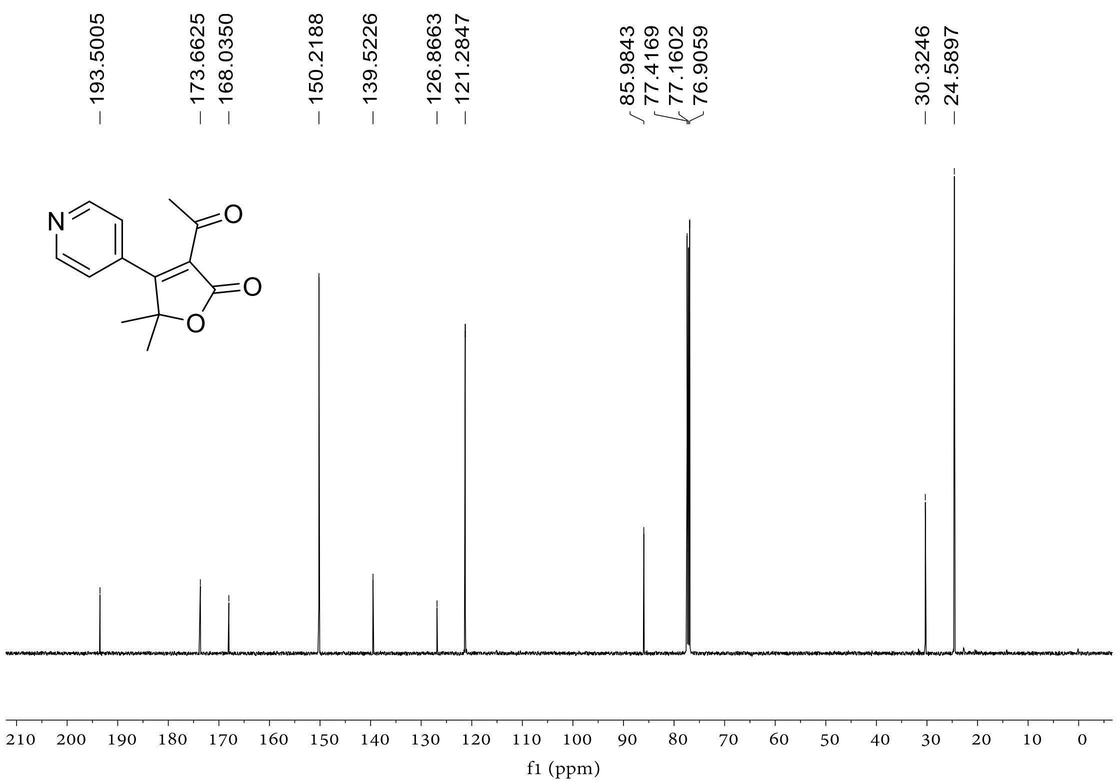

Figure S12 ^{13}C NMR of compound **III-14** (126 MHz, $CDCl_3$)

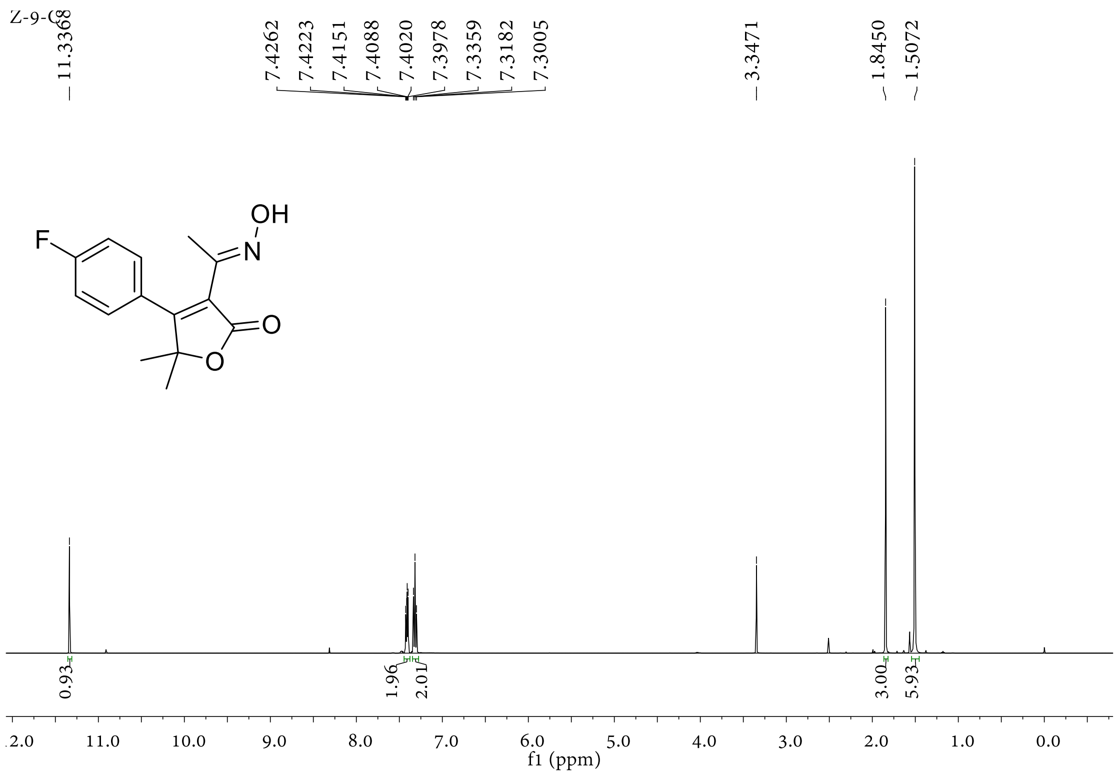

Figure S13 ^{1}H NMR of compound **IV-7** (500 MHz, $CDCl_3$)

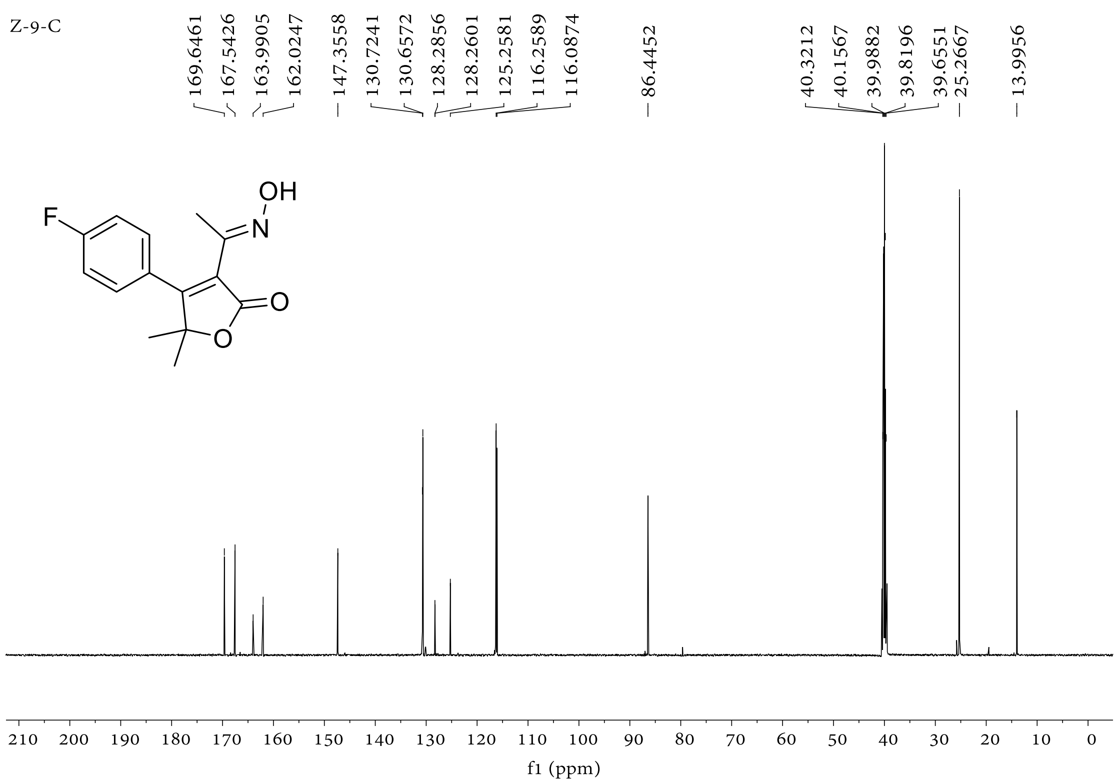

Figure S14 ^{13}C NMR of compound **IV-7** (126 MHz, $CDCl_3$)

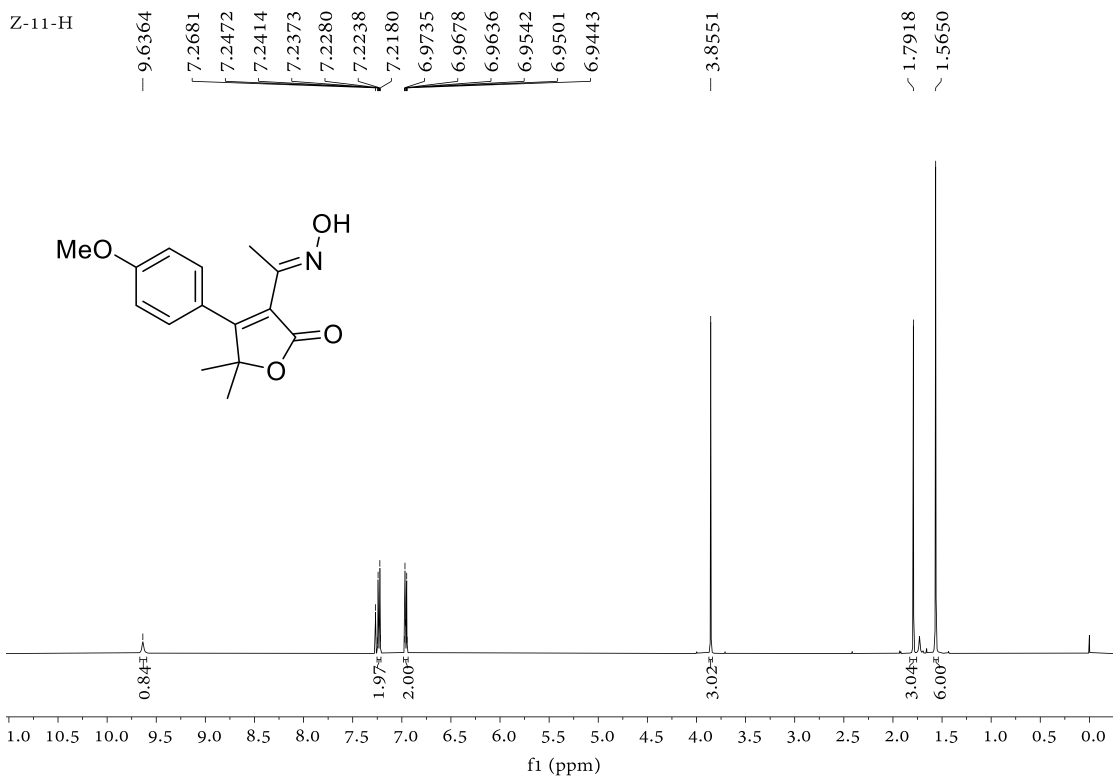

Figure S15 ^{1}H NMR of compound **IV-8** (500 MHz, $CDCl_3$)

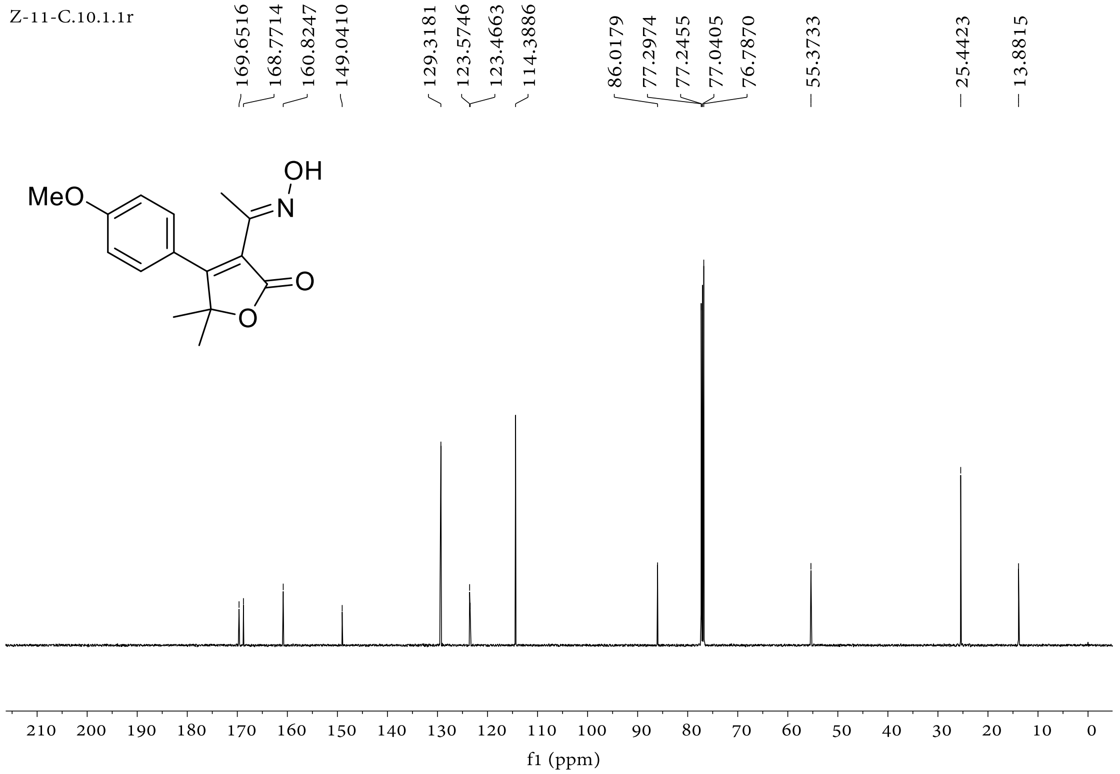

Figure S16 ^{13}C NMR of company **IV-8** (126 MHz, $CDCl_3$)

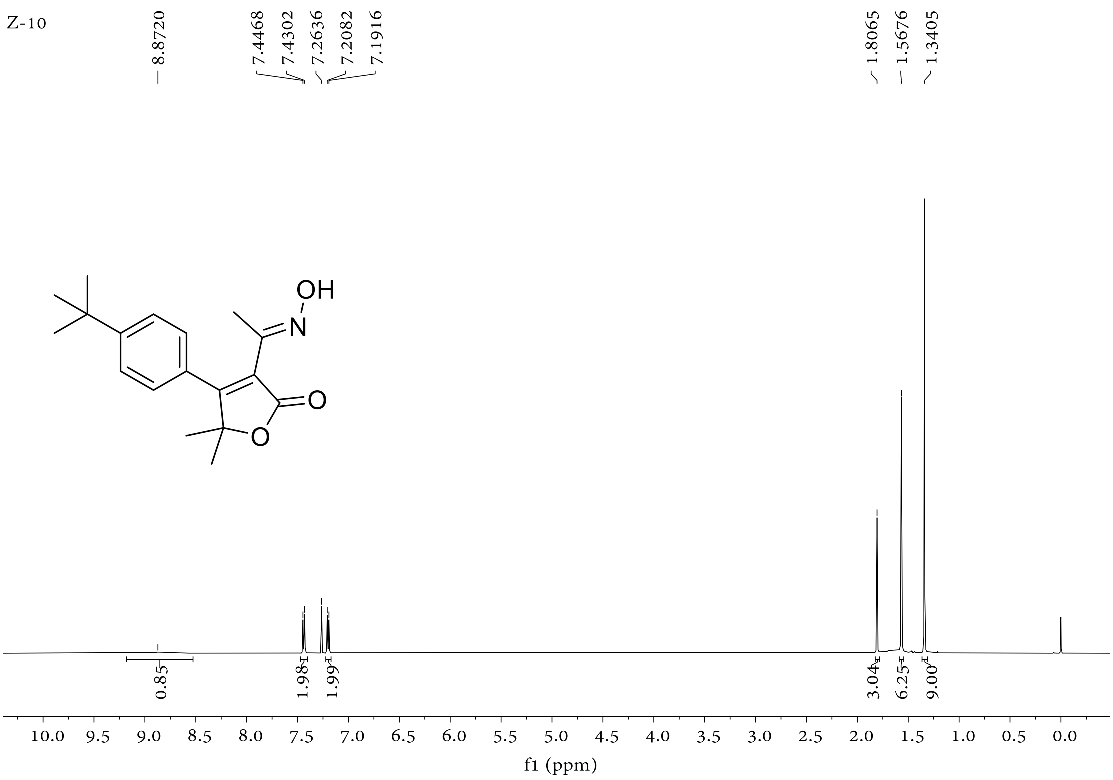

Figure S17 ^{1}H NMR of compound **IV-9** (500 MHz, $CDCl_3$)

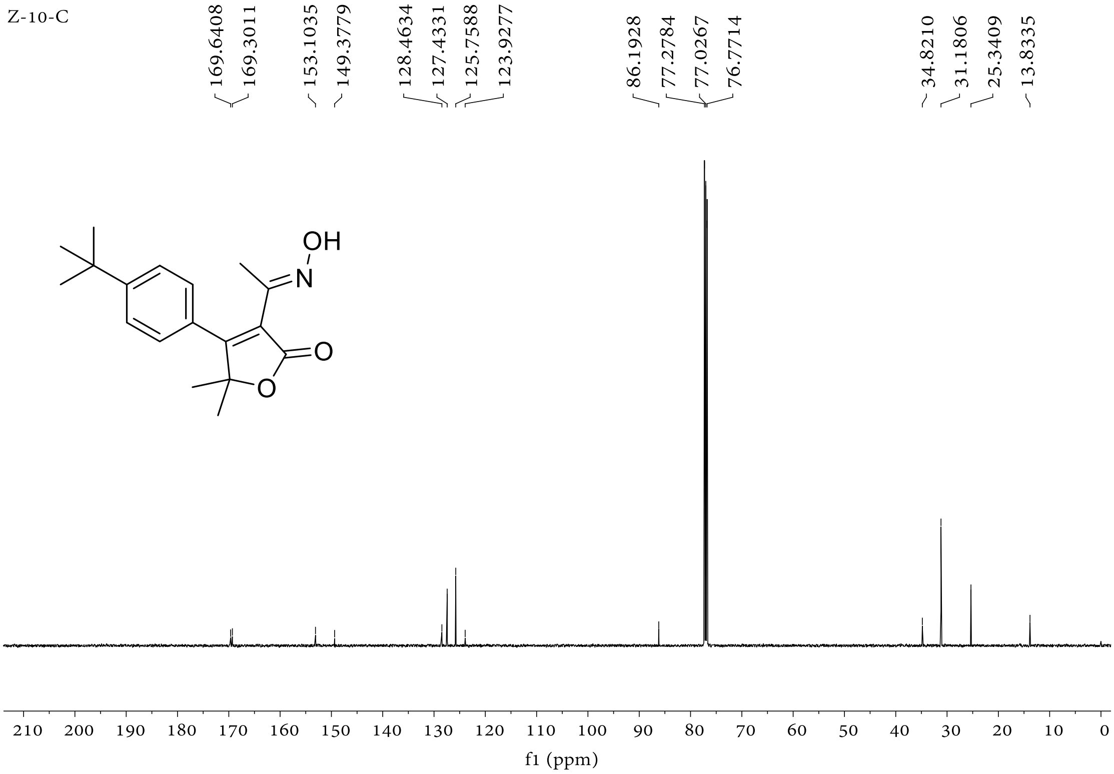

Figure S18 ^{13}C NMR of compound **IV-9** (126 MHz, $CDCl_3$)

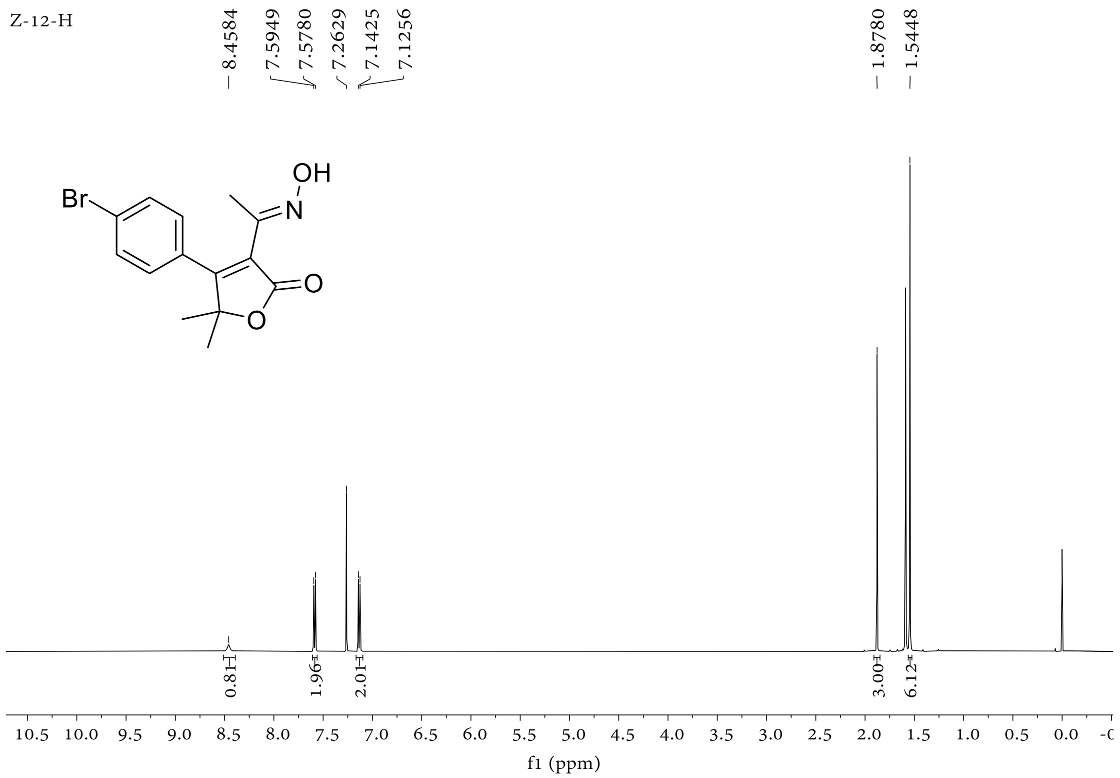

Figure S19 ^{1}H NMR of compound **IV-10** (500 MHz, $CDCl_3$)

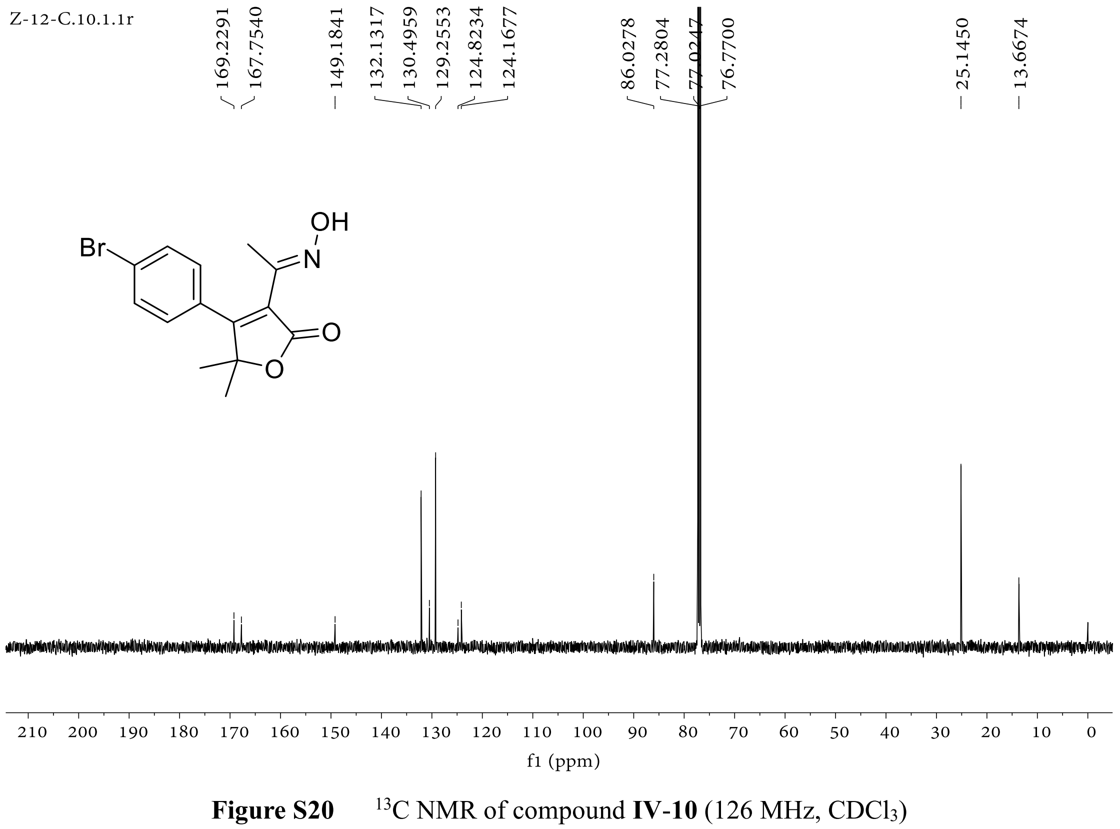

Figure S20 ^{13}C NMR of compound **IV-10** (126 MHz, $CDCl_3$)

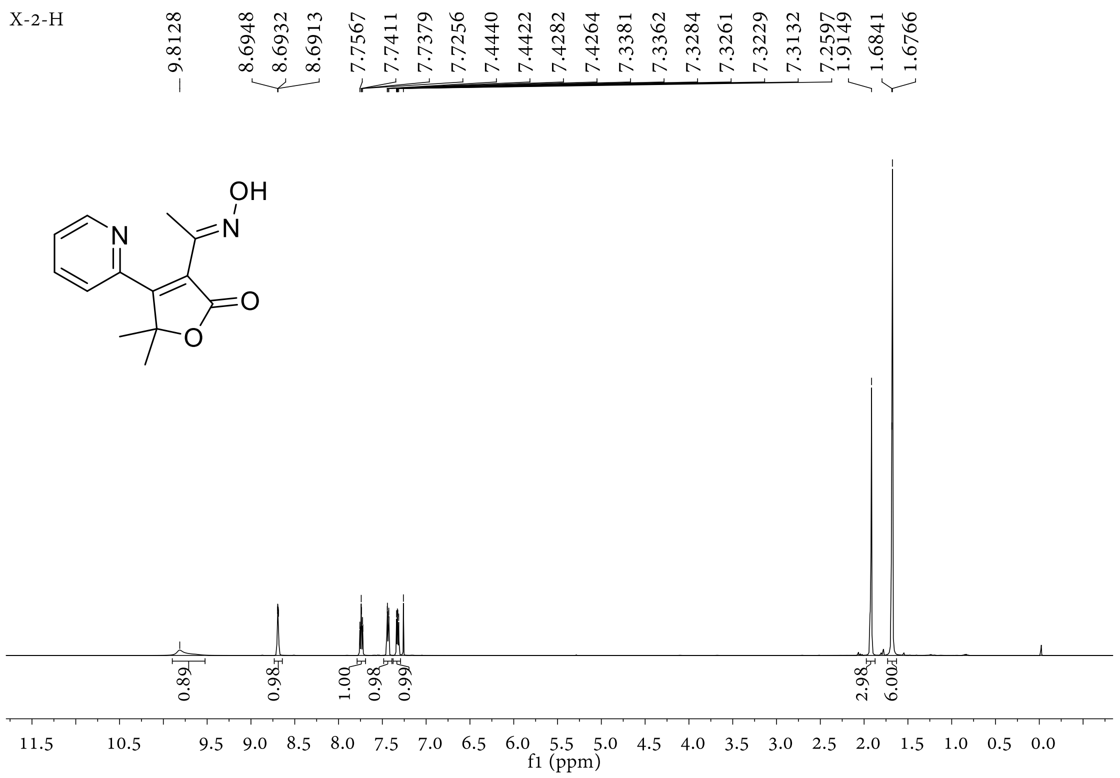

Figure S21 ^{1}H NMR of compound **IV-12** (500 MHz, $CDCl_3$)

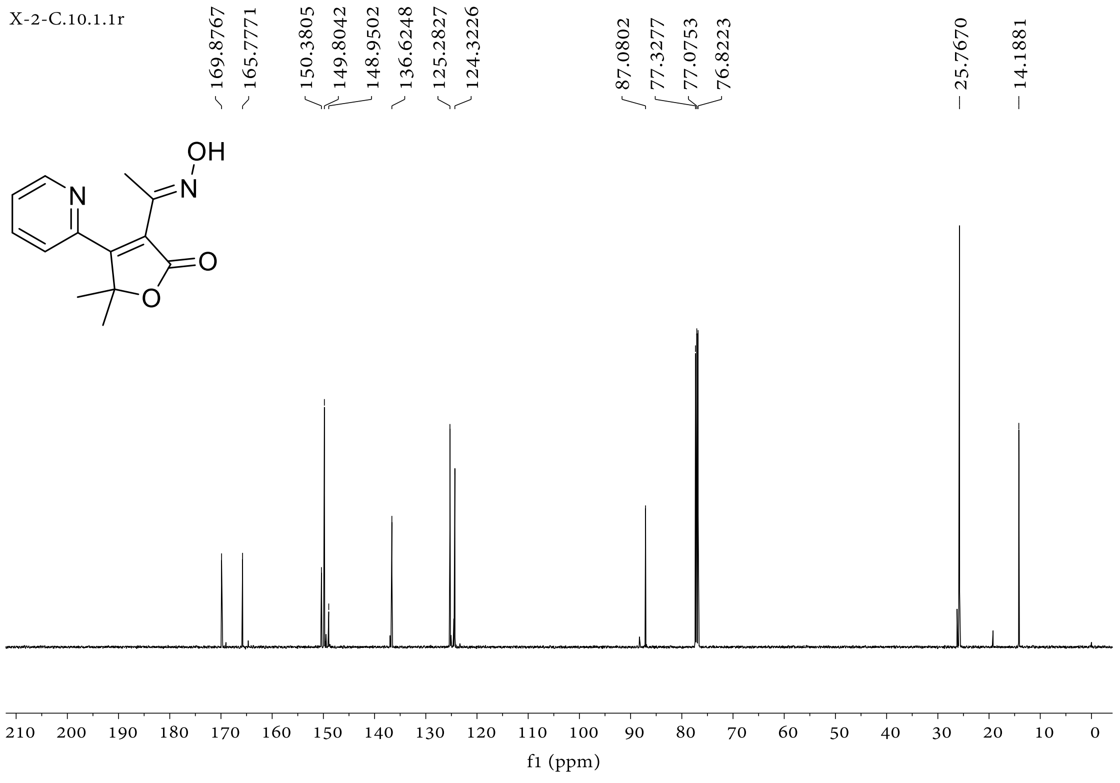

Figure S22 ^{13}C NMR of compound **IV-12** (126 MHz, $CDCl_3$)

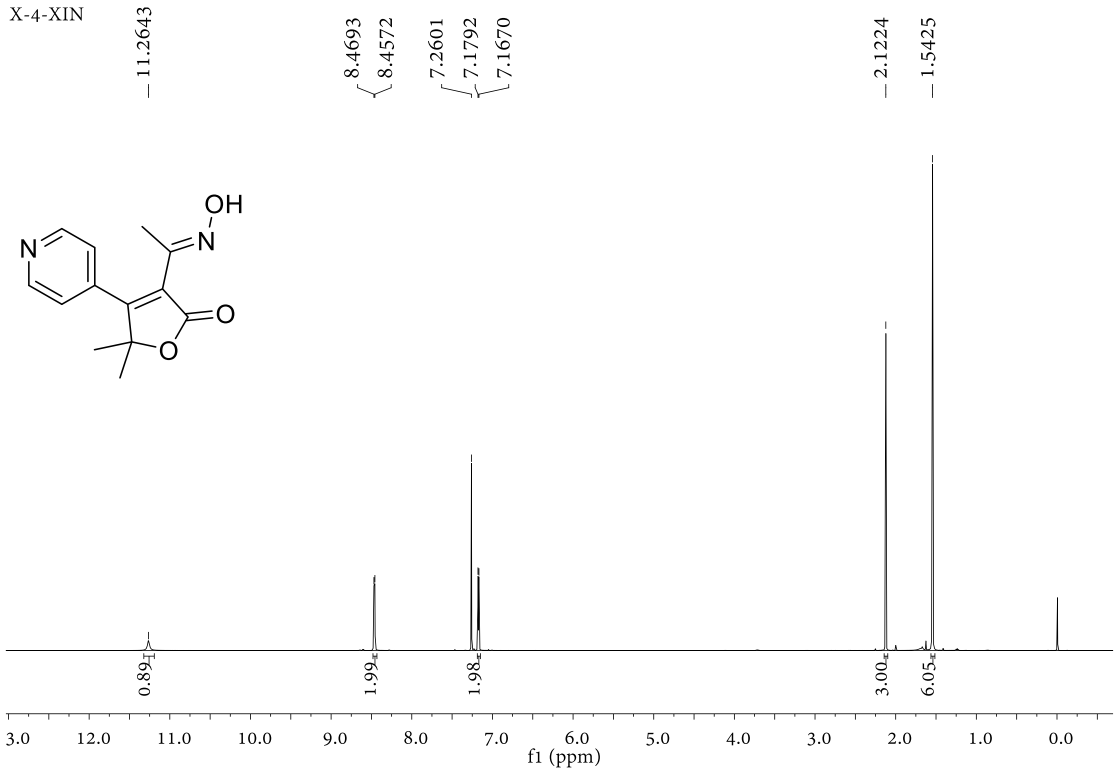

Figure S23 ^{1}H NMR of compound **IV-14** (500 MHz, $CDCl_3$)

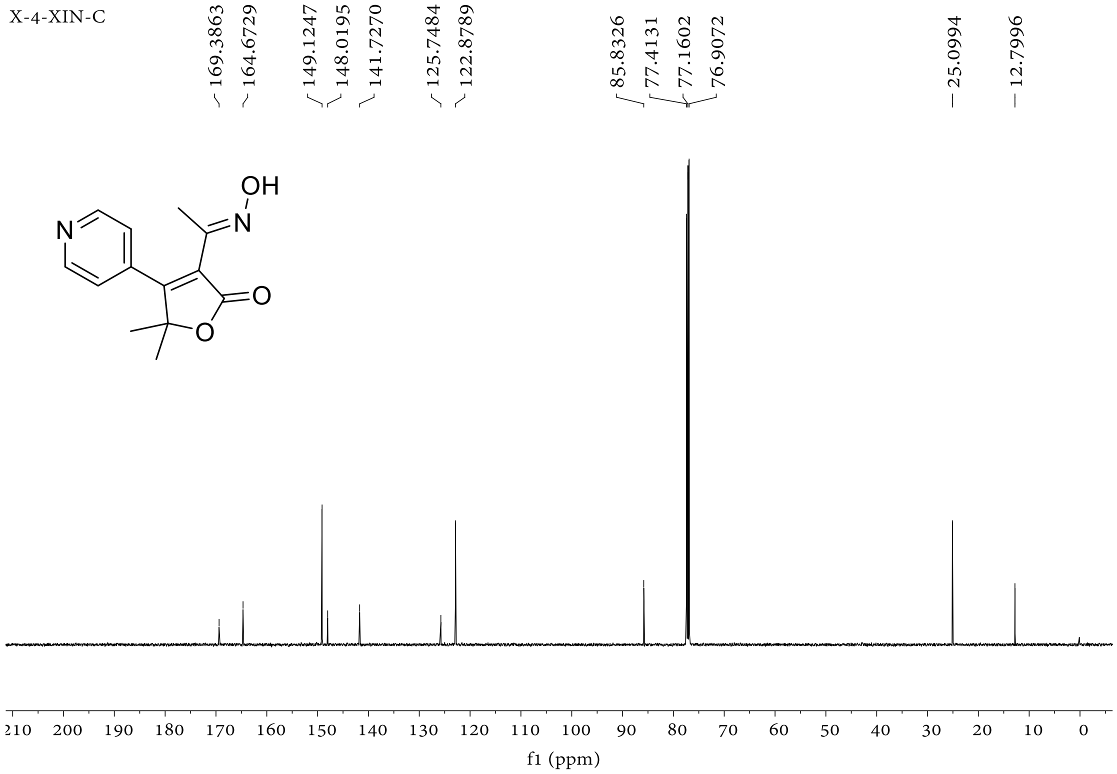

Figure S24 ^{13}C NMR of compound **IV-14** (126 MHz, $CDCl_3$)

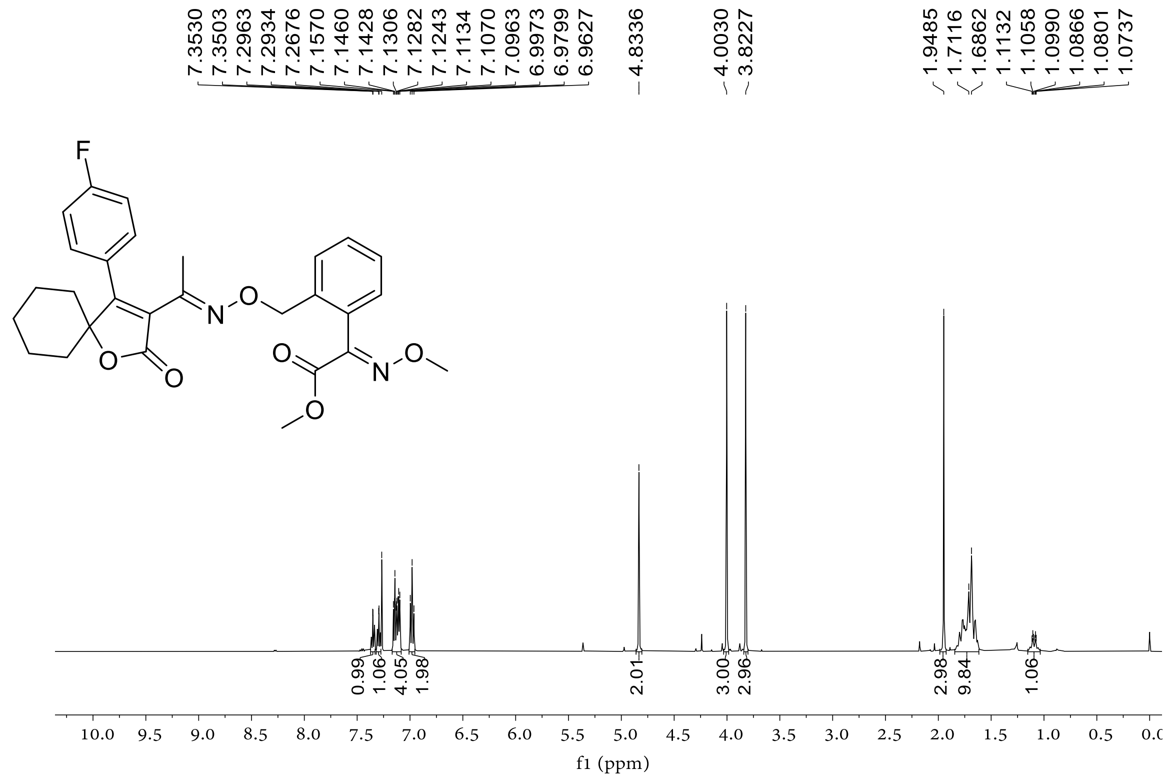

Figure S25 ^{1}H NMR of compound **V-1** (500 MHz, $CDCl_3$)

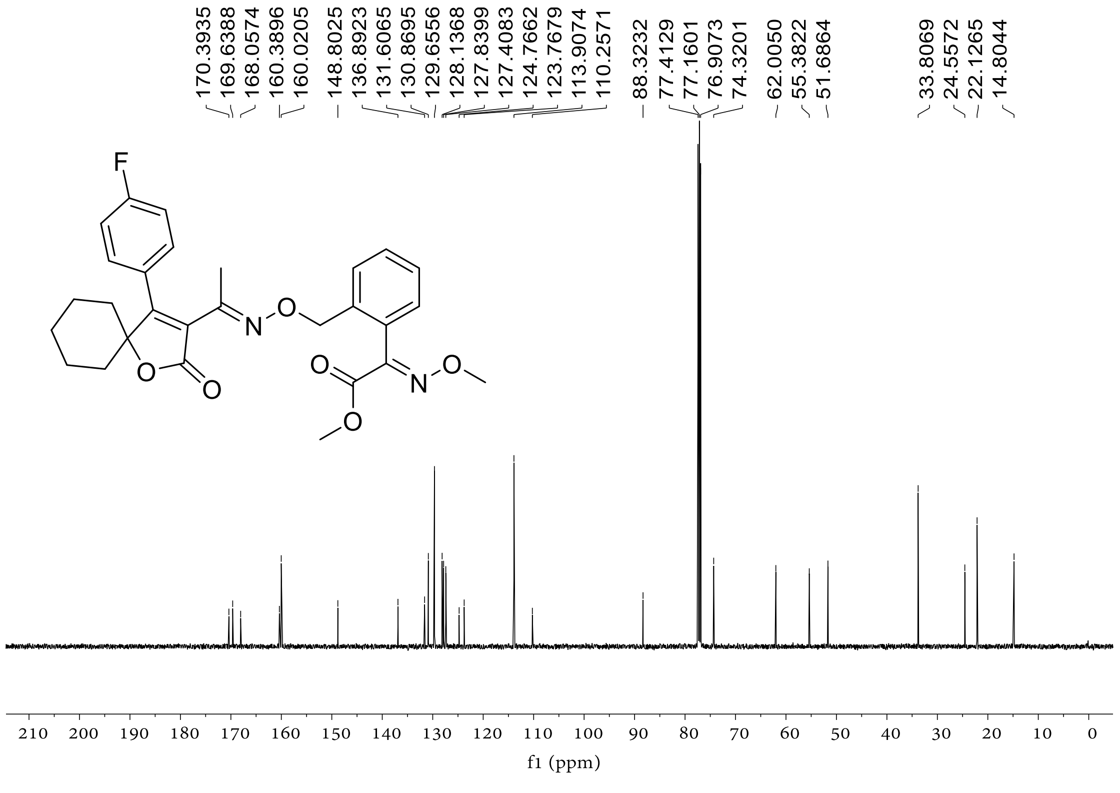

Figure S26 ^{13}C NMR of compound **V-1** (126 MHz, $CDCl_3$)

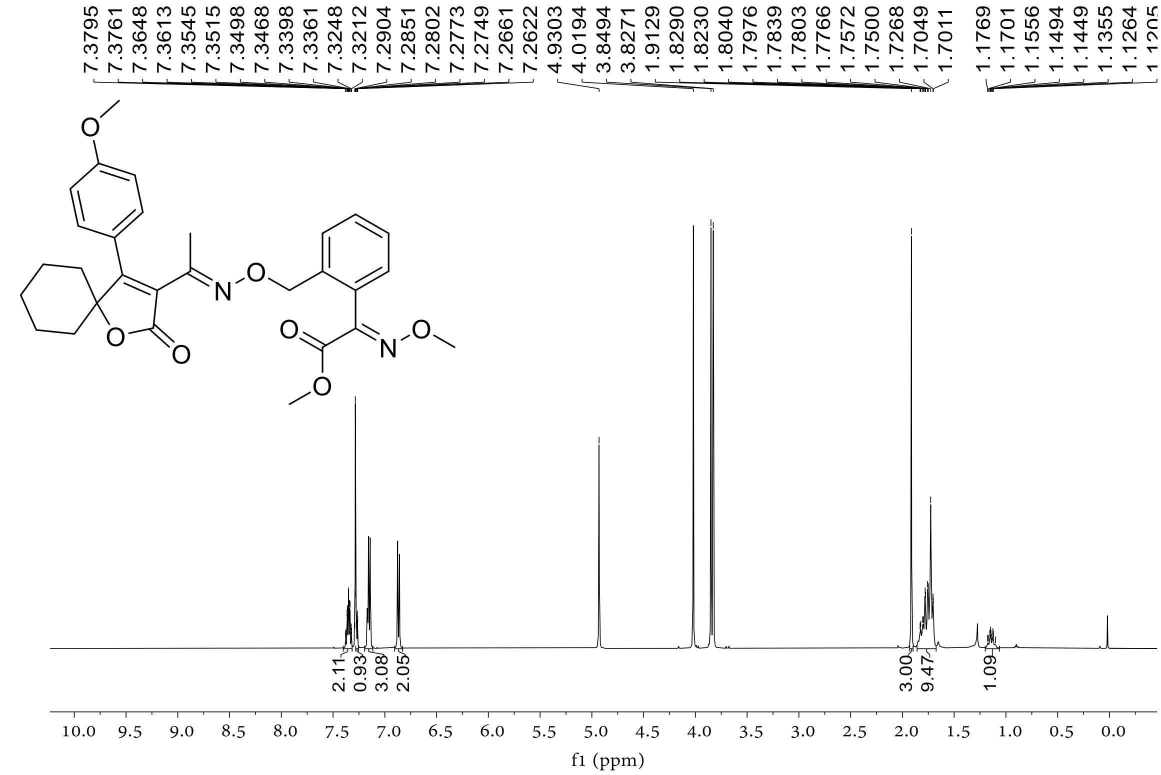

Figure S27 ^{1}H NMR of compound **V-2** (500 MHz, $CDCl_3$)

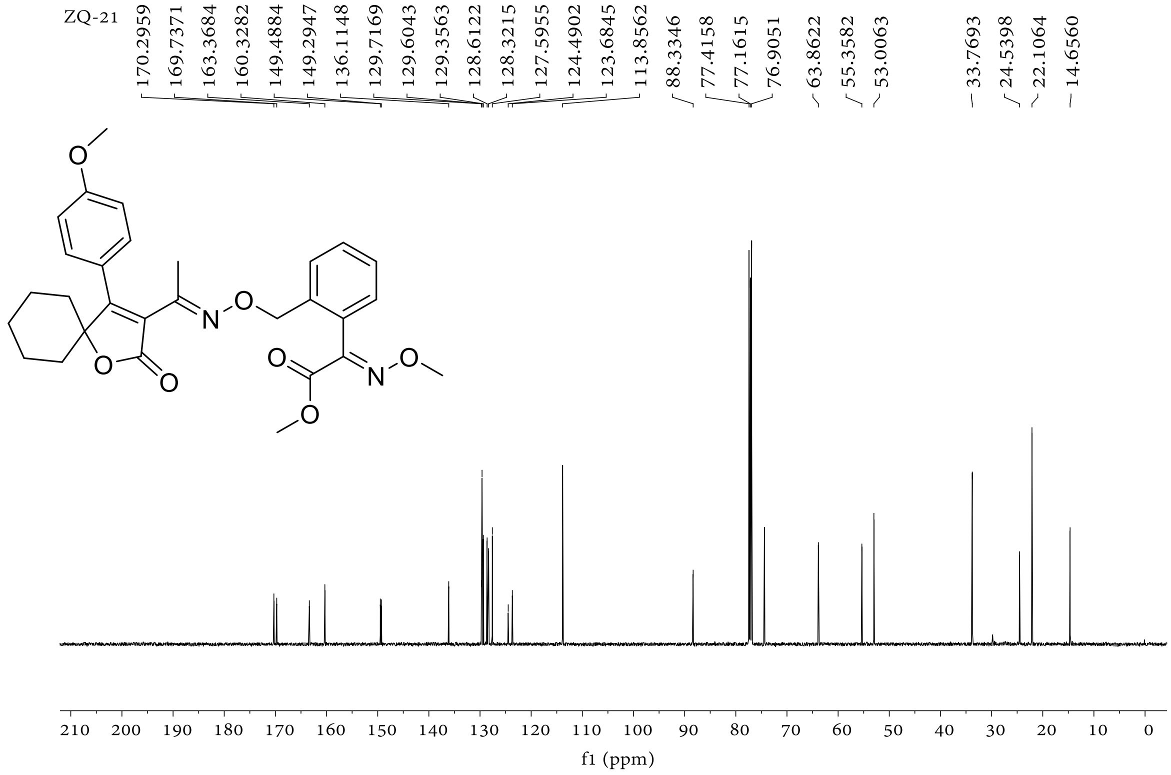

Figure S28 ^{13}C NMR of compound **V-2** (126 MHz, $CDCl_3$)

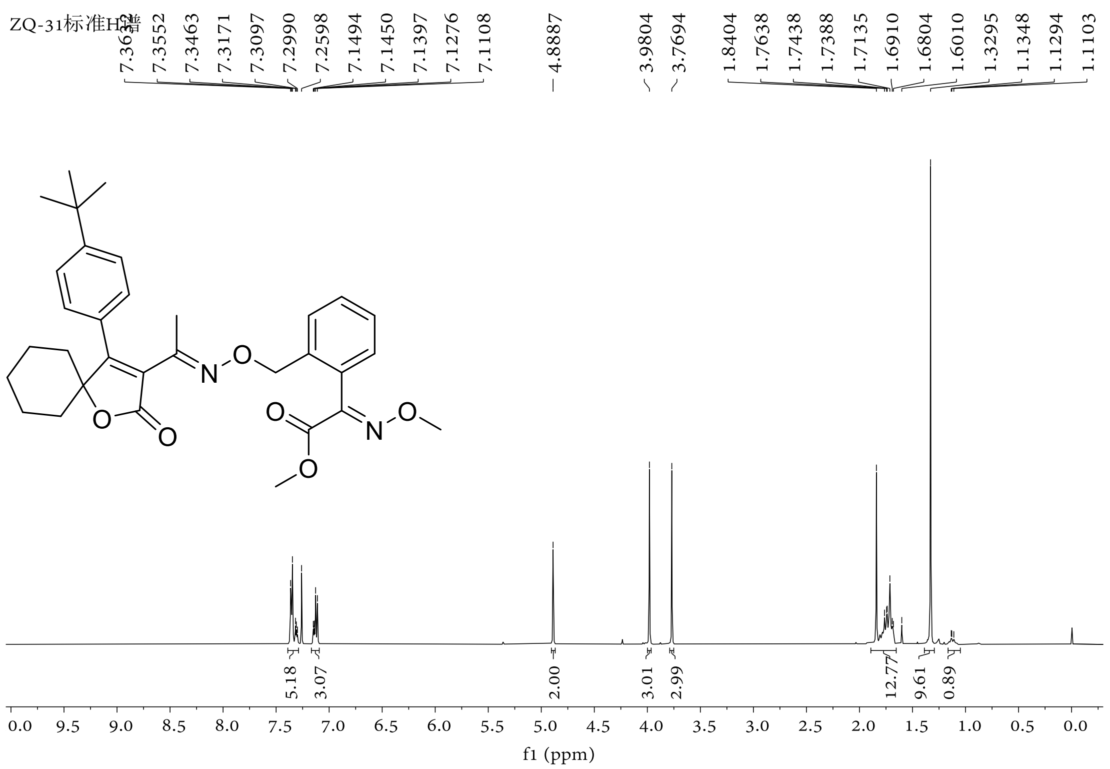

Figure S29 ^{1}H NMR of compound **V-3** (500 MHz, $CDCl_3$)

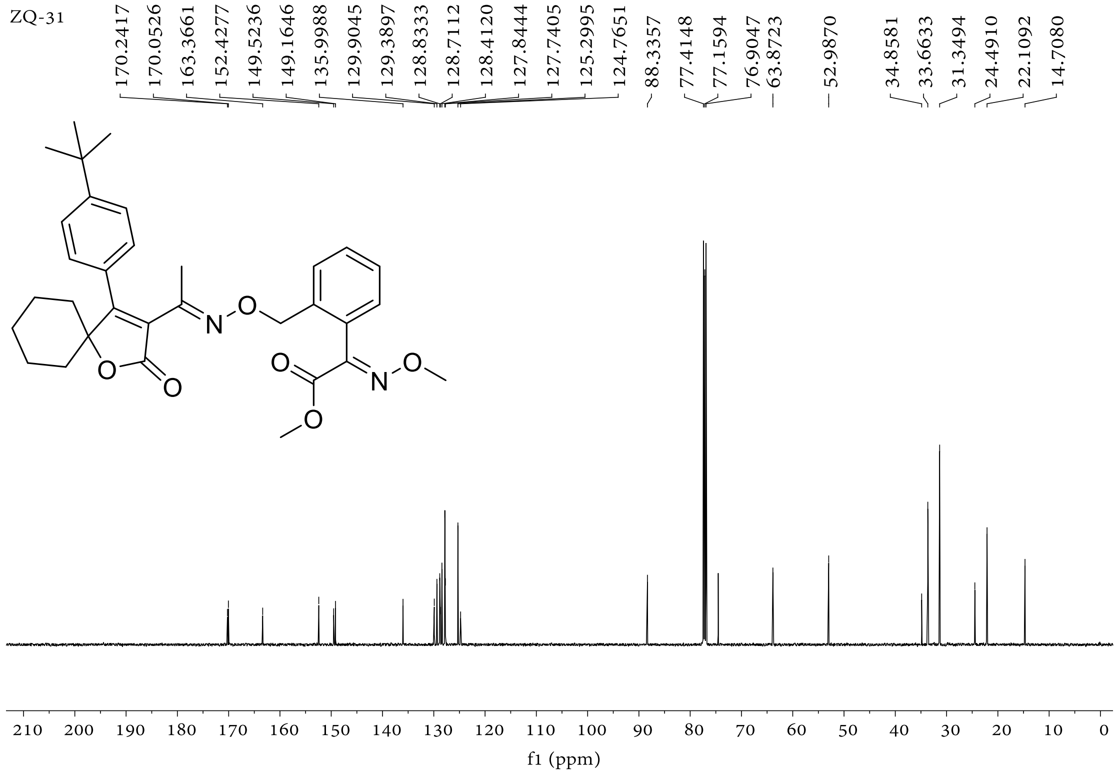

Figure S30 ^{13}C NMR of compound **V-3** (126 MHz, $CDCl_3$)

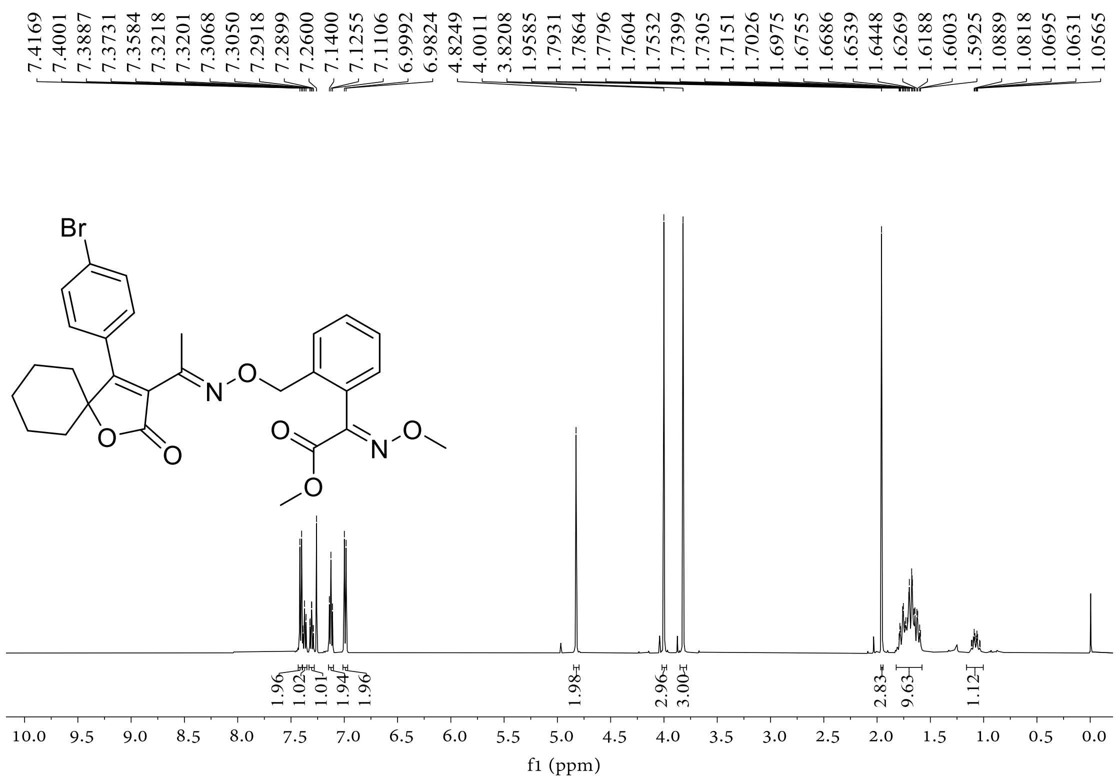

Figure S31 ^{1}H NMR of compound **V-4** (500 MHz, $CDCl_3$)

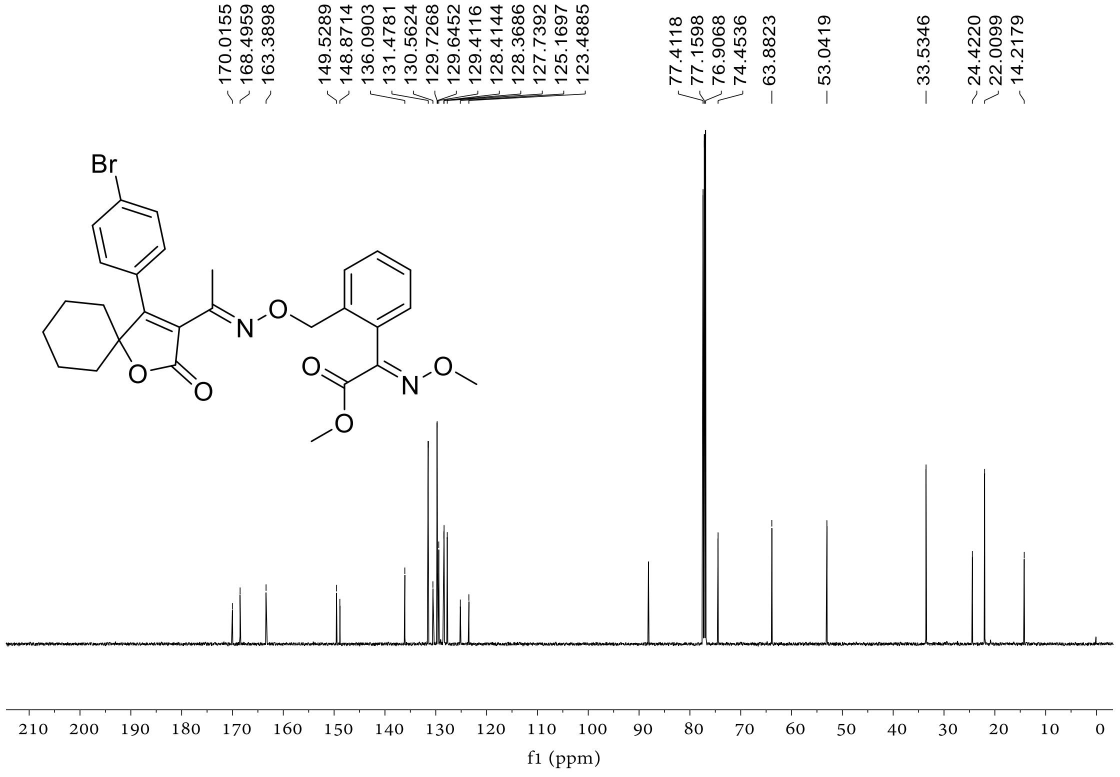

Figure S32 ^{13}C NMR of compound **V-4** (126 MHz, $CDCl_3$)

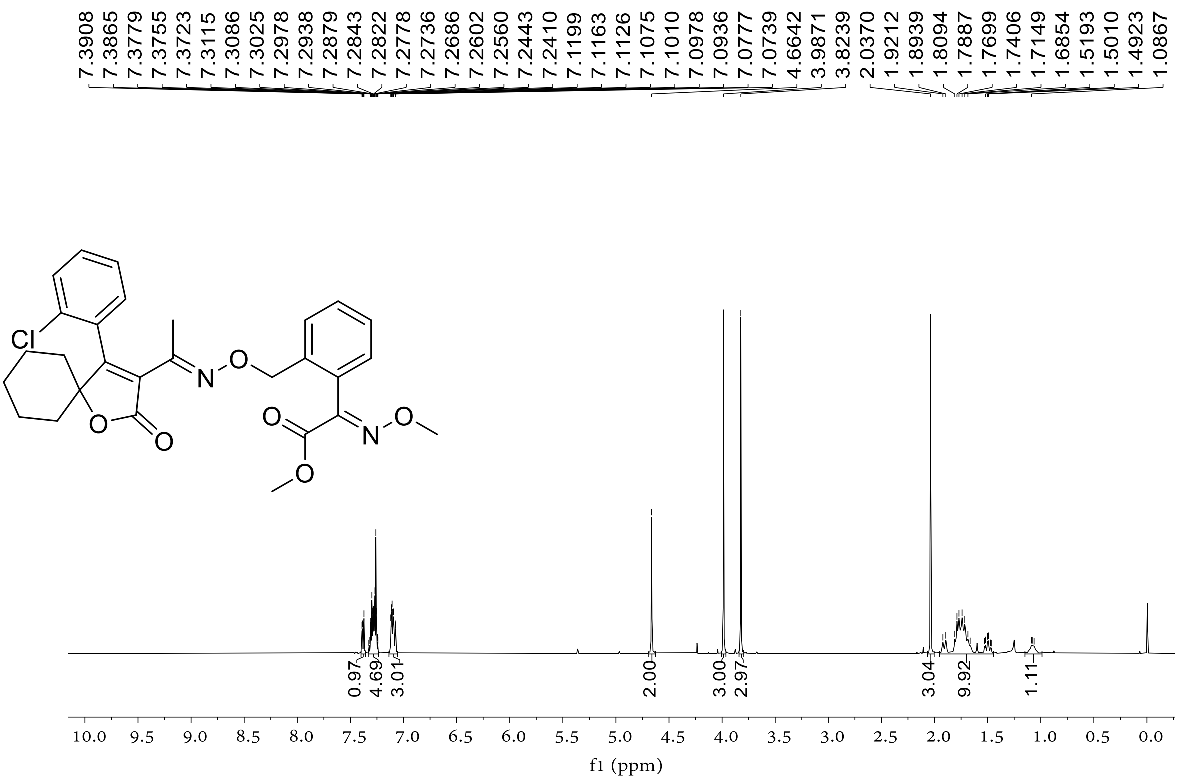

Figure S33 ^{1}H NMR of compound **V-5** (500 MHz, $CDCl_3$)

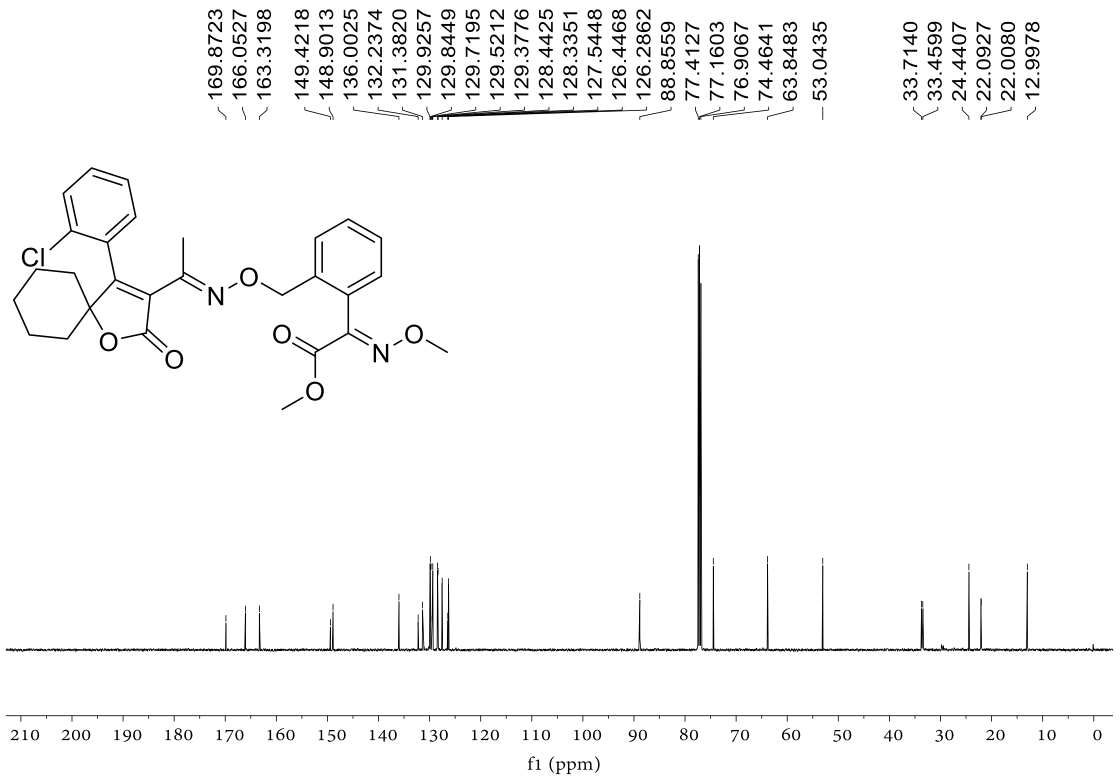

Figure S34 ^{13}C NMR of compound **V-5** (126 MHz, $CDCl_3$)

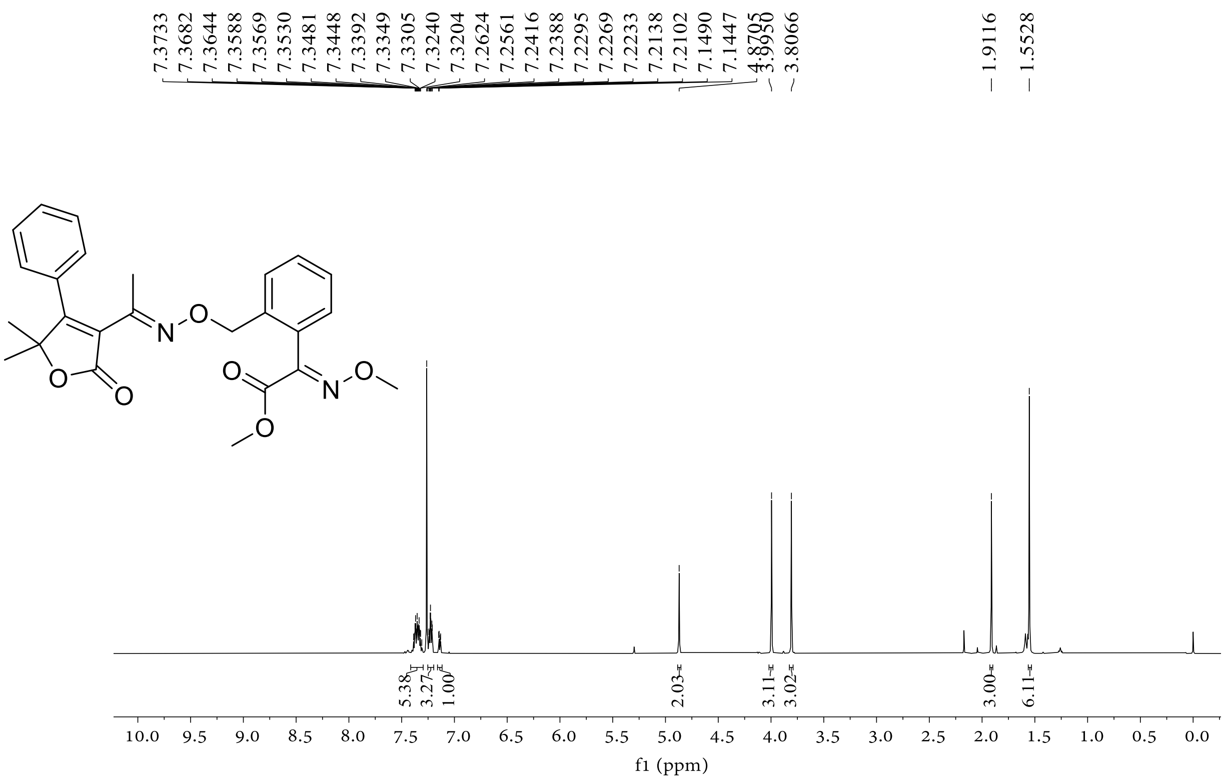

Figure S35 ^{1}H NMR of compound **V-6** (500 MHz, $CDCl_3$)

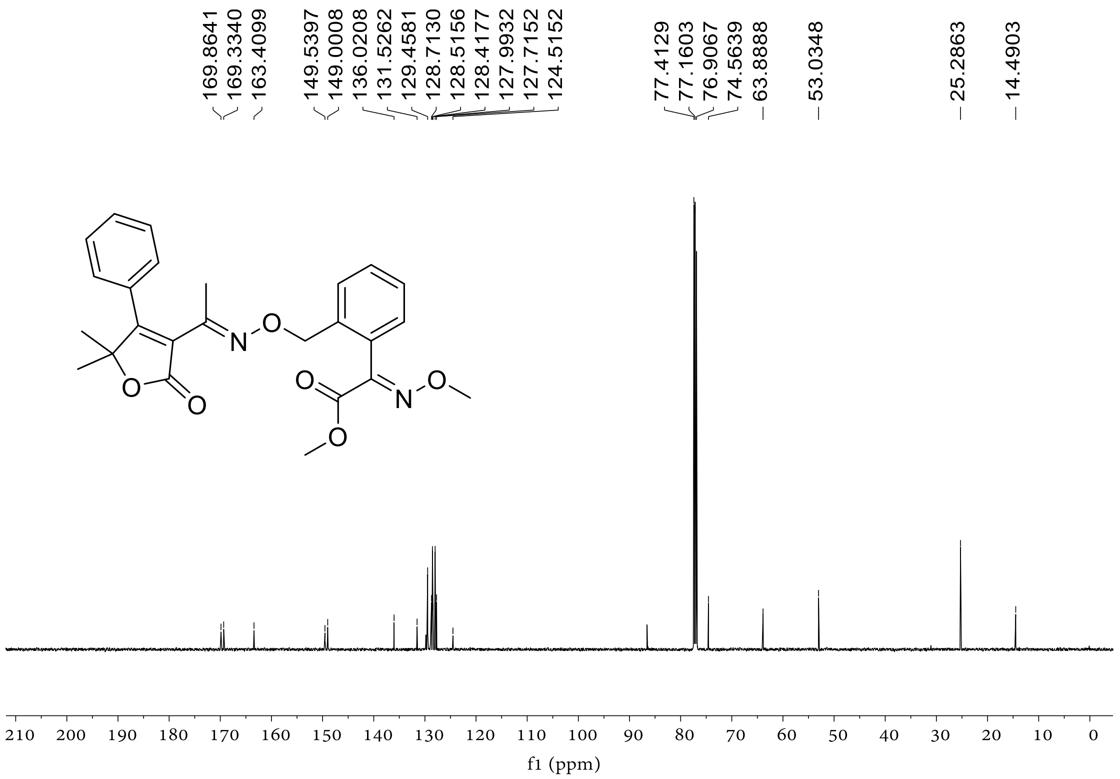

Figure S36 ^{13}C NMR of compound **V-6** (126 MHz, $CDCl_3$)

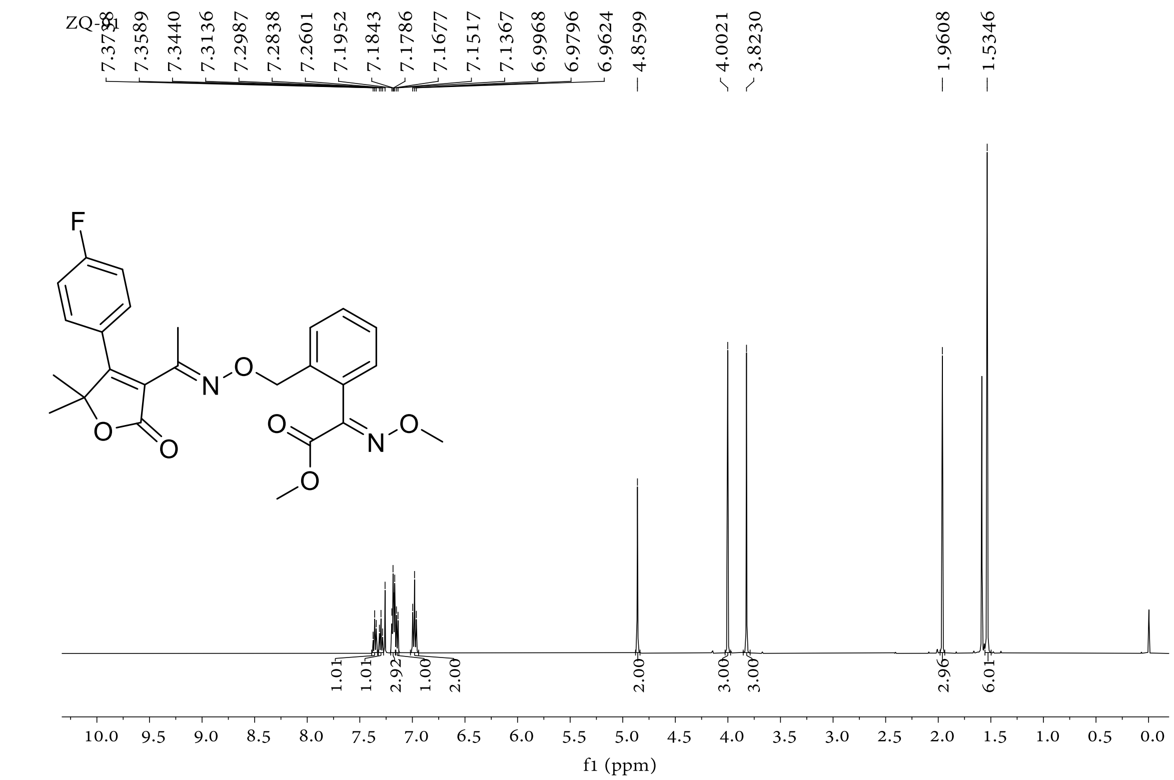

Figure S37 ^{1}H NMR of compound **V-7** (500 MHz, $CDCl_3$)

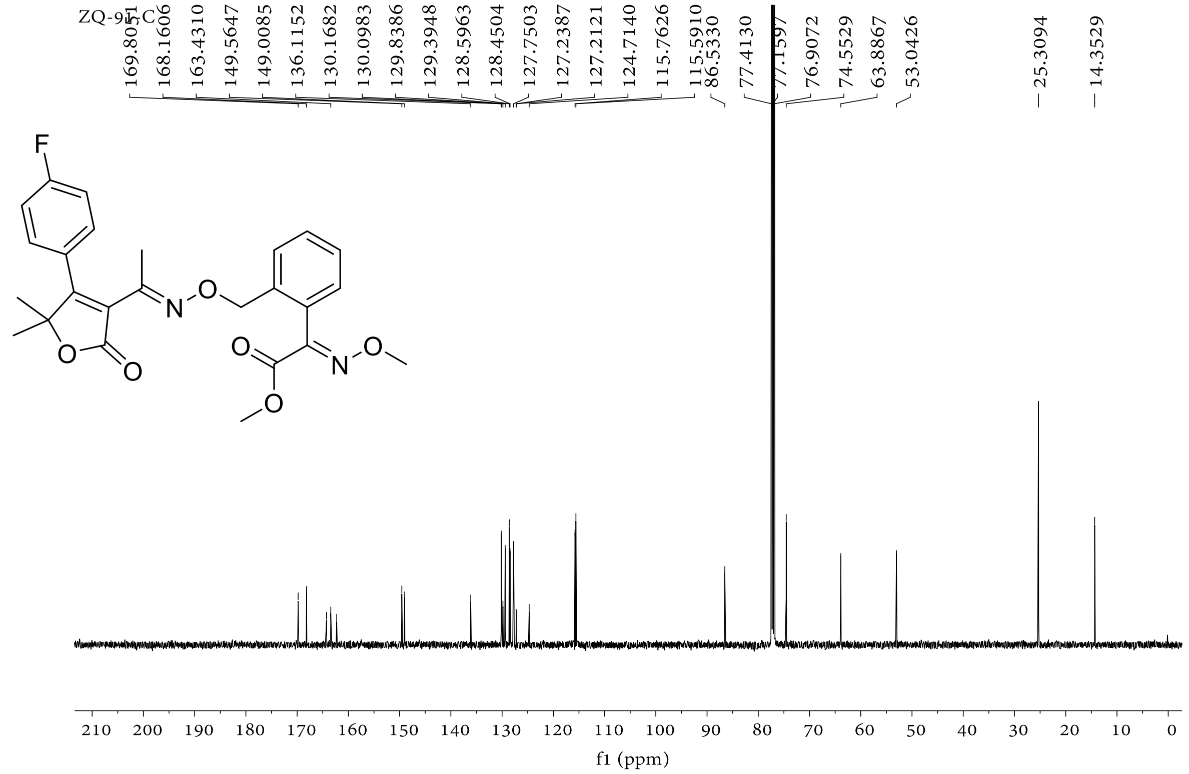

Figure S38 ^{13}C NMR of compound **V-7** (126 MHz, $CDCl_3$)

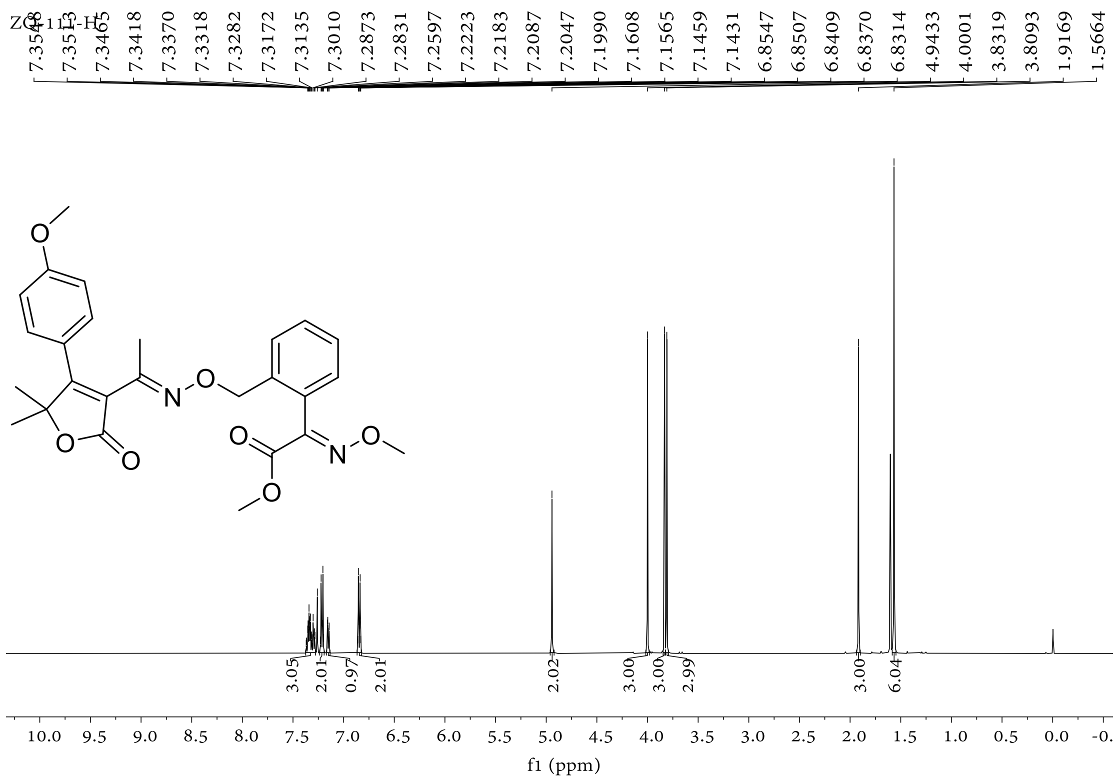

Figure S39 ^{1}H NMR of compound **V-8** (500 MHz, $CDCl_3$)

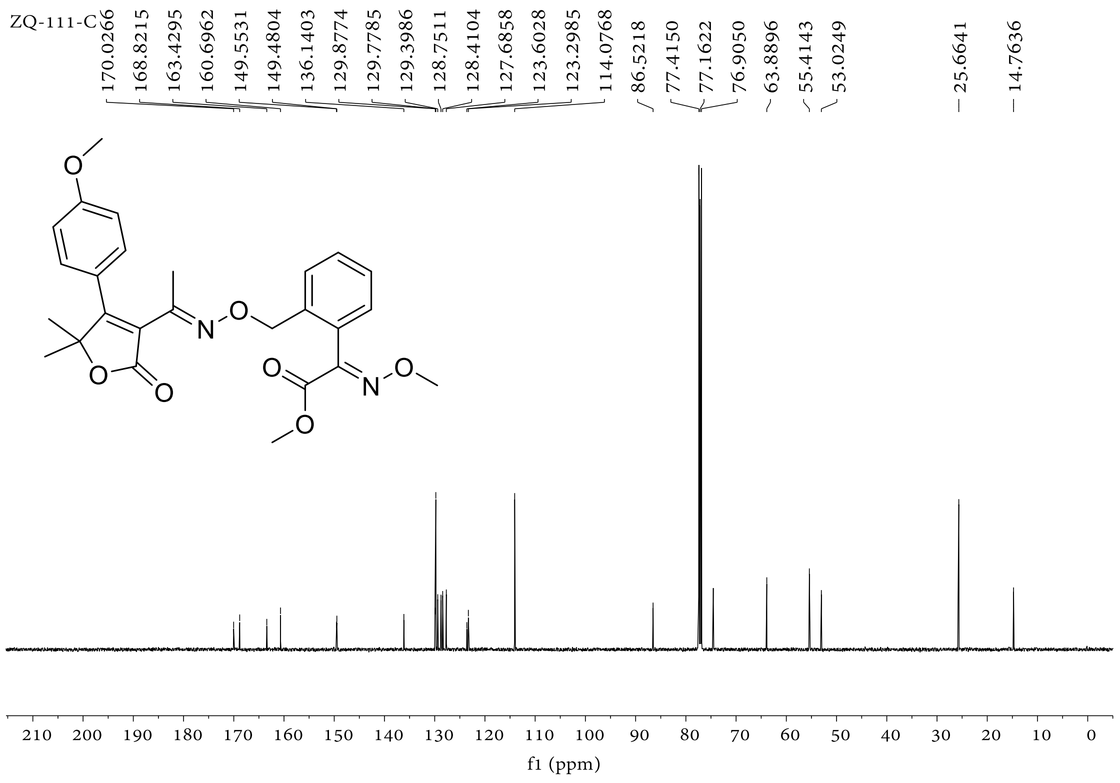

Figure S40 ^{13}C NMR of compound **V-8** (126 MHz, $CDCl_3$)

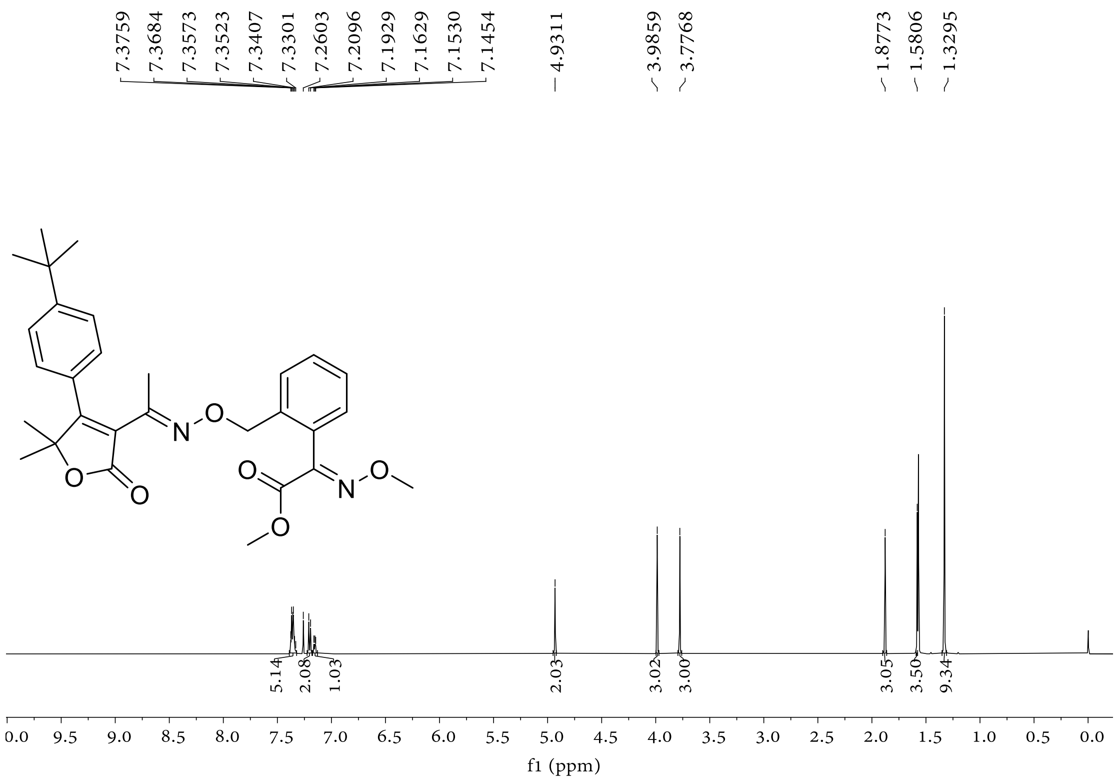

Figure S41 ¹H NMR of compound **V-9** (500 MHz, $CDCl_3$)

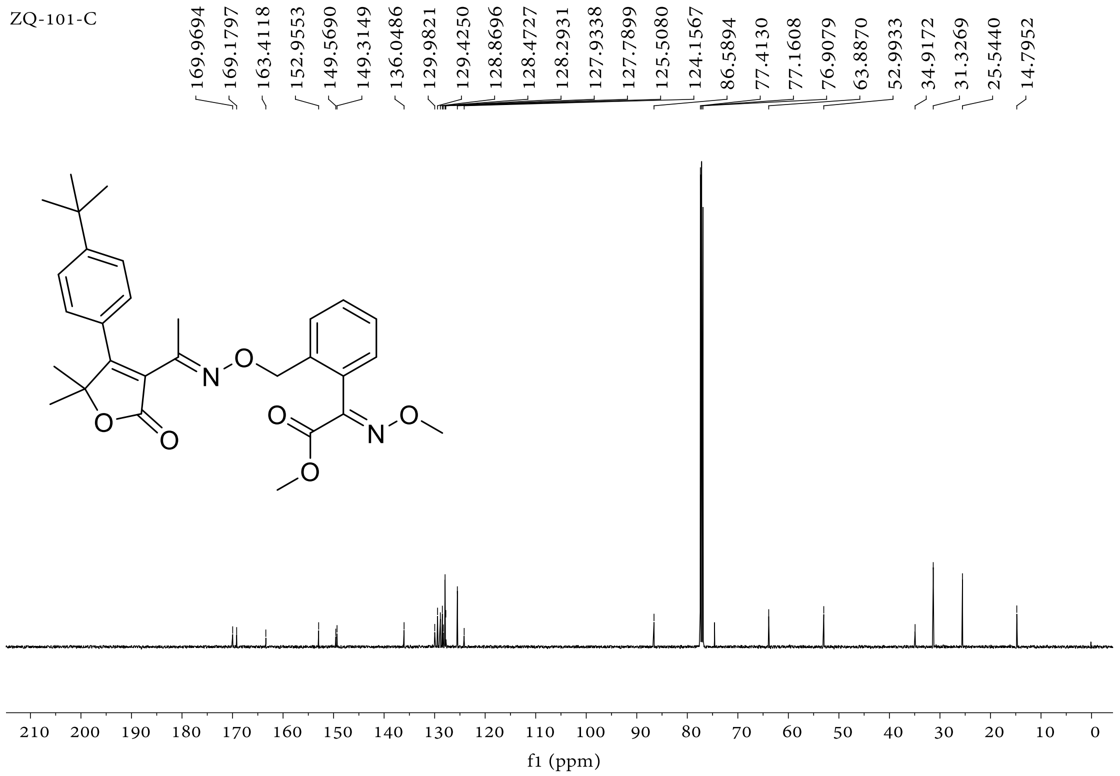

Figure S42 ¹³C NMR of compound **V-9** (126 MHz, $CDCl_3$)

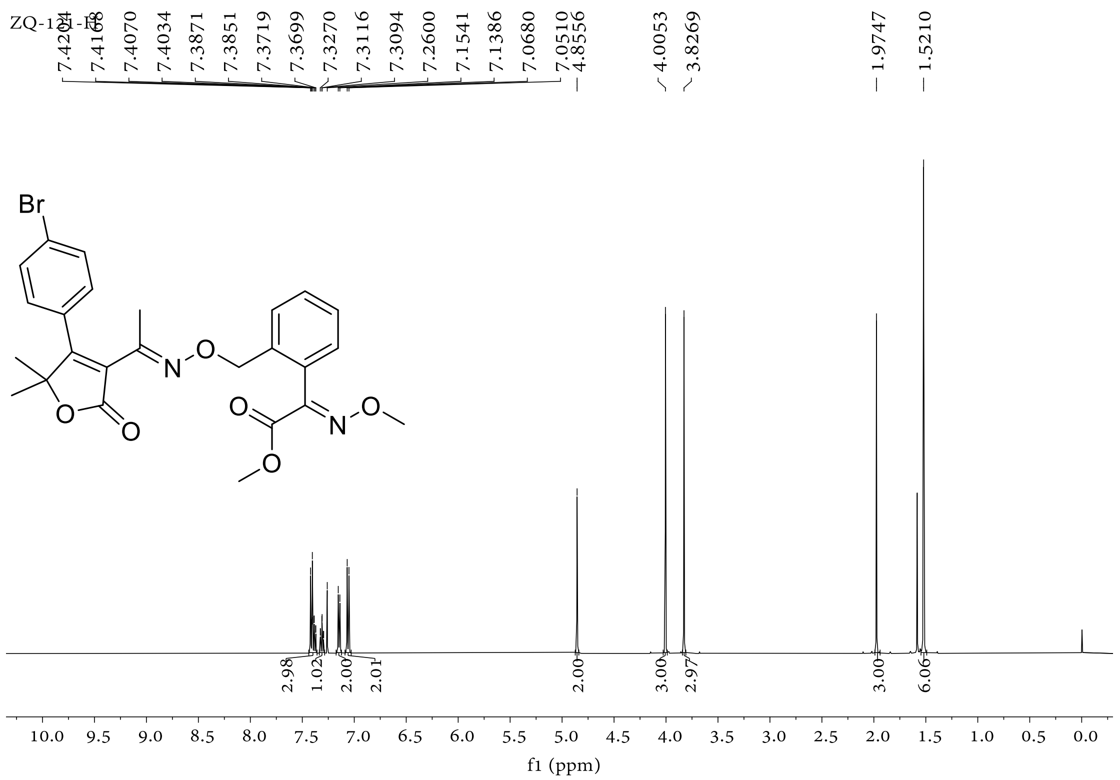

Figure S43 ^{1}H NMR of compound **V-10** (500 MHz, $CDCl_3$)

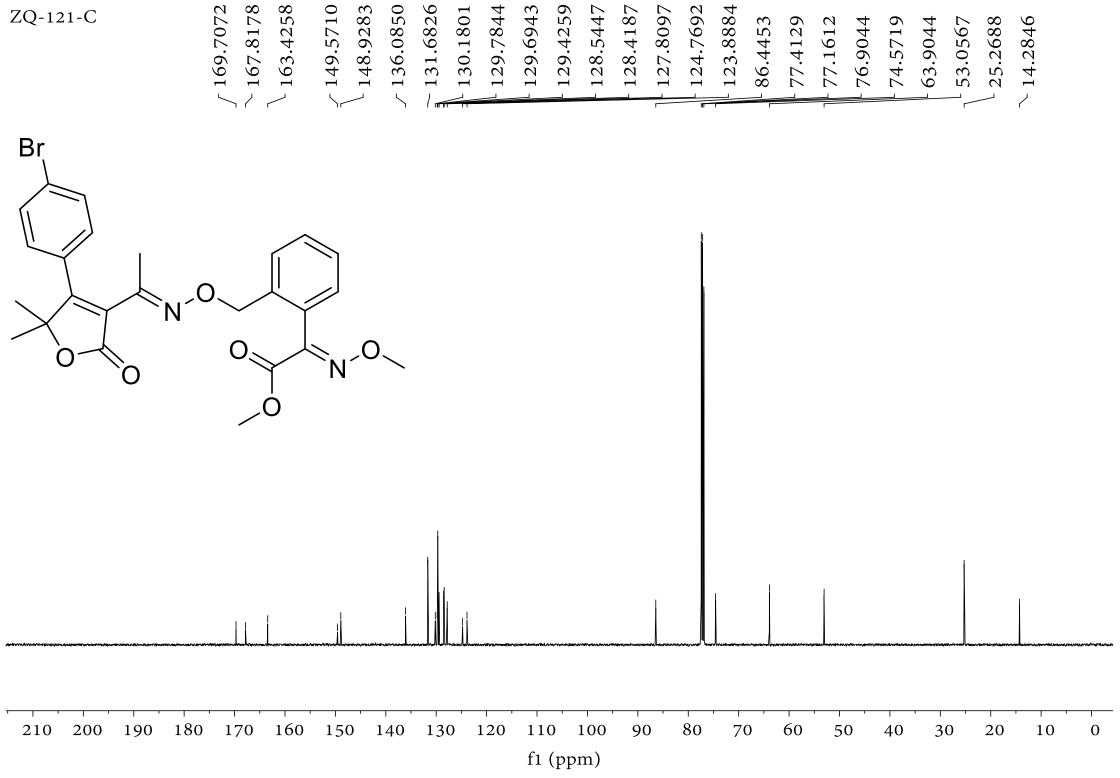

Figure S44 ^{13}C NMR of compound **V-10** (126 MHz, $CDCl_3$)

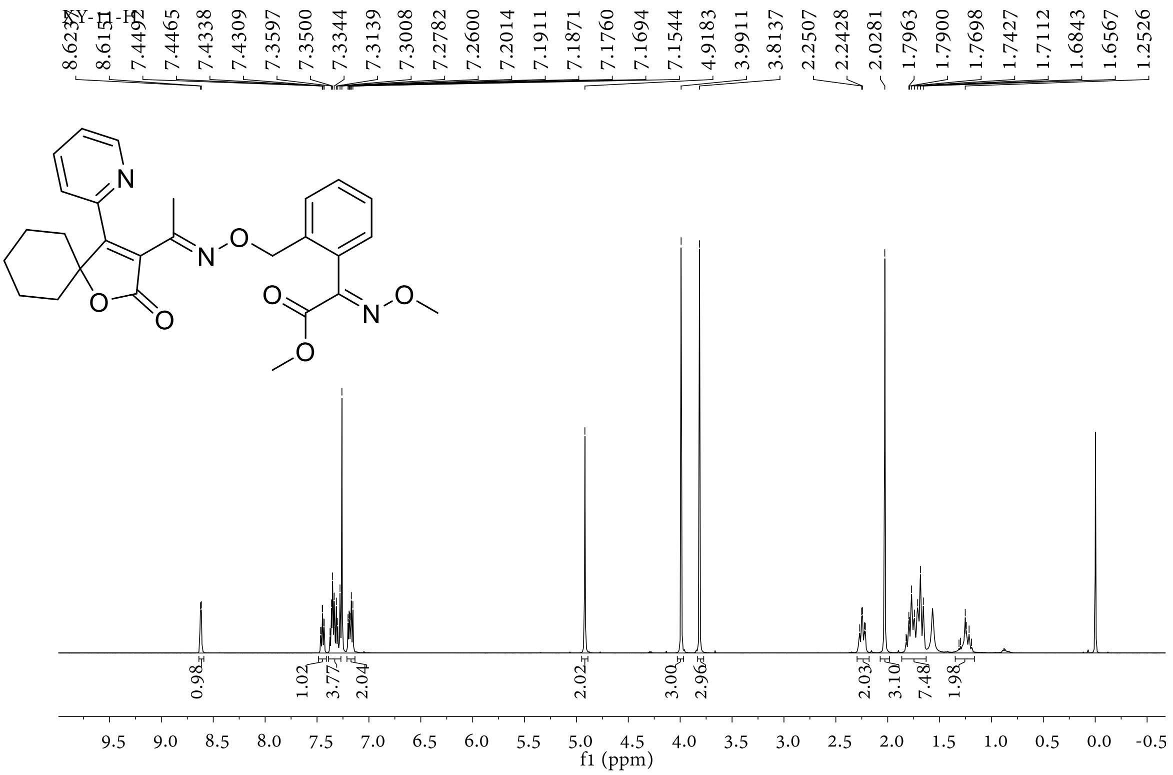

Figure S45 ^{1}H NMR of compound **V-11** (500 MHz, $CDCl_3$)

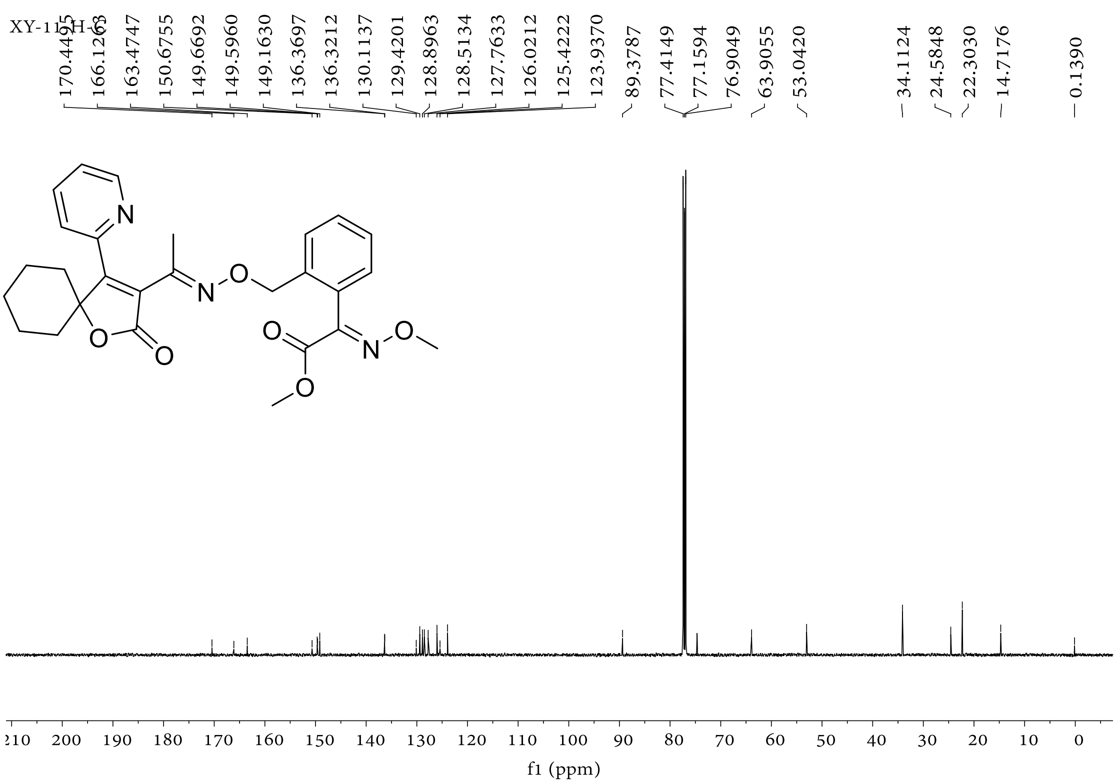

Figure S46 ^{13}C NMR of compound **V-11** (126 MHz, $CDCl_3$)

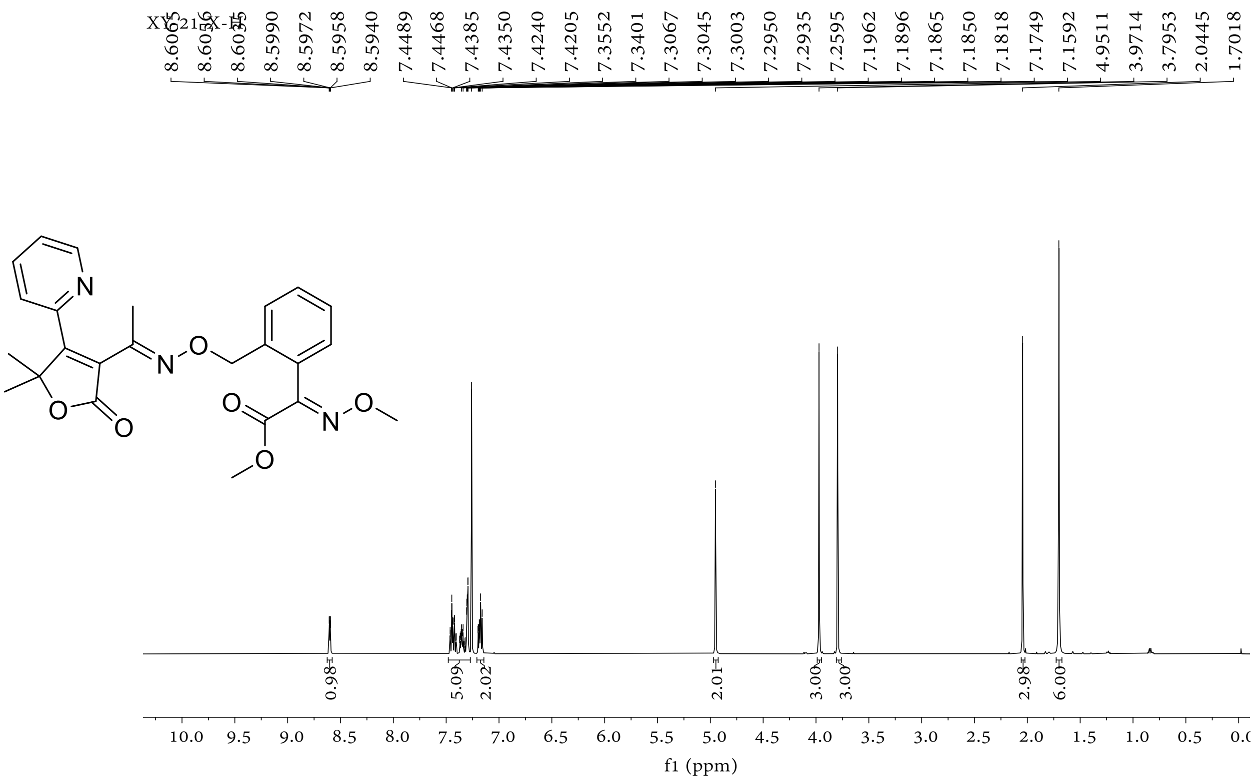

Figure S47 ^{1}H NMR of compound **V-12** (500 MHz, $CDCl_3$)

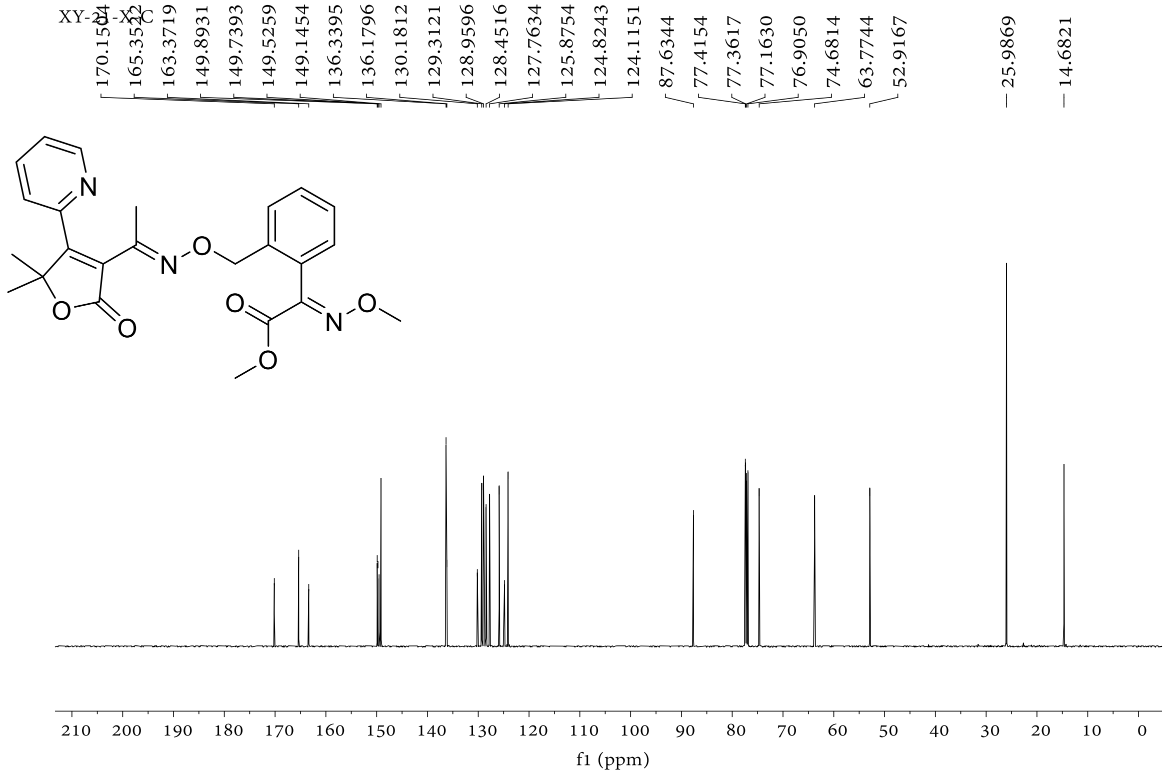

Figure S48 ^{13}C NMR of compound **V-12** (126 MHz, $CDCl_3$)

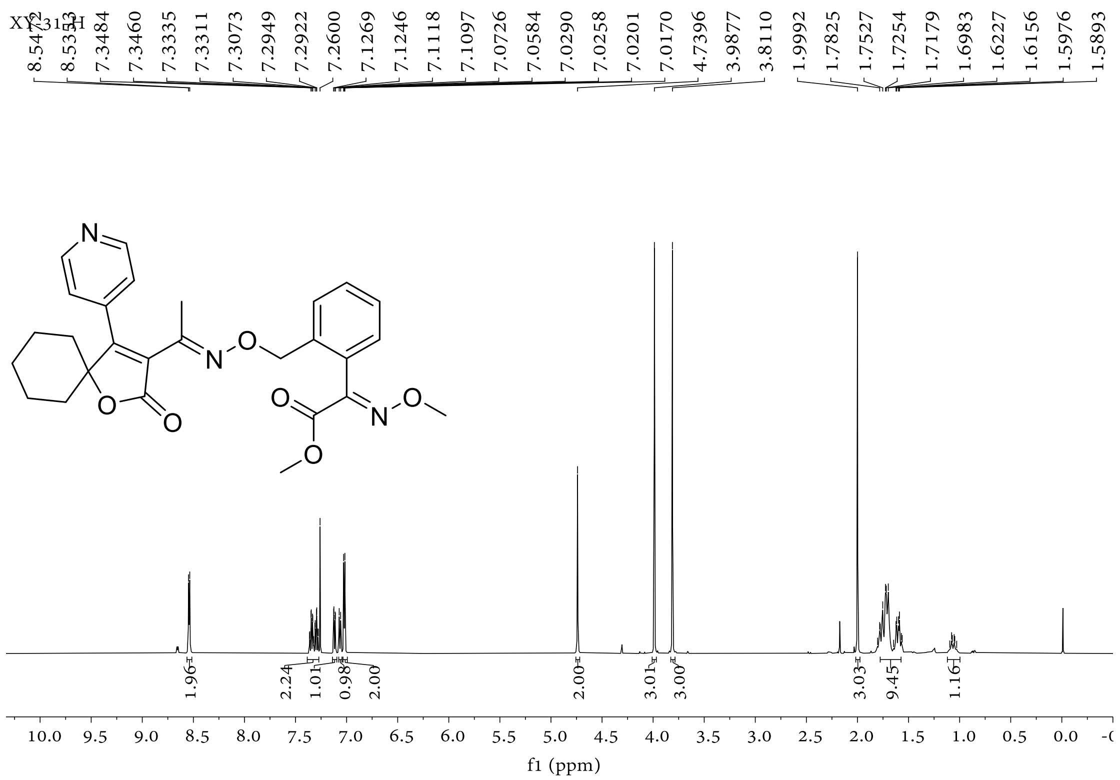

Figure S49 ^{1}H NMR of compound **V-13** (500 MHz, CDCl$_3$)

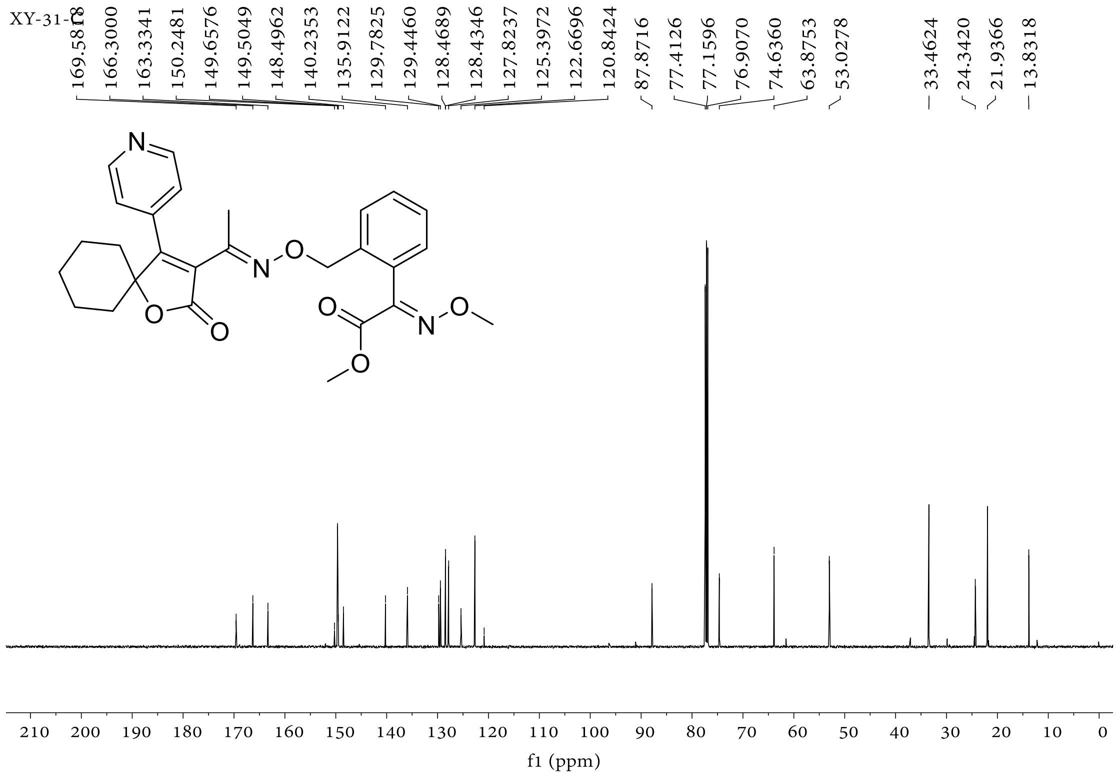

Figure S50 ^{13}C NMR of compound **V-13** (126 MHz, CDCl$_3$)

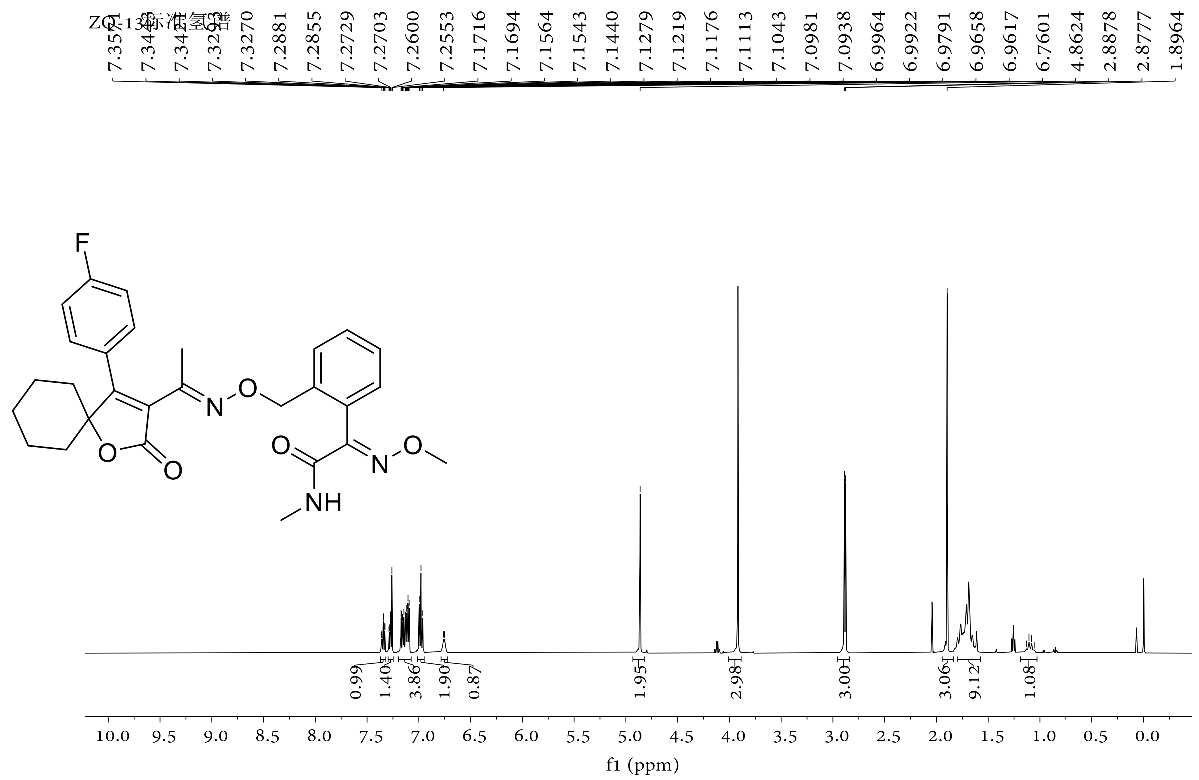

Figure S51 ^{1}H NMR of compound **VI-1** (500 MHz, $CDCl_3$)

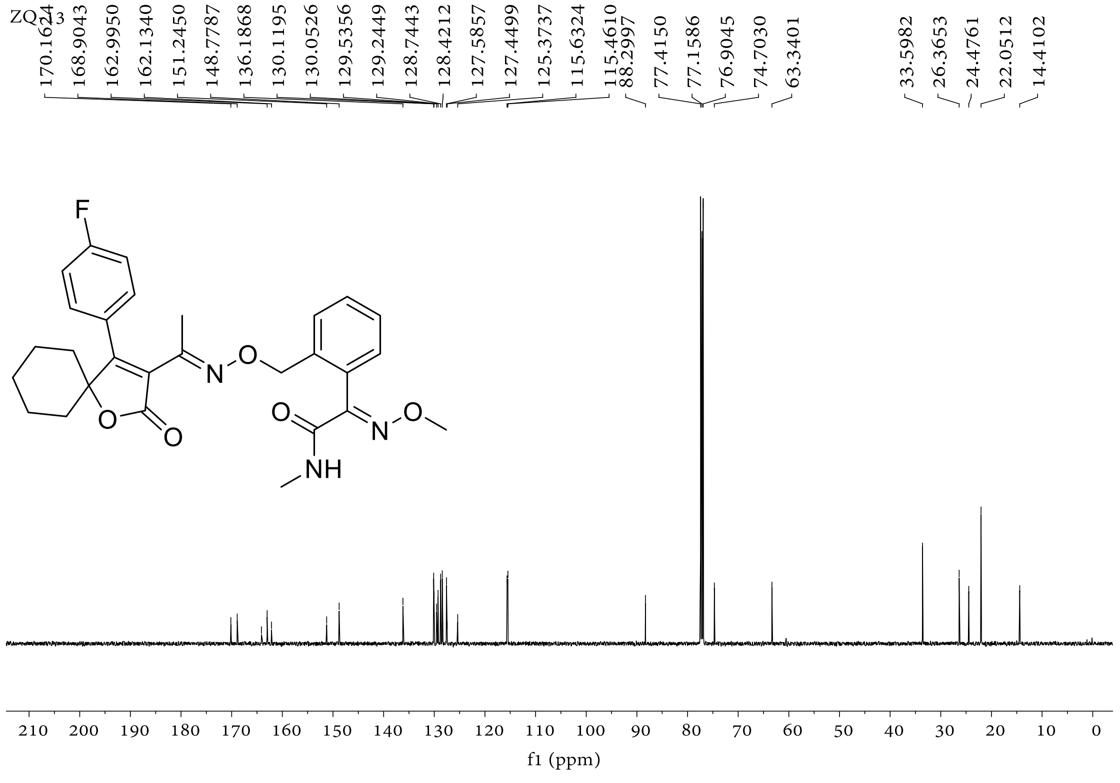

Figure S52 ^{13}C NMR of compound **VI-1** (126 MHz, $CDCl_3$)

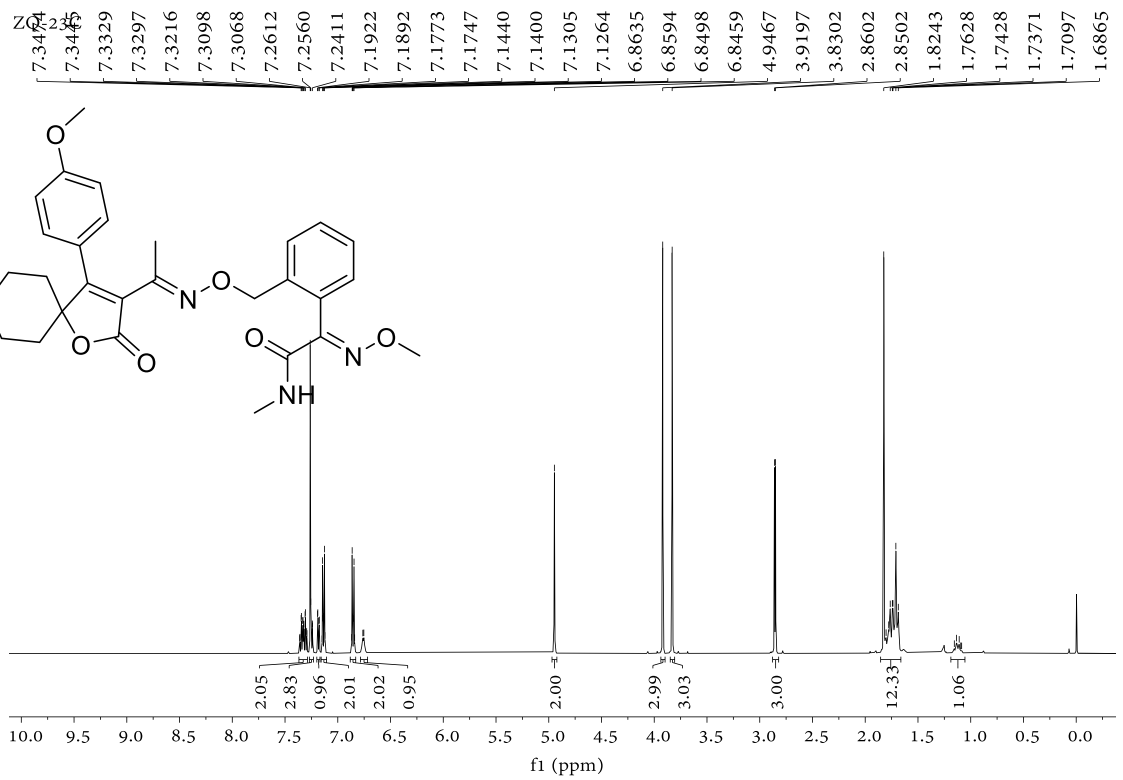

Figure S53 ^{1}H NMR of compound **VI-2** (500 MHz, $CDCl_3$)

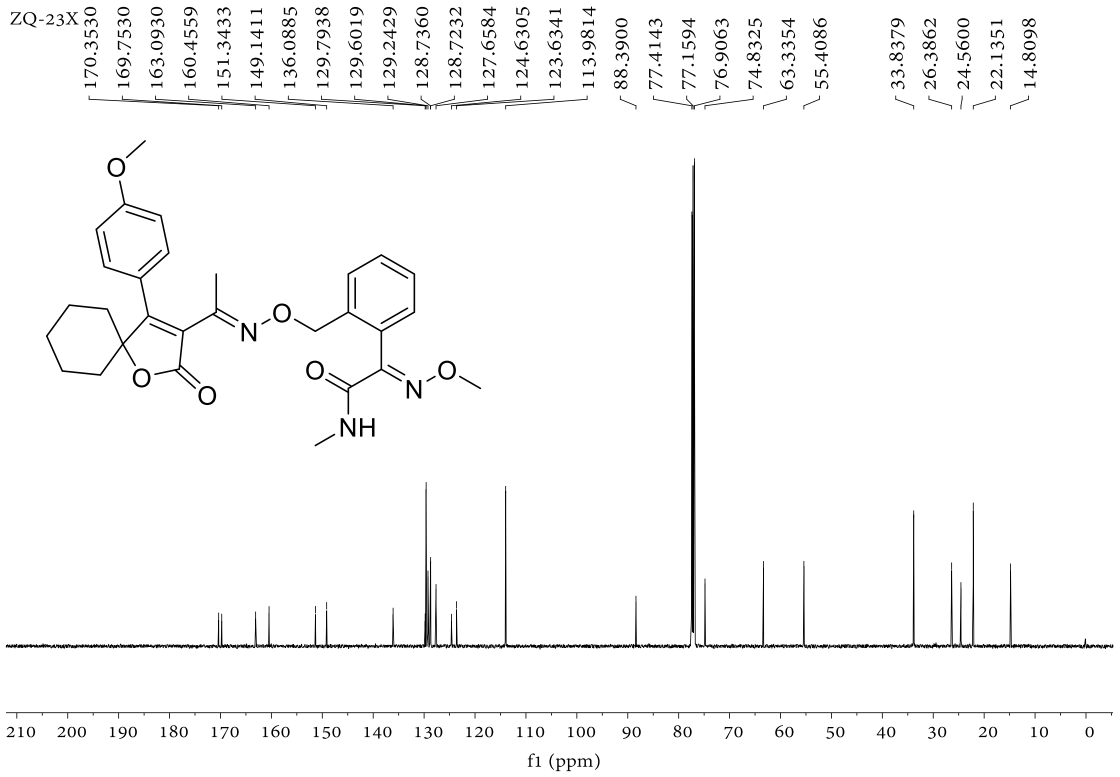

Figure S54 ^{13}C NMR of compound **VI-2** (126 MHz, $CDCl_3$)

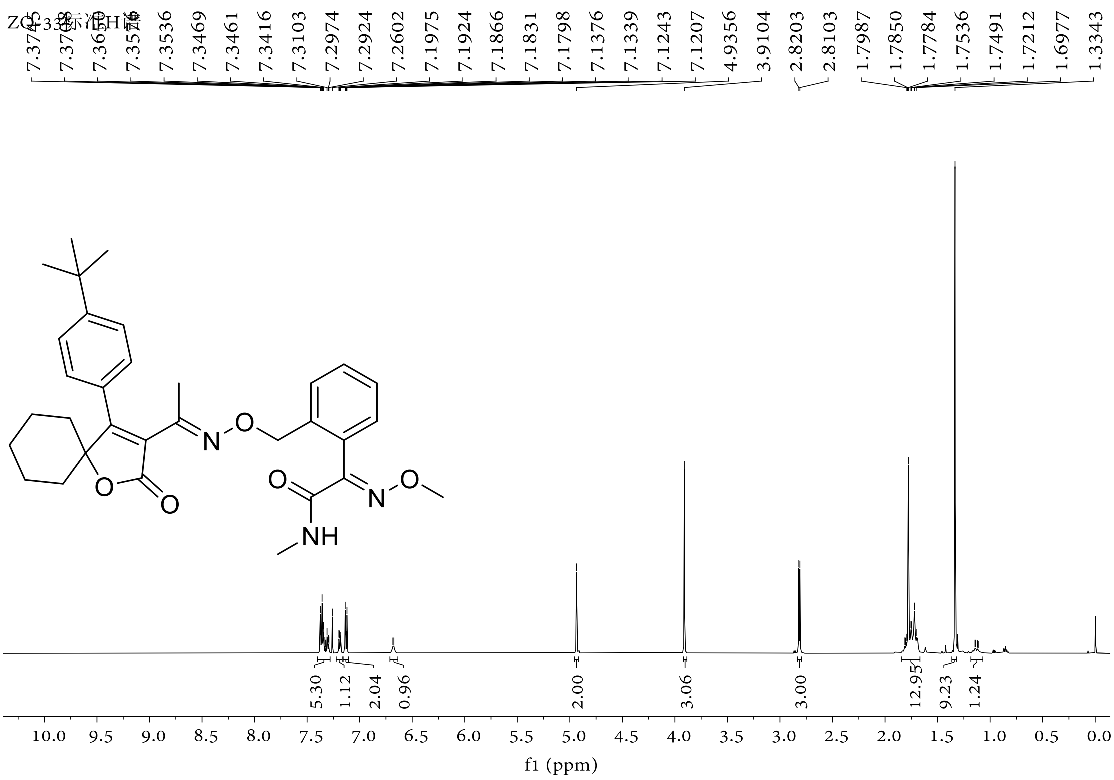

Figure S55 ^{1}H NMR of compound **VI-3** (500 MHz, $CDCl_3$)

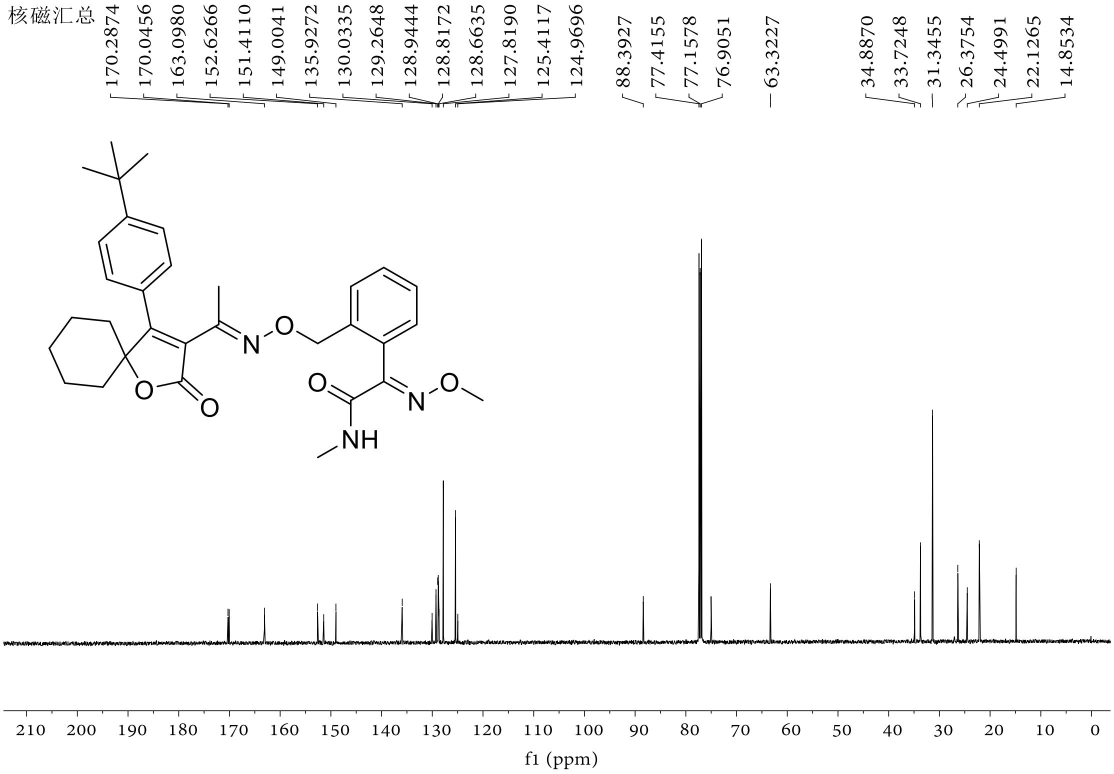

Figure S56 ^{13}C NMR of compound **VI-3** (126 MHz, $CDCl_3$)

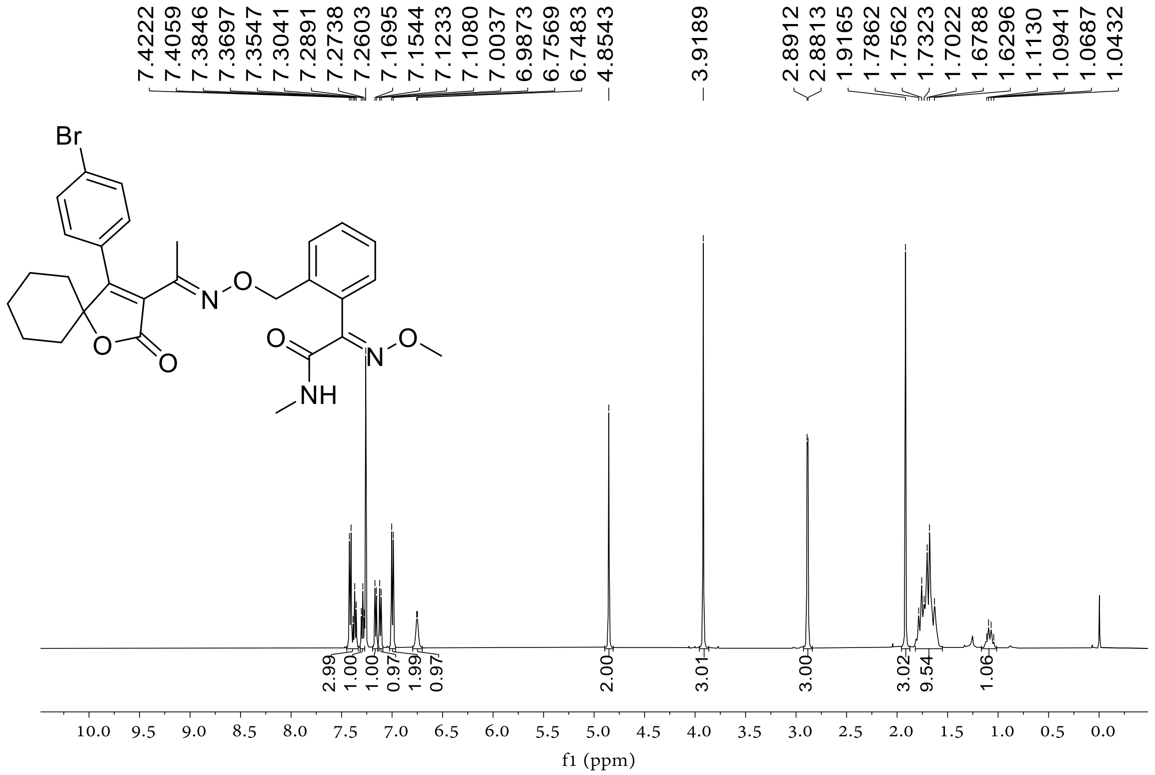

Figure S57 ^{1}H NMR of compound **VI-4** (500 MHz, $CDCl_3$)

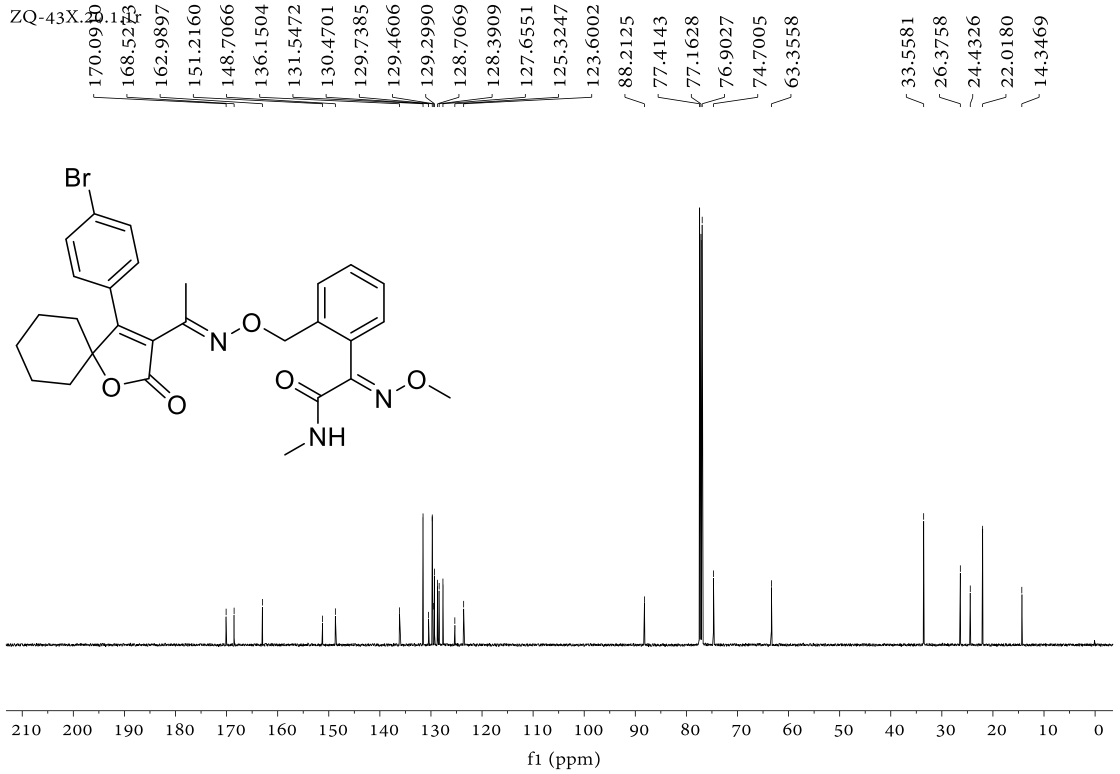

Figure S58 ^{13}C NMR of compound **VI-4** (126 MHz, $CDCl_3$)

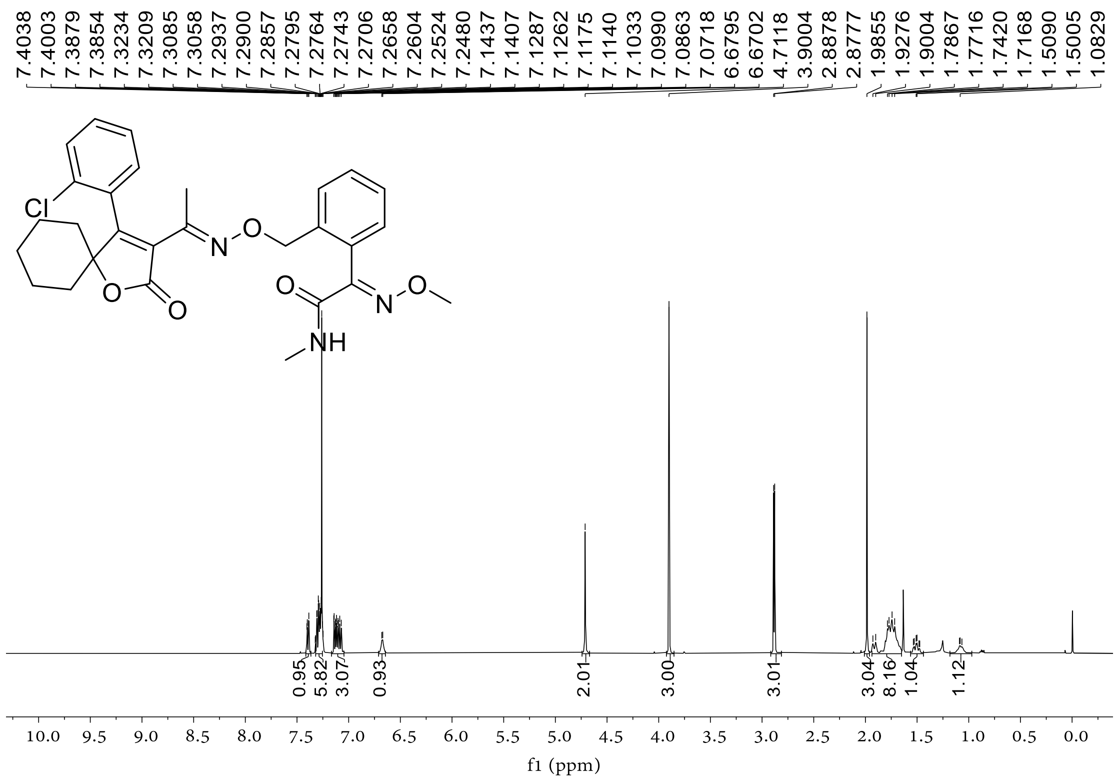

Figure S59 ^{1}H NMR of compound **VI-5** (500 MHz, $CDCl_3$)

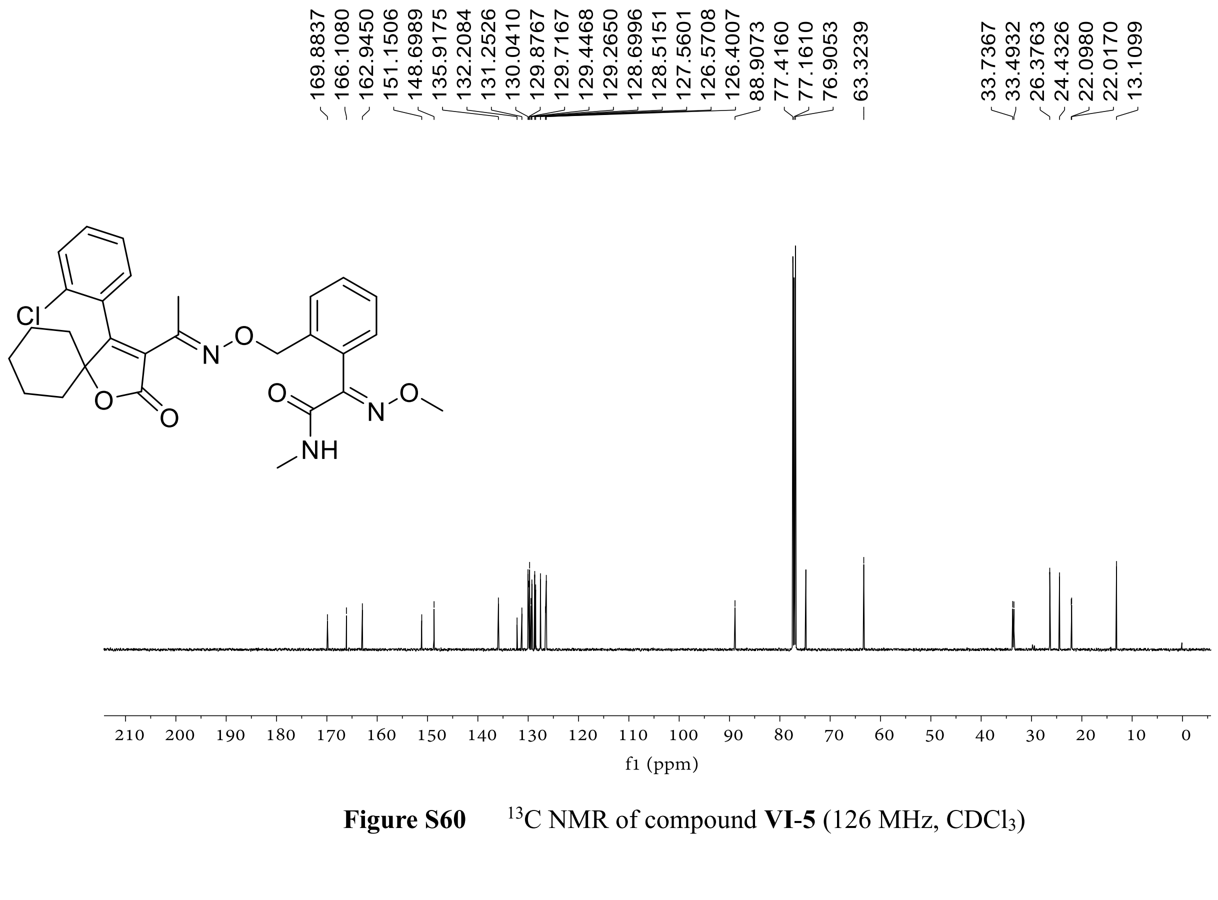

Figure S60 ^{13}C NMR of compound **VI-5** (126 MHz, $CDCl_3$)

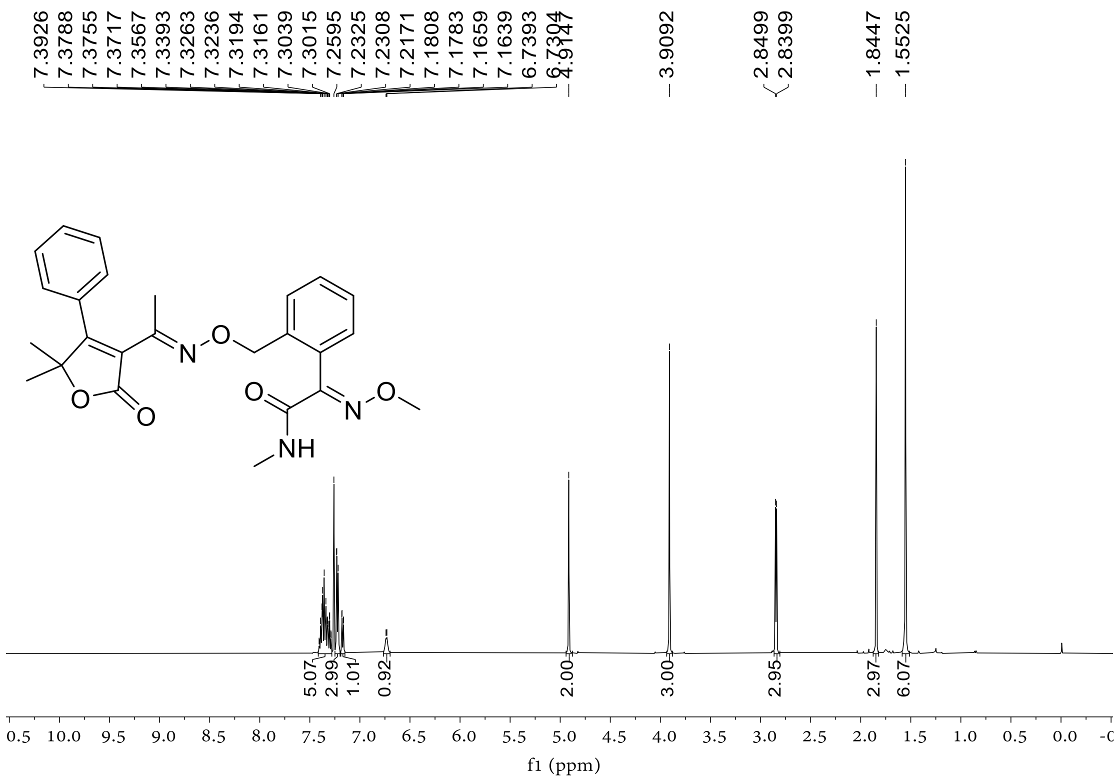

Figure S61 ^{1}H NMR of compound **VI-6** (500 MHz, $CDCl_3$)

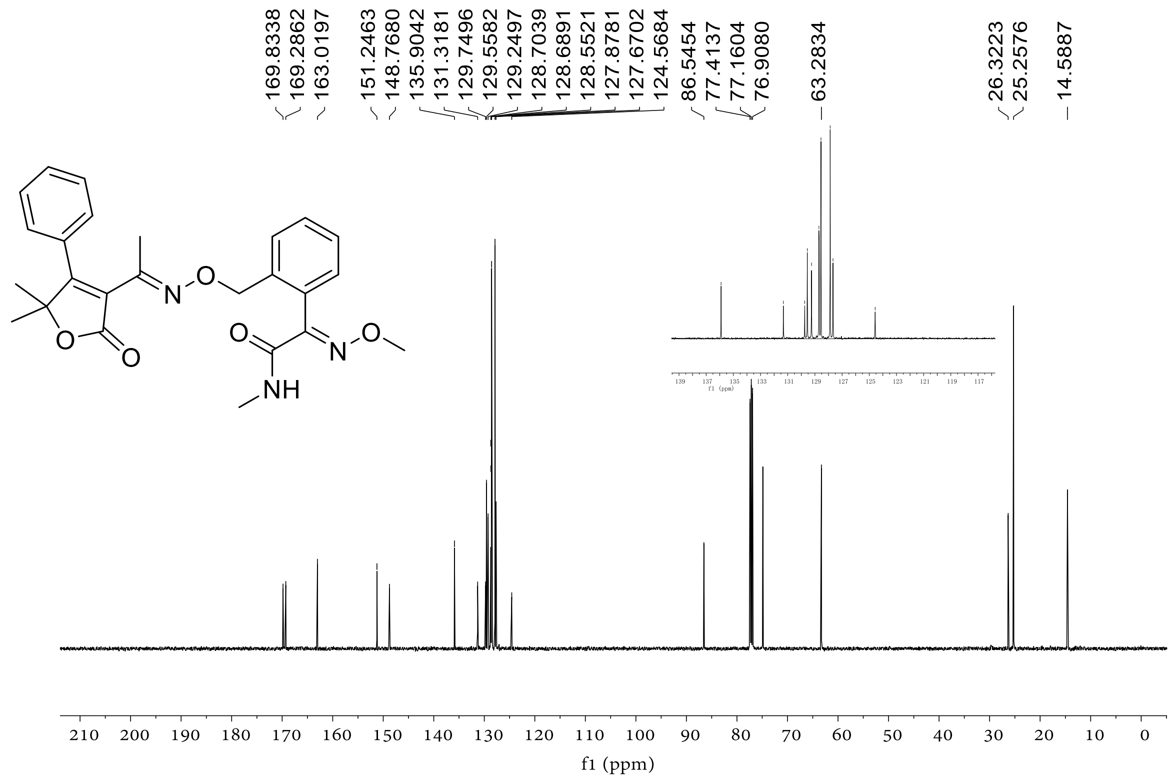

Figure S62 ^{13}C NMR of compound **VI-6** (126 MHz, $CDCl_3$)

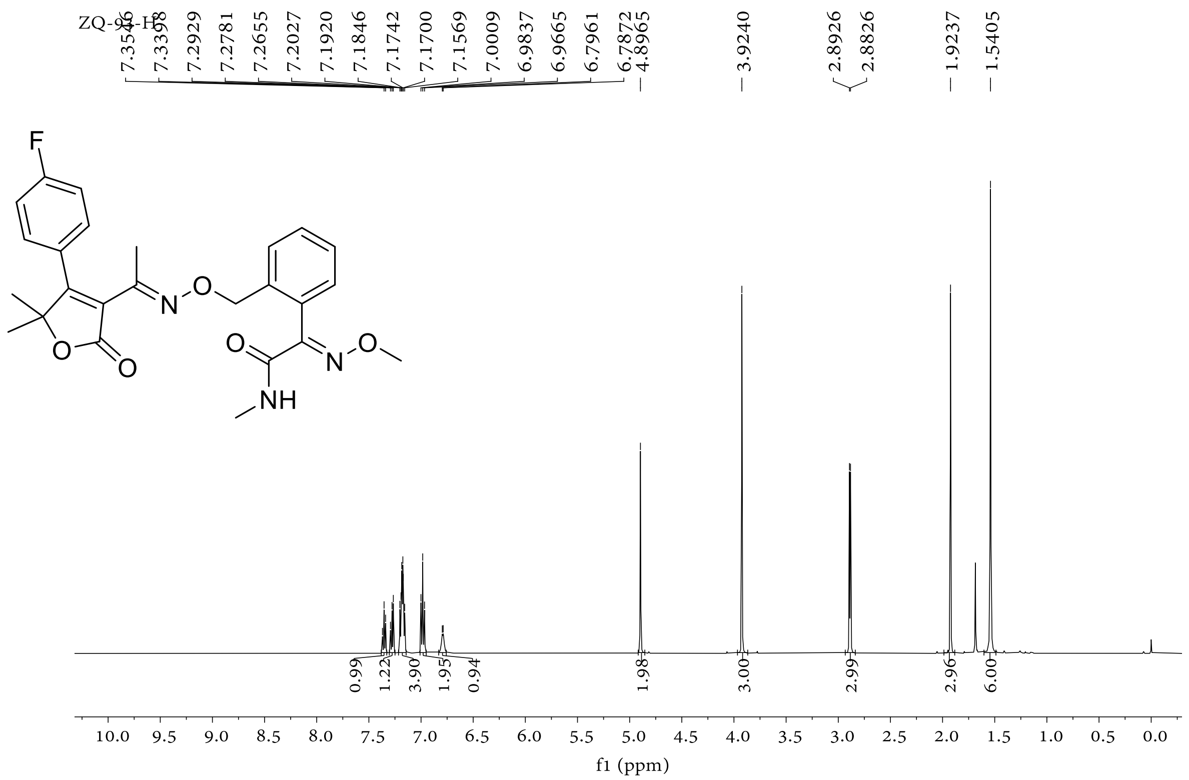

Figure S63 ^{1}H NMR of compound **VI-7** (500 MHz, $CDCl_3$)

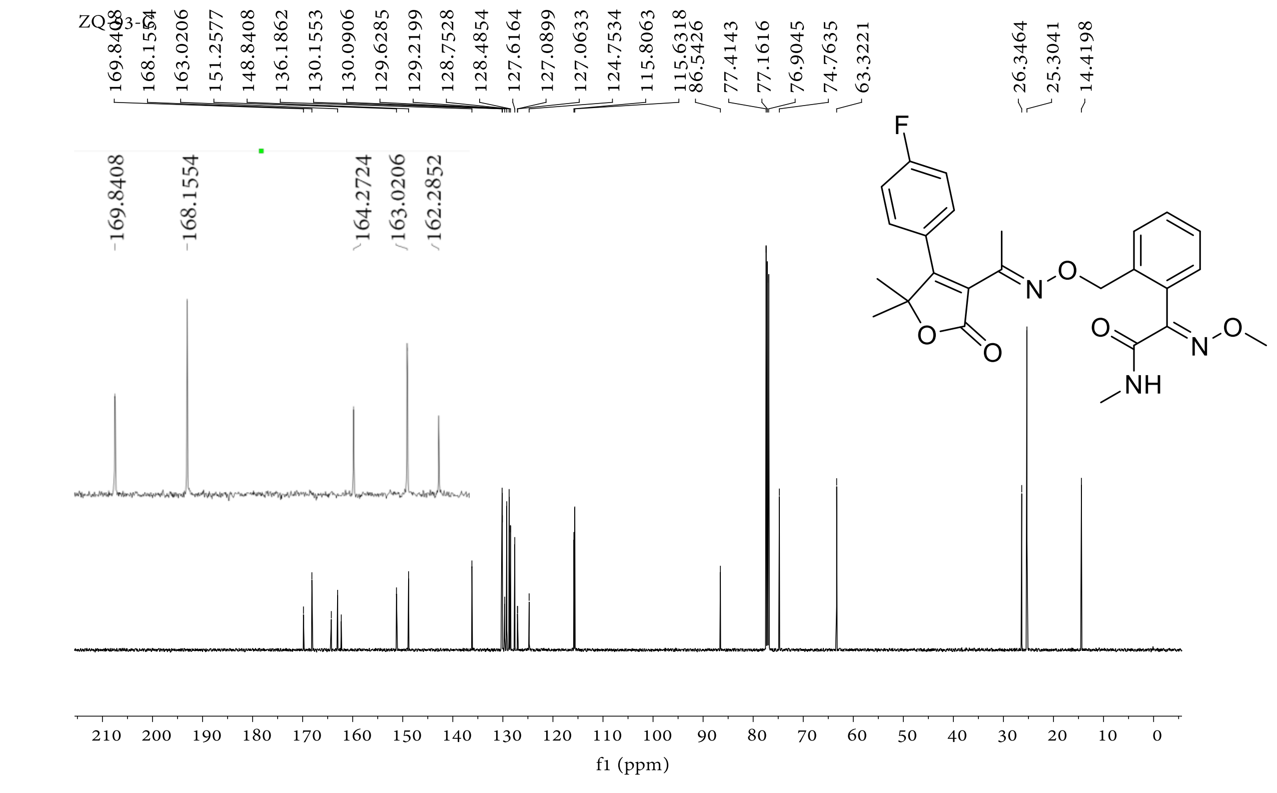

Figure S64 ^{13}C NMR of compound **VI-7** (126 MHz, $CDCl_3$)

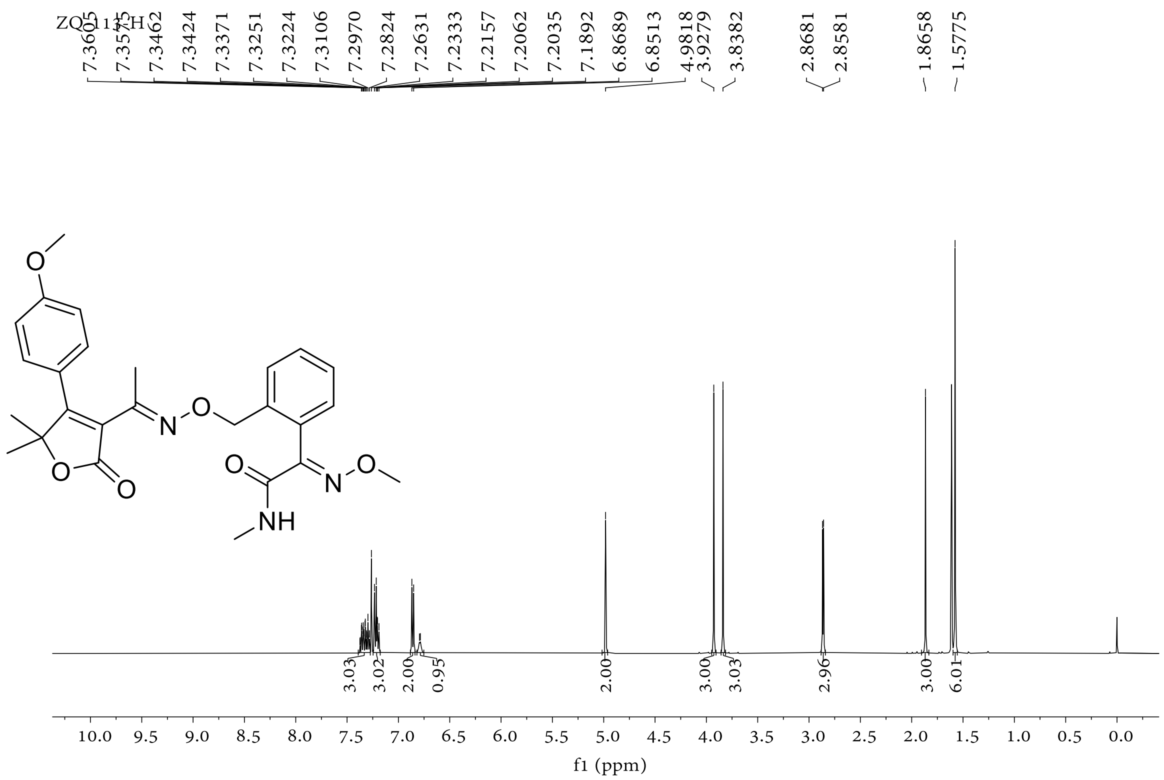

Figure S65 ^{1}H NMR of compound **VI-8** (500 MHz, $CDCl_3$)

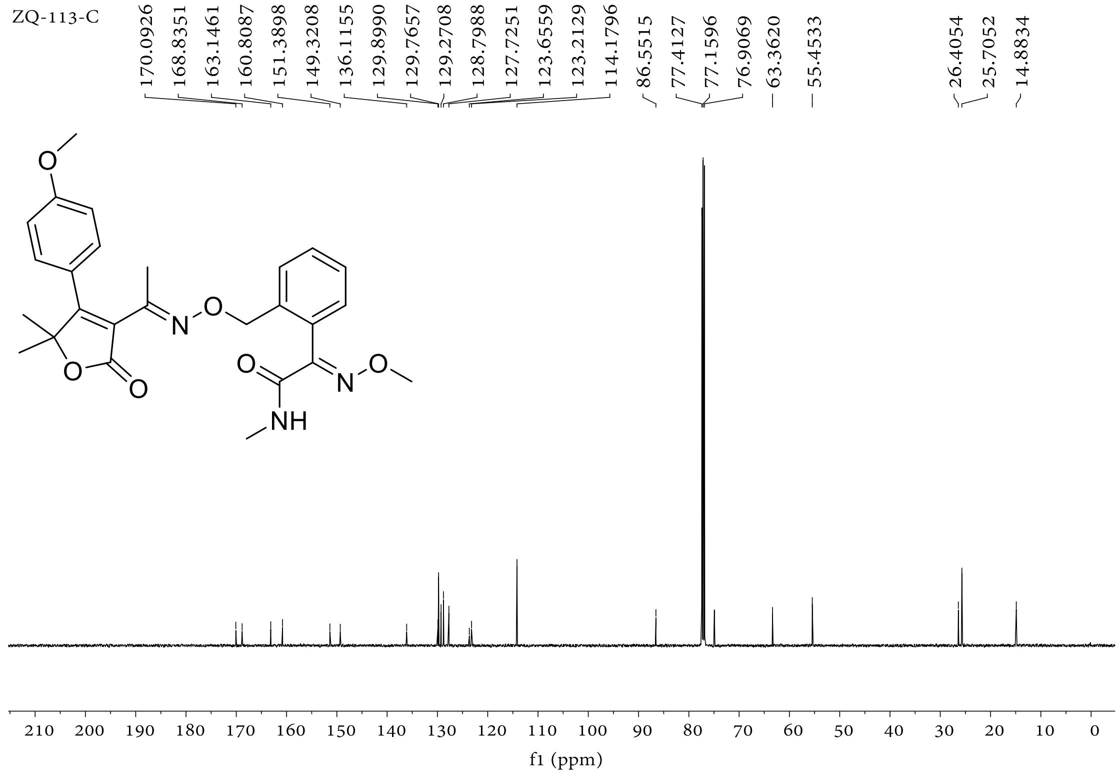

Figure S66 ^{13}C NMR of compound **VI-8** (126 MHz, $CDCl_3$)

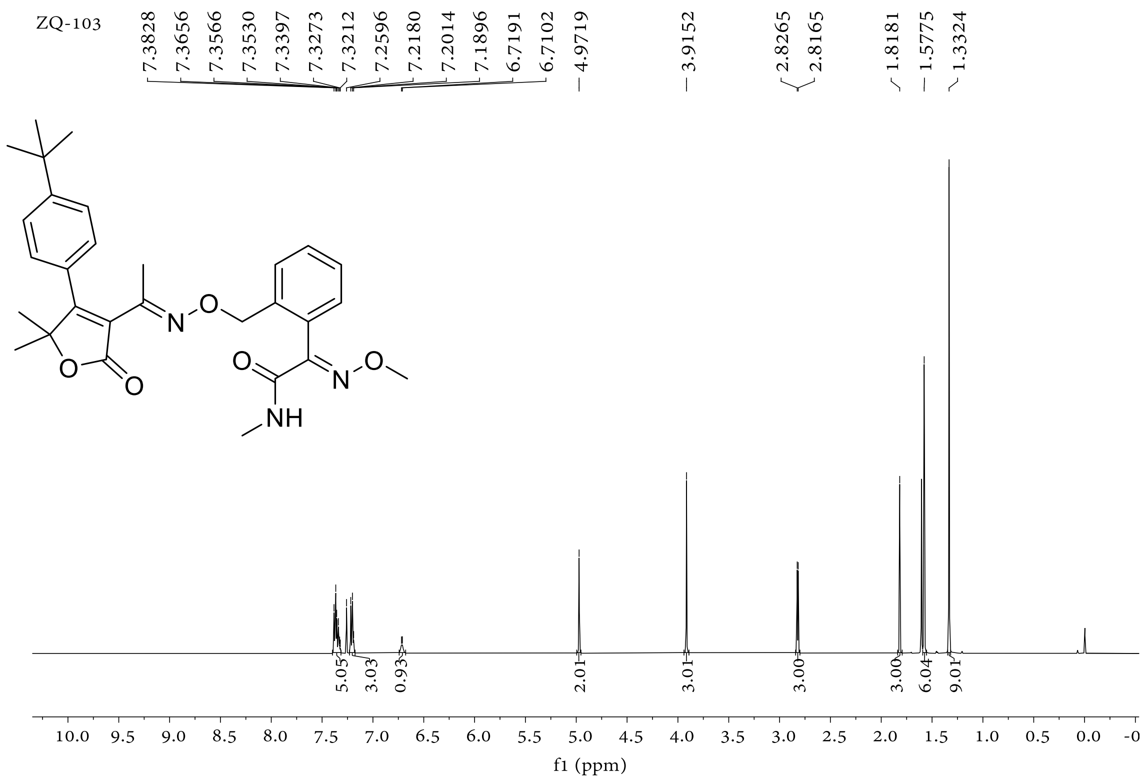

Figure S67 ^{1}H NMR of compound **VI-9** (500 MHz, $CDCl_3$)

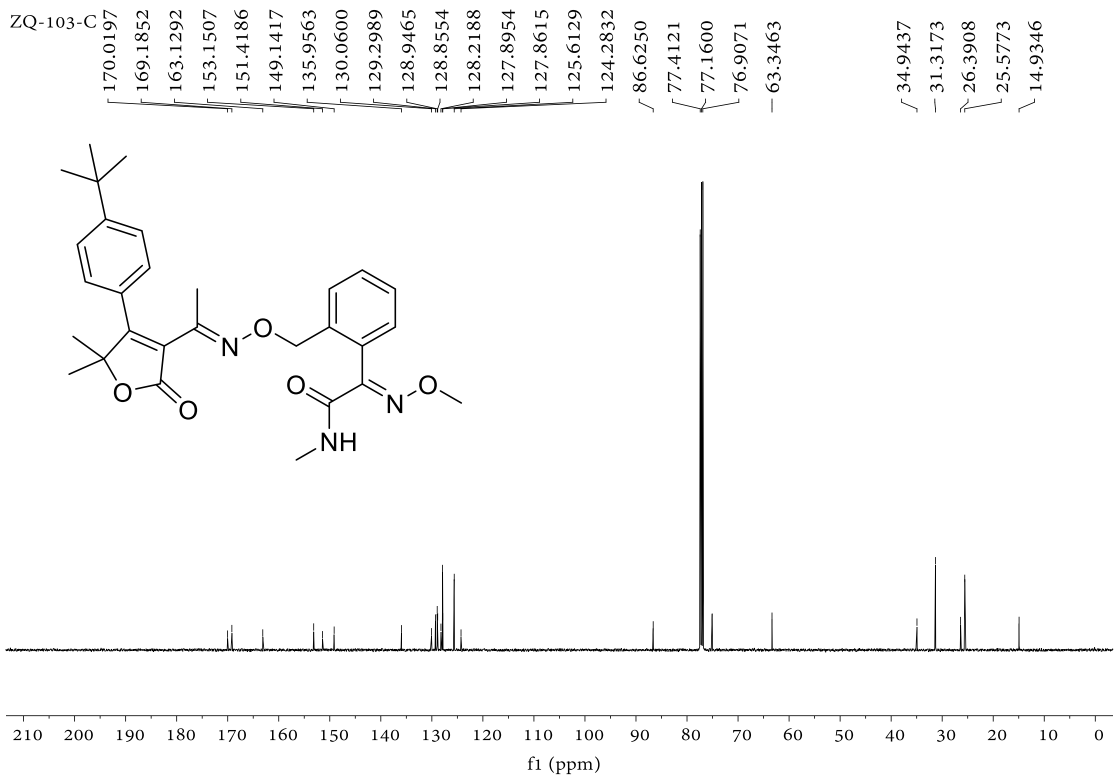

Figure S68 ^{13}C NMR of compound **VI-9** (126 MHz, $CDCl_3$)

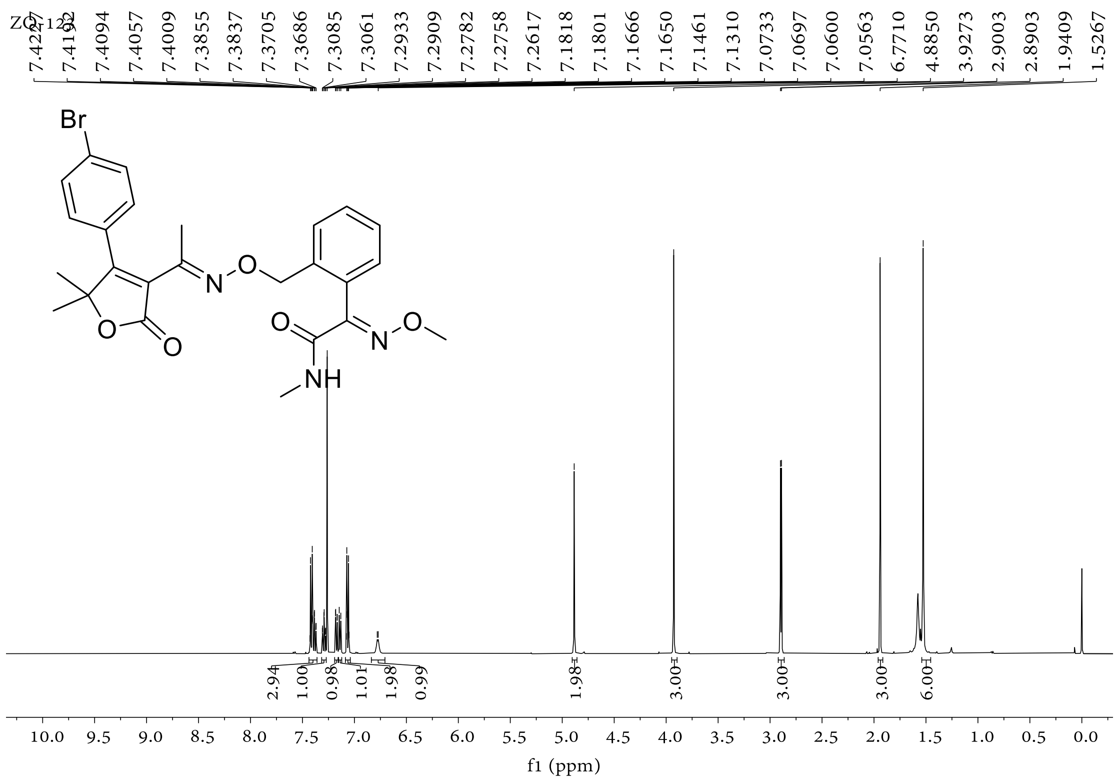

Figure S69 ^{1}H NMR of compound **VI-10** (500 MHz, $CDCl_3$)

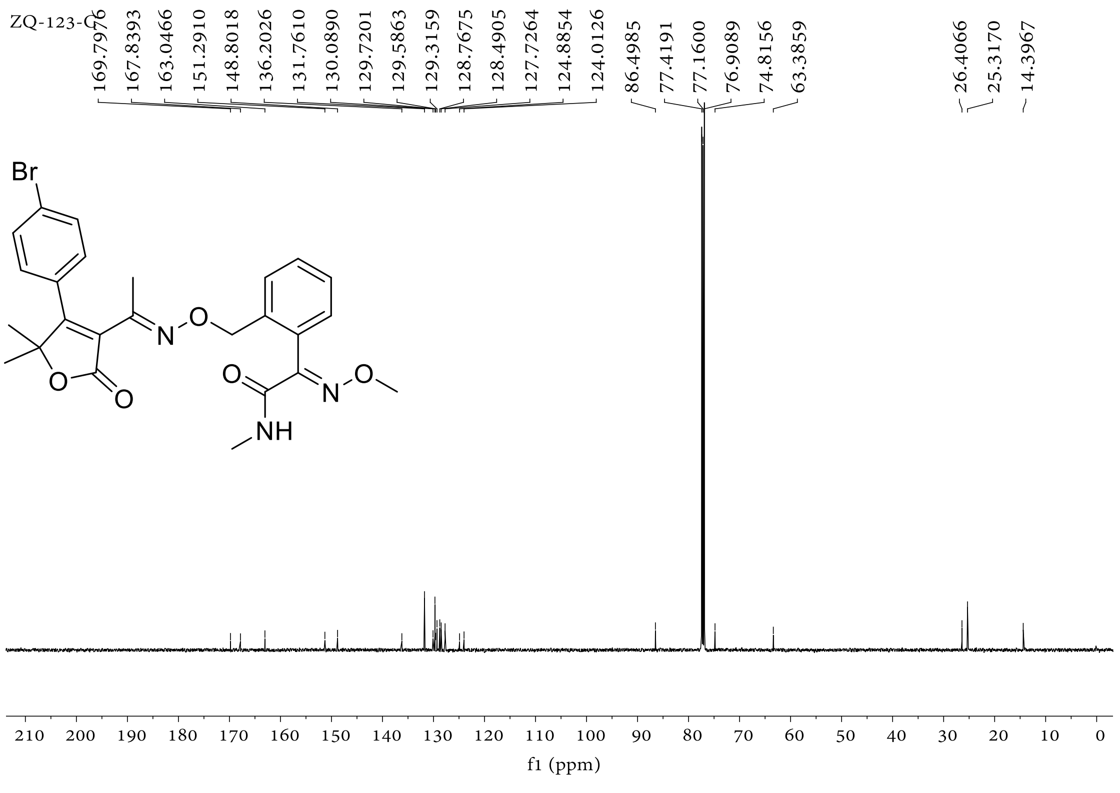

Figure S70 ^{13}C NMR of compound **VI-10** (126 MHz, $CDCl_3$)

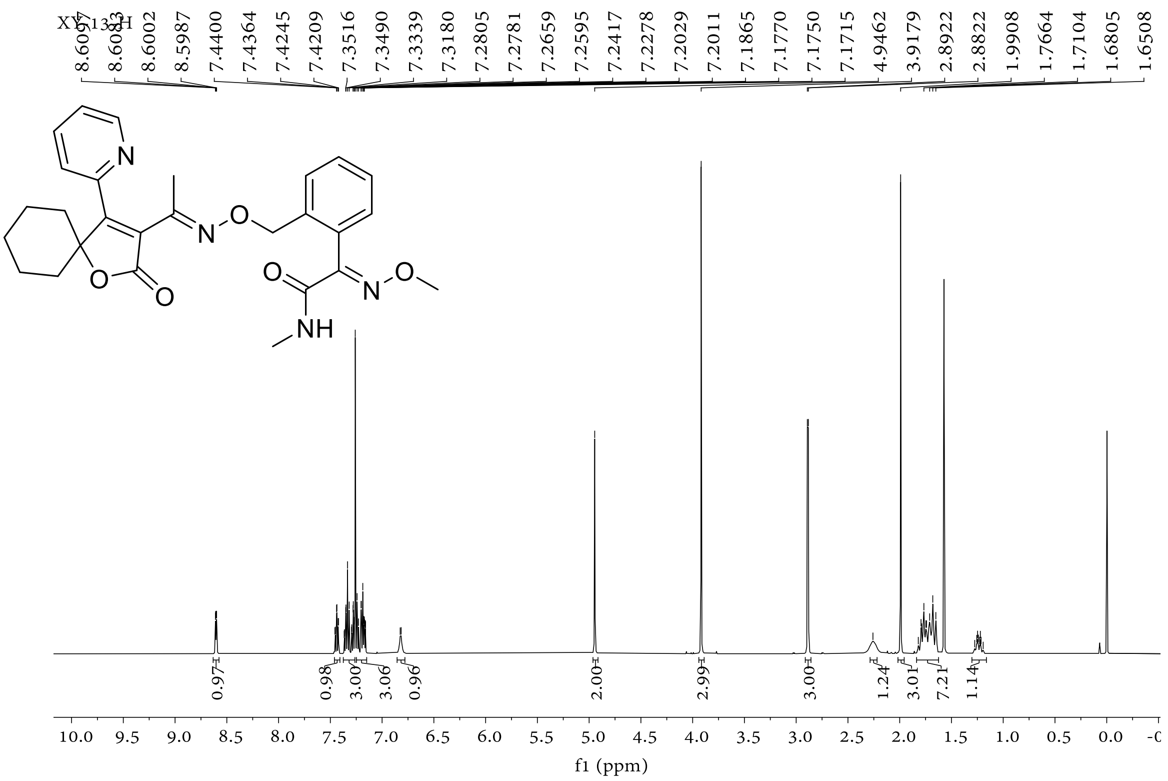

Figure S71 ^{1}H NMR of compound **VI-11** (500 MHz, $CDCl_3$)

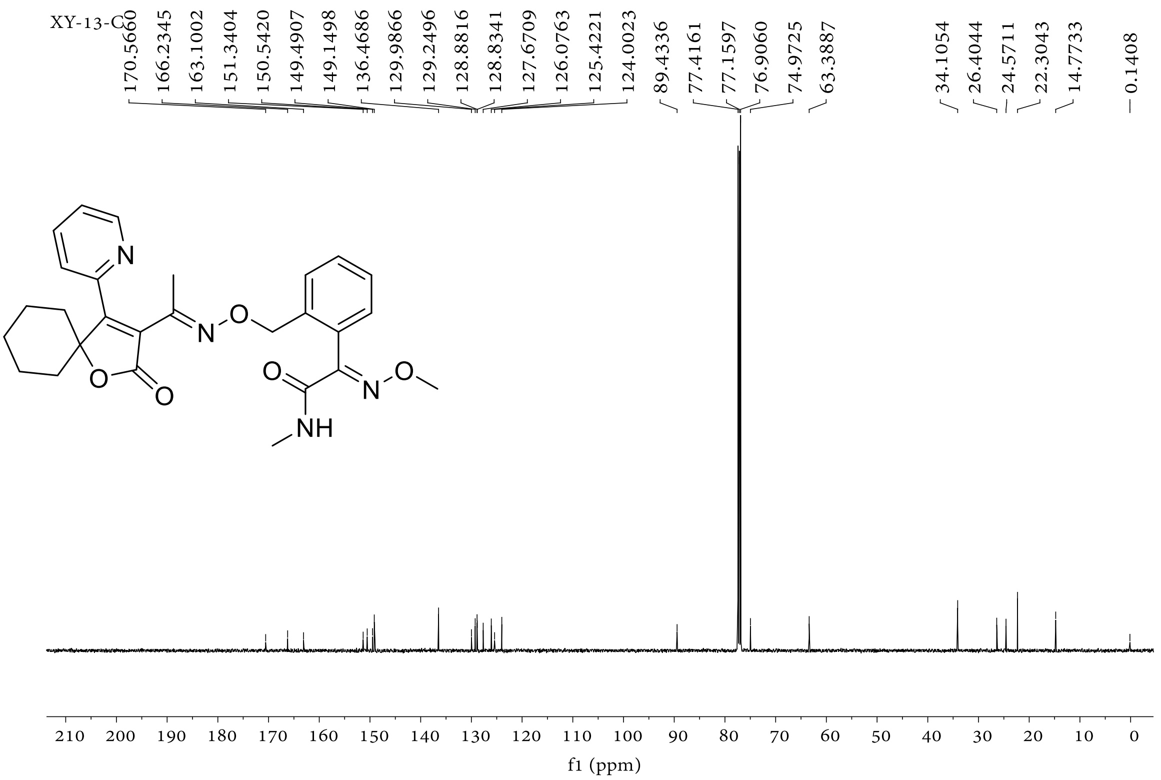

Figure S72 ^{13}C NMR of compound **VI-11** (126 MHz, $CDCl_3$)

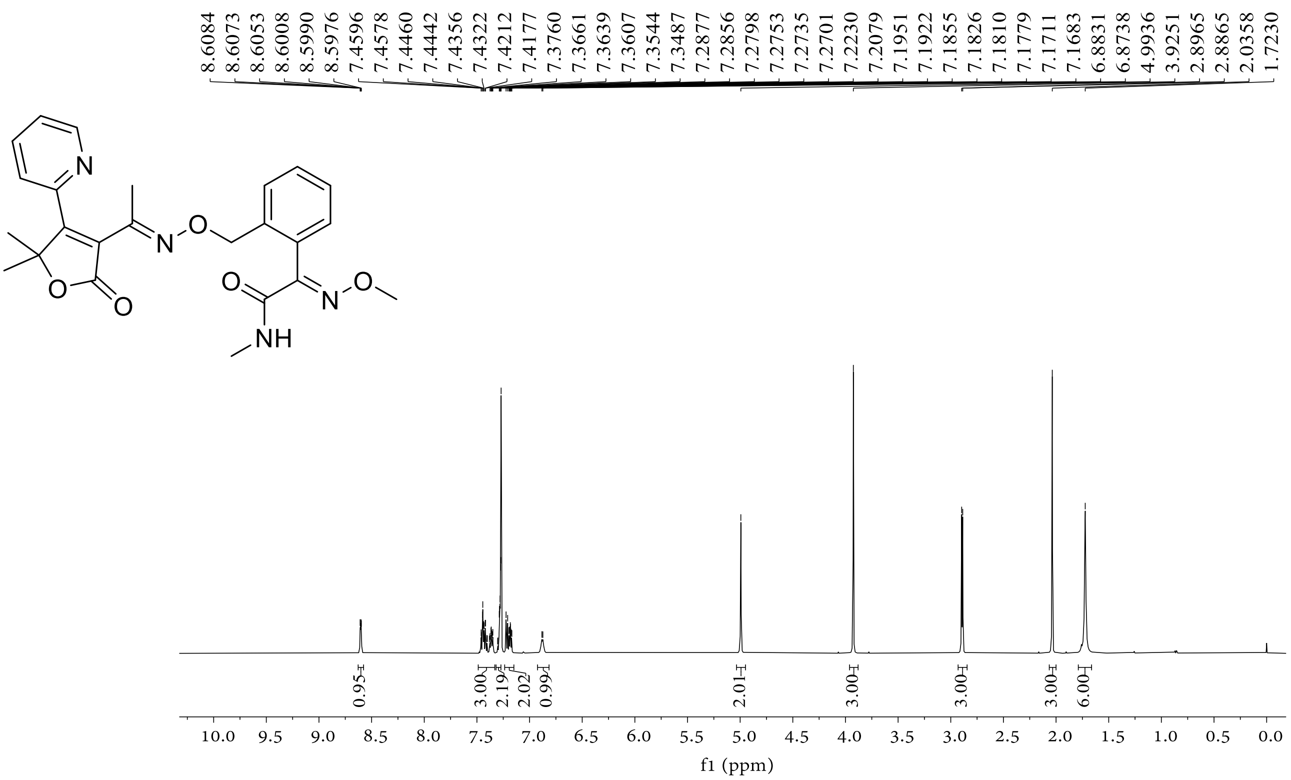

Figure S73 ^{1}H NMR of compound **VI-12** (500 MHz, $CDCl_3$)

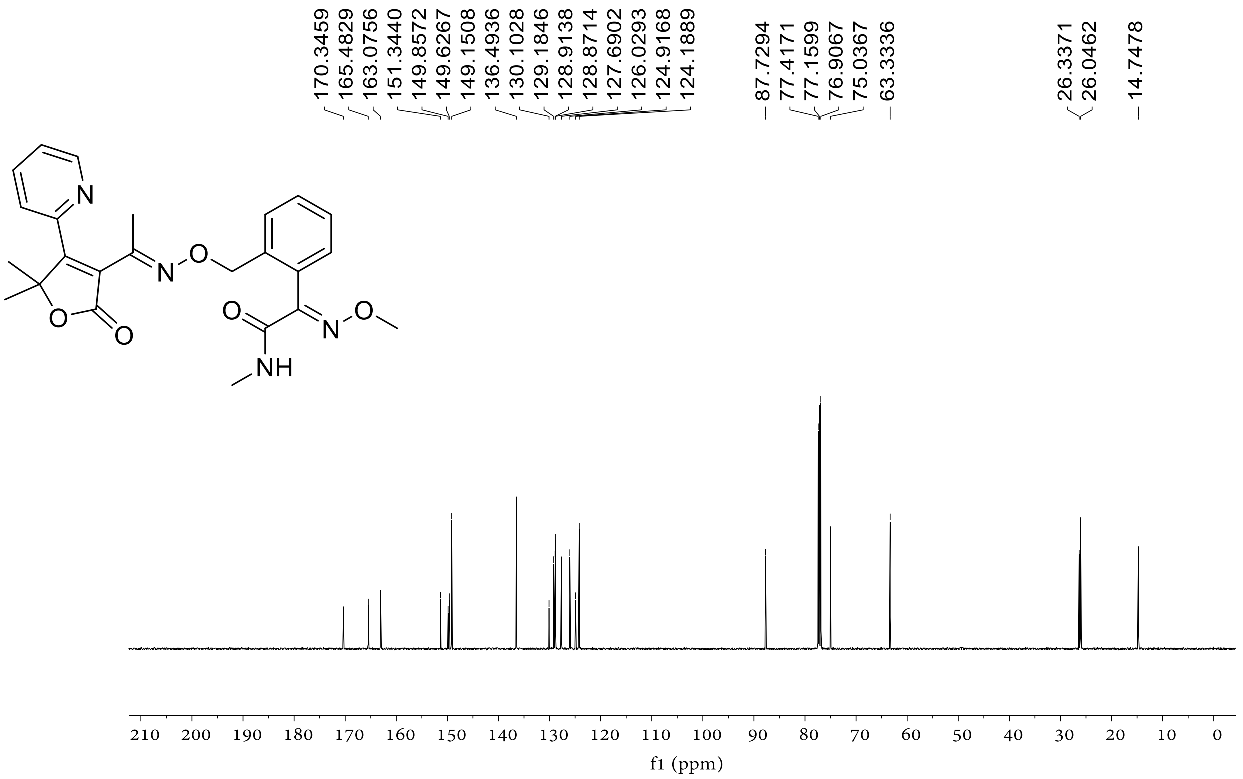

Figure S74 ^{13}C NMR of compound **VI-12** (126 MHz, $CDCl_3$)

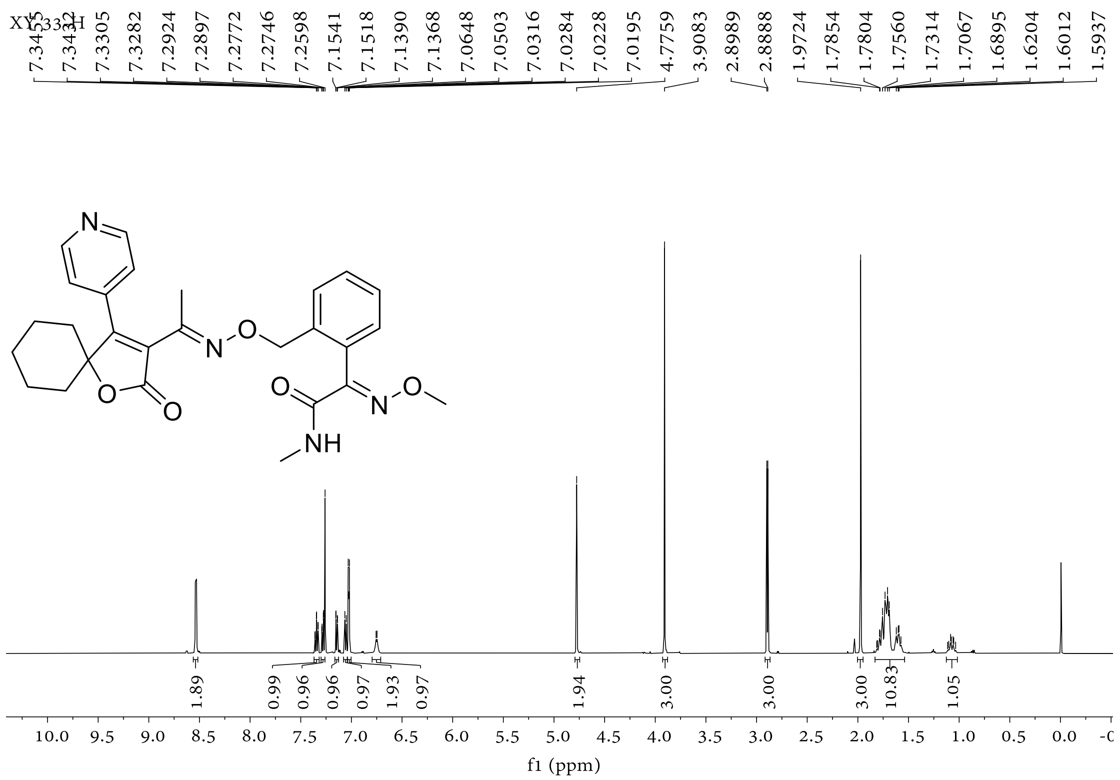

Figure S75 ^{1}H NMR of compound **VI-13** (500 MHz, $CDCl_3$)

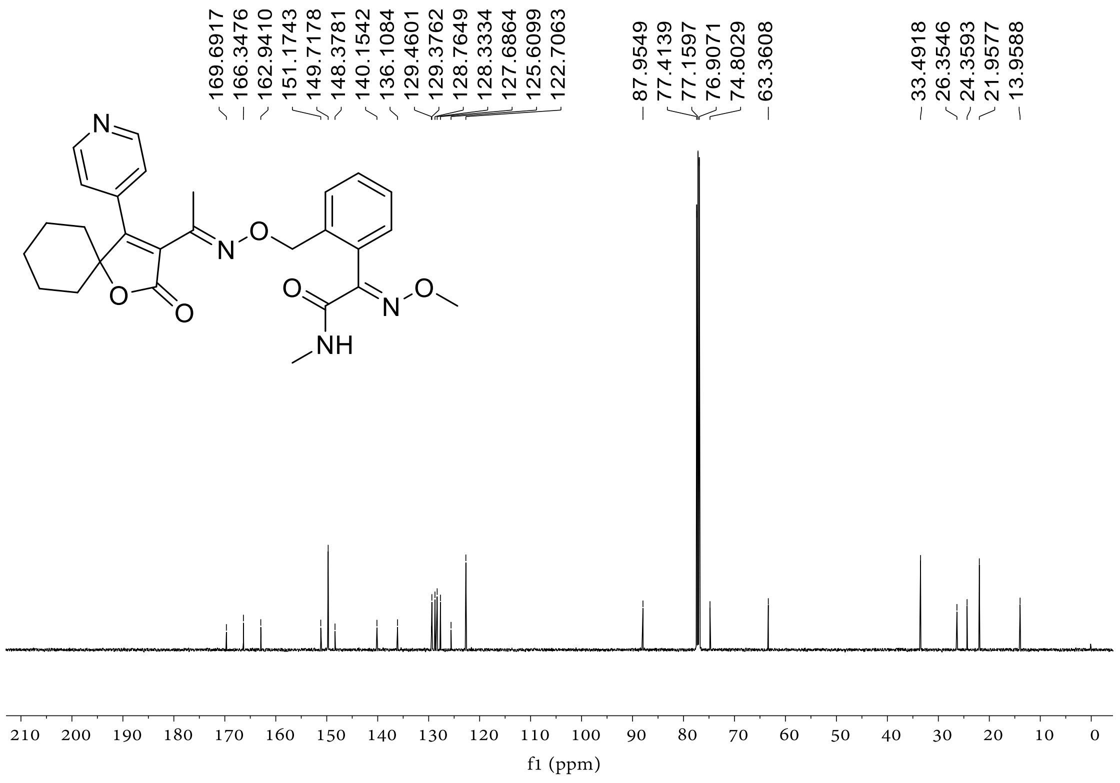

Figure S76 ^{13}C NMR of compound **VI-13** (126 MHz, $CDCl_3$)

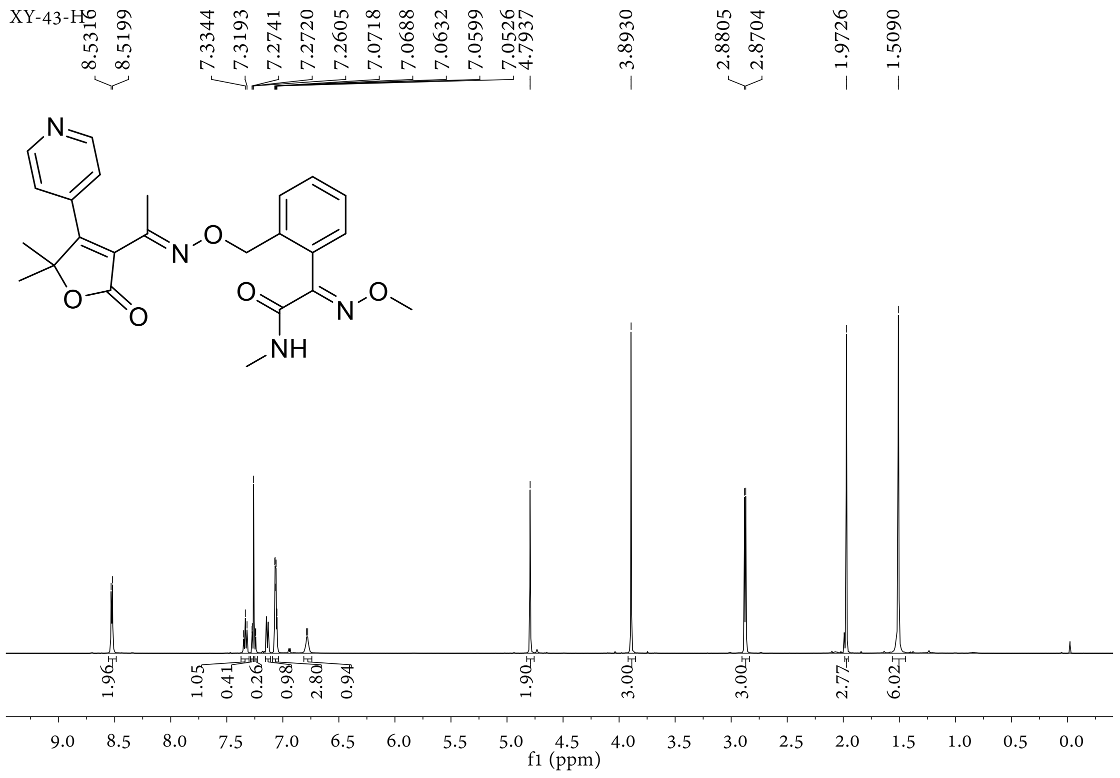

Figure S77 ^{1}H NMR of compound **VI-14** (500 MHz, $CDCl_3$)

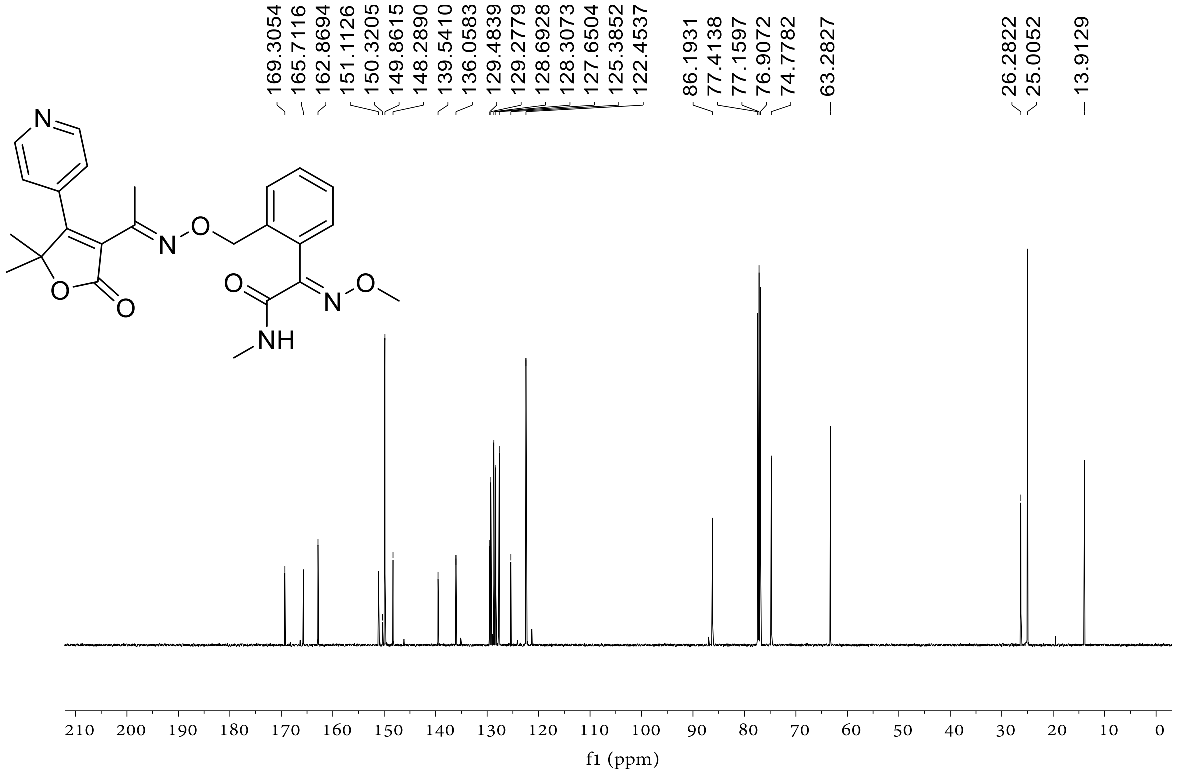

Figure S78 ^{13}C NMR of compound **VI-14** (126 MHz, $CDCl_3$)

2. The high reslution mass spectrometry report spectra of compounds **VI-3**, **VI-8** and **VI-13**

Sample No.	Formula (M)	Ion Formula	Measured m/z	Calc m/z	Diff (ppm)
**VI-3**	$C_{32} H_{39} N_3 O_5$	$C_{32} H_{40} N_3 O_5$	546.2966	546.2962	0.73

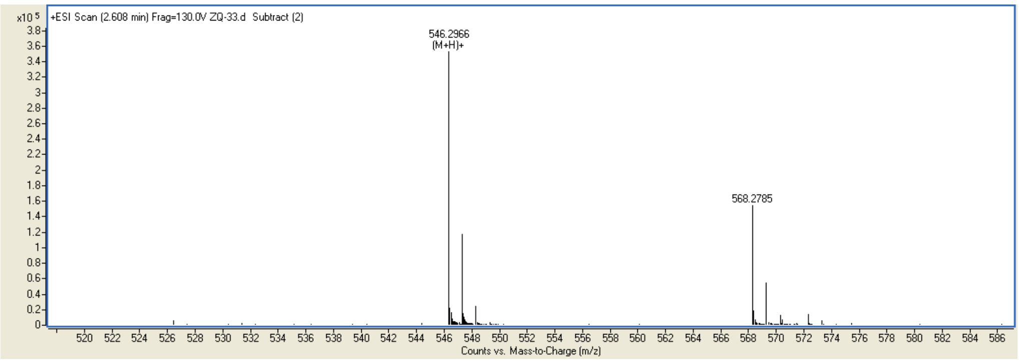

Figure S79 high reslution mass spectrometry report spectra of compound **VI-3**

Sample No.	Formula (M)	Ion Formula	Measured m/z	Calc m/z	Diff (ppm)
**VI-8**	$C_{26} H_{29} N_3 O_6$	$C_{26} H_{30} N_3 O_6$	480.2131	480.2129	0.42

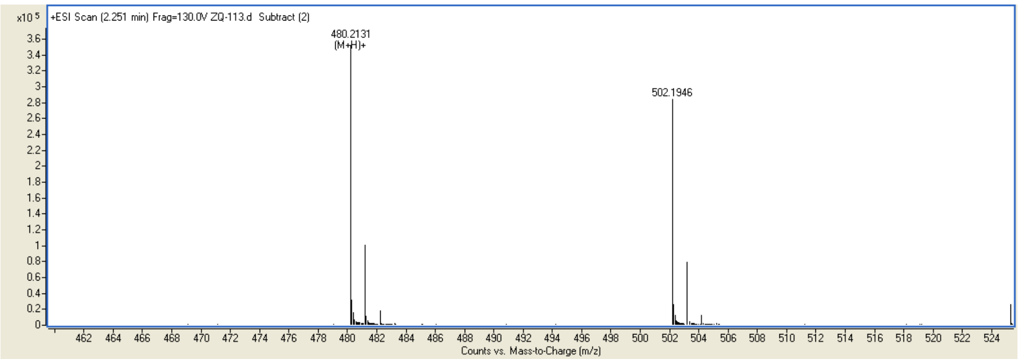

Figure S80 high reslution mass spectrometry report spectra of compound **VI-8**

Sample No.	Formula (M)	Ion Formula	Measured m/z	Calc m/z	Diff (ppm)
**VI-13**	$C_{27} H_{30} N_4 O_5$	$C_{27} H_{31} N_4 O_5$	491.2291	491.2289	0.41

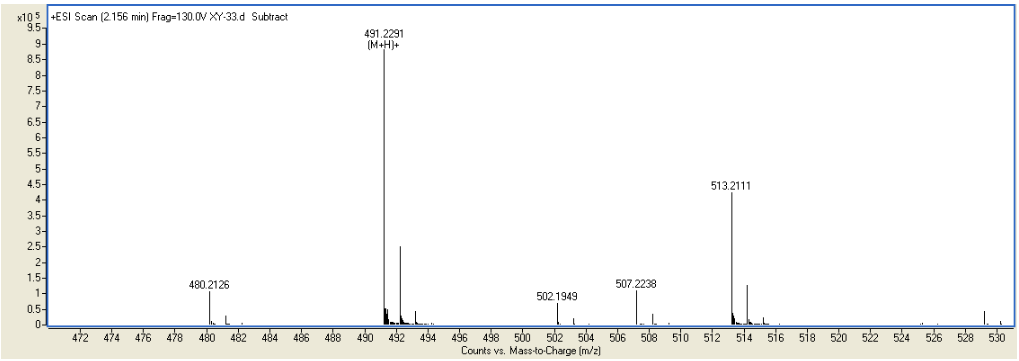

Figure S81 high reslution mass spectrometry report spectra of compound **VI-13**

3. The X-ray crystal structure of target compound **IV-6**

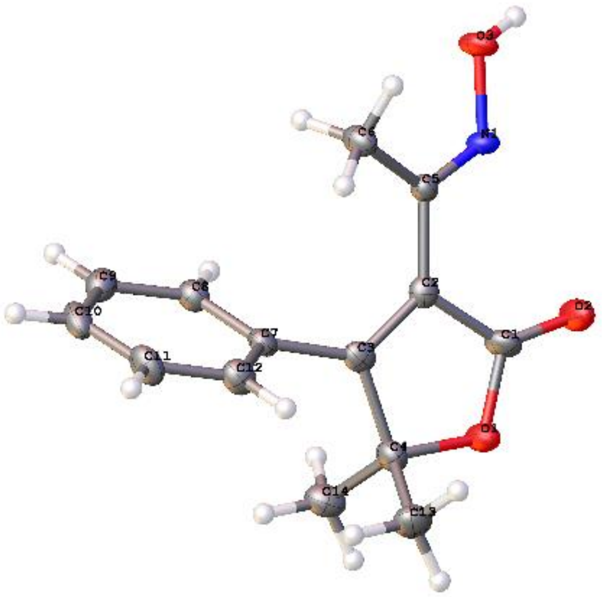

Figure S82 The X-ray crystal structure of target compound **IV-6**

Table S1. Crystal data and structure refinement of compound 7471 (IV-6, 2162653)

Empirical formula	$C_{14}H_{15}NO_3$
Formula weight	245.27
Temperature / K	125.50(14)
Crystal system	tetragonal
Space group	$P4_12_12$
a / Å, b / Å, c / Å	8.8563(5), 8.8563(5), 32.1060(18)
α/°, β/°, γ/°	90.00, 90.00, 90.00
Volume / $Å^3$	2518.2(2)
Z	8
ρ_{calc} / mg mm^{-3}	1.294
μ / mm^{-1}	0.091
F(000)	1040
Crystal size / mm^3	0.55 × 0.34 × 0.02
2Θ range for data collection	6.5 to 51.96°
Index ranges	-10 ≤ h ≤ 10, -10 ≤ k ≤ 10, -39 ≤ l ≤ 25
Reflections collected	7531
Independent reflections	2427[R(int) = 0.0522 (inf-0.9Å)]
Data/restraints/parameters	2427/0/167
Goodness-of-fit on F^2	1.085
Final R indexes [I>2σ (I) i.e. F_o>4σ (F_o)]	R_1 = 0.0514, wR_2 = 0.1026
Final R indexes [all data]	R_1 = 0.0655, wR_2 = 0.1114
Largest diff. peak/hole / e $Å^{-3}$	0.204/-0.181

Completeness	0.9944

TableS2 Fractional Atomic Coordinates (×104) and Equivalent Isotropic Displacement Parameters (Å2×103) for exp_7471. Ueq is defined as 1/3 of of the trace of the 43rthogonalized UIJ tensor.

Atom	*x*	*y*	*z*	U(eq)
O3	9425(2)	6758.6(19)	22.1(5)	25.9(4)
N1	8955(2)	7582(2)	372.3(6)	20.7(5)
O1	7667(2)	9457(2)	1504.8(5)	27.7(5)
O2	6858(2)	9226(2)	852.0(5)	33.9(5)
C6	10567(3)	5720(3)	725.1(8)	25.0(6)
C5	9535(3)	7058(3)	711.0(7)	17.3(5)
C10	13914(3)	5898(3)	1887.8(8)	27.6(6)
C14	9822(3)	10179(3)	1918.3(8)	30.3(7)
C3	9736(3)	7833(3)	1475.1(7)	20.3(6)
C2	9106(3)	7859(3)	1095.0(7)	19.2(6)
C1	7772(3)	8877(3)	1115.0(7)	24.9(6)
C4	8868(3)	8860(3)	1769.9(7)	23.7(6)
C11	12569(3)	5394(3)	2054.9(8)	28.4(6)
C12	11199(3)	5991(3)	1923.5(8)	25.3(6)
C8	12532(3)	7597(3)	1442.3(7)	21.8(5)
C7	11173(3)	7118(3)	1613.9(7)	19.8(6)
C9	13900(3)	6997(3)	1579.1(8)	25.5(6)
C13	8099(3)	8041(3)	2129.1(8)	29.6(6)

Table S3 Anisotropic Displacement Parameters (Å2×103) for exp_7471. The Anisotropic displacement factor exponent takes the form: -2π2[h2a*2U11+…+2hka×b×U12]

Atom	U_{11}	U_{22}	U_{33}	U_{23}	U_{13}	U_{12}
O3	35.9(11	30.4(10)	11.4(9)	-2.2(8)	-0.1(9)	10.4(8)
N1	23.9(11)	24.0(12)	14.4(10)	-4.3(9)	1.9(9)	4.2(10)
O1	29(1)	36.3(10)	18.0(9)	-3.9(8)	-2.0(8)	11.7(9)
O2	31.9(11)	49.6(13)	20.1(10)	-1.7(9)	-5.0(9)	19.1(10)
C6	30.3(15)	26.2(15)	18.6(14)	-1.2(11)	-4.5(11)	5.3(13)
C5	17.0(12)	19.9(13)	15.2(12)	0.9(10)	-0.8(10)	-0.1(11)
C10	25.6(15)	24.8(15)	32.4(15)	-7.9(12)	-12.0(13)	4.1(12)
C14	38.1(17)	28.7(16)	24.0(14)	-2.8(12)	0.0(13)	-3.3(13)
C3	22.1(13)	20.5(14)	18.3(13)	3.1(10)	1.5(11)	-5.6(12)
C2	19.1(13)	17.1(13)	21.4(14)	3.4(10)	-0.3(11)	-1.5(11)
C1	31.2(16)	30.2(15)	13.4(13)	0.3(11)	0.9(11)	3.0(14)
C4	24.6(14)	27.5(15)	18.9(13)	-0.4(11)	-4.0(11)	1.3(13)
C11	33.0(16)	27.0(14)	25.2(14)	5.8(12)	-10.5(12)	0.0(14)

C12	26.5(15)	25.9(15)	23.4(14)	3.1(11)	-1.2(12)	-5.2(13)
C8	23.7(13)	21.4(13)	20.2(13)	0.4(11)	-2.1(11)	-0.3(12)
C7	21.7(13)	22.5(14)	15.2(13)	-2.4(10)	-3.1(11)	-1.2(11)
C9	21.0(14)	26.3(15)	29.1(15)	-5.5(12)	-0.2(12)	-0.4(12)
C13	30.0(16)	36.7(17)	21.9(14)	2.2(13)	5.2(13)	-1.4(13)

Table S4 Bond Lengths for exp_7471.

Atom	Atom	Length/Å
O3	N1	1.403(2)
N1	C5	1.289(3)
O1	C1	1.356(3)
O1	C4	1.462(3)
O2	C1	1.210(3)
C6	C5	1.497(3)
C5	C2	1.472(3)
C10	C11	1.381(4)
C10	C9	1.389(4)
C14	C4	1.519(4)
C3	C2	1.342(3)
C3	C4	1.521(4)
C3	C7	1.489(3)
C2	C1	1.487(4)
C4	C13	1.523(3)
C11	C12	1.389(4)
C12	C7	1.409(3)
C8	C7	1.390(4)
C8	C9	1.394(4)

Table S5 Bond Angles for exp_7471.

Atom	Atom	Atom	Angle/˚
C5	N1	O3	111.75(19)
C1	O1	C4	110.48(19)
N1	C5	C6	123.6(2)
N1	C5	C2	115.5(2)
C2	C5	C6	120.9(2)
C11	C10	C9	119.7(2)
C2	C3	C4	110.2(2)
C2	C3	C7	129.4(2)
C7	C3	C4	120.0(2)
C5	C2	C1	122.2(2)
C3	C2	C5	130.2(2)
C3	C2	C1	107.5(2)

O1	C10	C2	108.9(2)
O2	C1	C1	120.1(2)
O2	C1	C2	131.0(2)
O1	C4	C14	108.0(2)
O1	C4	C3	102.82(18)
O1	C4	C13	106.7(2)
C14	C4	C3	112.0(2)
C14	C4	C13	112.2(2)
C3	C4	C13	114.4(2)
C10	C11	C12	120.8(2)
C11	C12	C7	119.9(2)
C7	C8	C9	120.7(2)
C12	C7	C3	121.7(2)
C8	C7	C3	119.4(2)
C8	C7	C12	118.8(2)
C10	C9	C8	120.0(2)

Table S6 Torsion Angles for exp_7471.

A	B	C	D	**Angle/°**
O3	N1	C5	C6	-0.7(3)
O3	N1	C5	C2	179.8(2)
N1	C5	C2	C3	-163.6(3)
N1	C5	C2	C1	17.7(3)
C6	C5	C2	C3	16.9(4)
C6	C5	C2	C1	-161.8(2)
C5	C2	C1	O1	-179.6(2)
C5	C2	C1	O2	0.0(4)
C10	C11	C12	C7	0.4(4)
C3	C2	C1	O1	1.5(3)
C3	C2	C1	O2	-178.9(3)
C2	C3	C4	O1	0.2(3)
C2	C3	C4	C14	-115.4(2)
C2	C3	C4	C13	115.5(2)
C2	C3	C7	C12	-119.8(3)
C2	C3	C7	C8	61.9(4)
C1	O1	C4	C14	119.2(2)
C1	O1	C4	C3	0.7(3)
C1	O1	C4	C13	-120.0(2)
C4	O1	C1	O2	179.0(2)
C4	O1	C1	C2	-1.3(3)
C4	C3	C2	C5	-179.8(2)
C4	C3	C2	C1	-1.0(3)
C4	C3	C7	C12	68.5(3)

C4	C3	C7	C8	-109.8(3)
C11	C10	C9	C8	0.7(4)
C11	C12	C7	C3	-177.6(2)
C11	C12	C7	C8	0.7(4)
C7	C3	C2	C5	7.8(4)
C7	C3	C2	C1	-173.3(2)
C7	C3	C4	O1	173.4(2)
C7	C3	C4	C14	57.7(3)
C7	C3	C4	C13	-71.3(3)
C7	C8	C9	C10	0.5(4)
C9	C10	C11	C12	-1.1(4)
C9	C8	C7	C3	177.2(2)
C9	C8	C7	C12	-1.2(4)

Table S7 Hydrogen Atom Coordinates (Å×104) and Isotropic Displacement Parameters (Å2×103) for exp_7471.

Atom	*x*	*y*	*z*	U(eq)
H3	9073	7159	-194	39
H6A	10262	4989	512	38
H6B	10510	5248	1001	38
H6C	11606	6049	671	38
H10	14846	5494	1984	33
H14A	10263	10696	1667	45
H14B	10632	9807	2099	45
H14C	9187	10888	2074	45
H11	12580	4629	2262	34
H12	10281	5640	2042	30
H8	12529	8343	1229	26
H9	14823	7339	1461	31
H13A	7474	8757	2286	44
H13B	8867	7610	2314	44
H13C	7461	7230	2019	44

4. The cell variance of *S. sclerotiorum* treated with **VI-3** and **TRI** observed by TEM

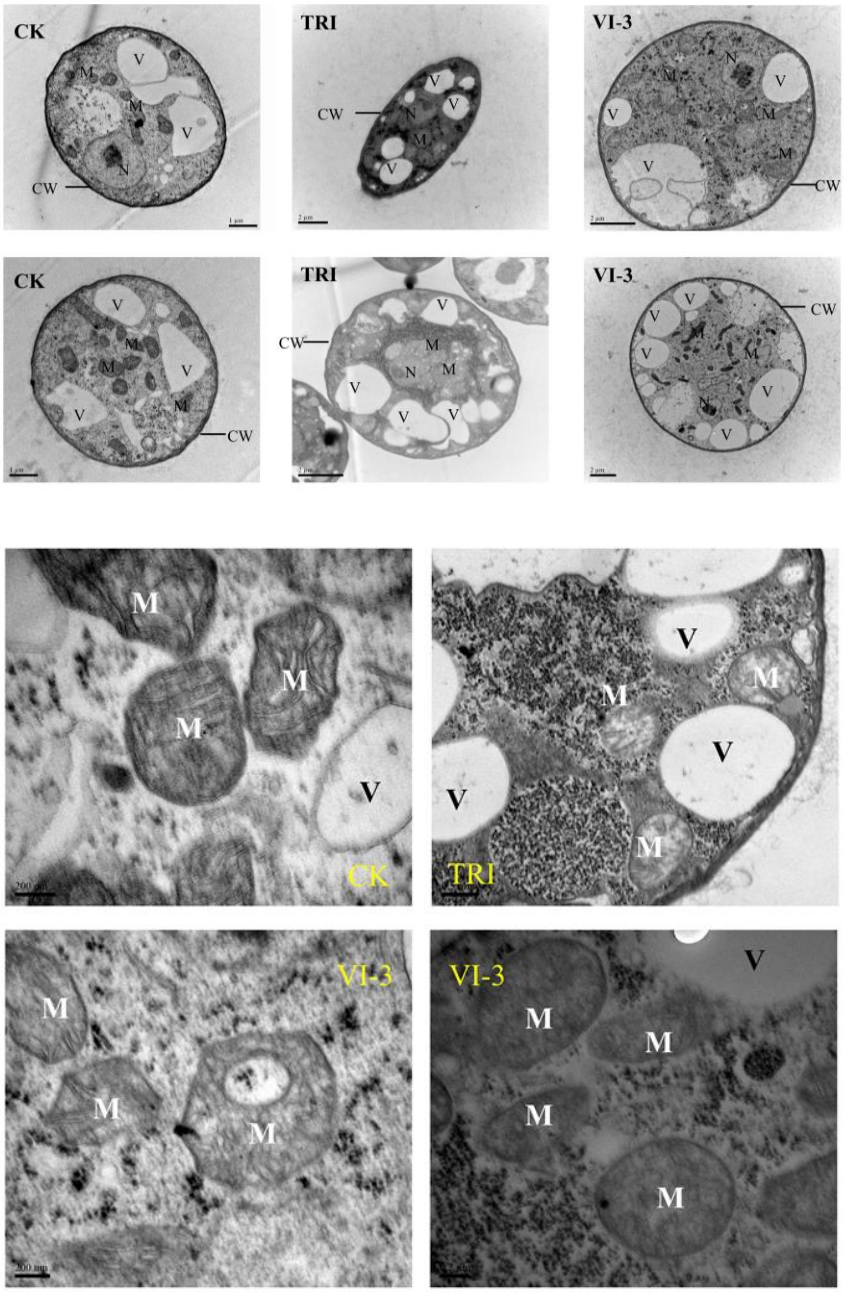

Figure S83 The cell variance of *S. sclerotiorum* treated with **VI-3** and TRI observed by TEM

Note: CW: cell wall; N: nucleus; M: mitochondrion; V: vacuole

5. The mycelium growth inhibition of *S. sclerotiorum* treated with **VI-3** and **TRI** by SEM

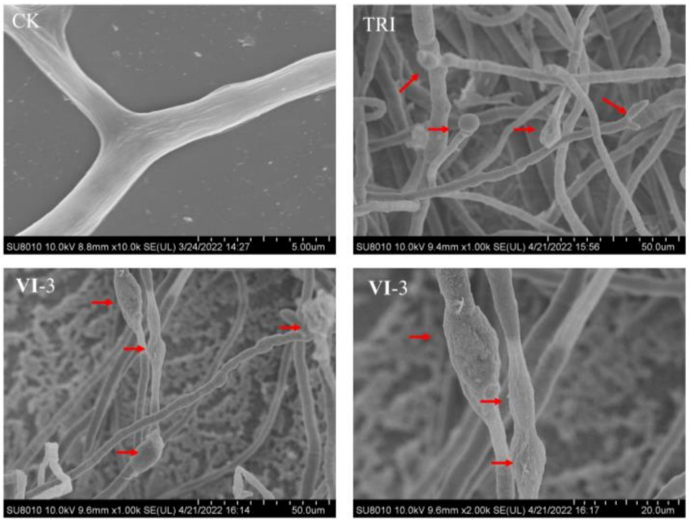

Figure S84 The mycelium growth inhibition of *S. sclerotiorum* treated with **VI-3** and **TRI** by SEM

6. Interaction of compounds **V-3**, **V-6**, **VI-7** and **TRI** with target by molecular docking

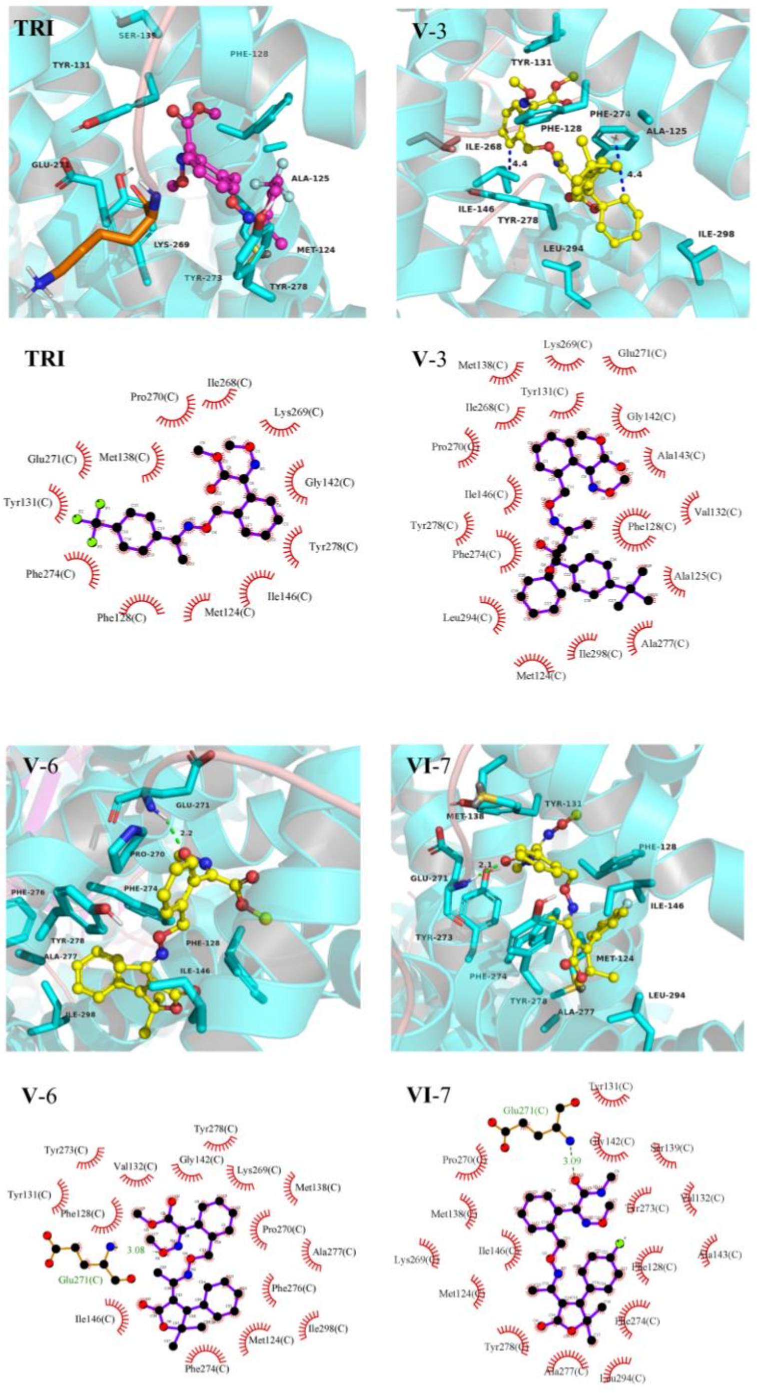

Figure S85 Interaction of compounds **V-3**, **V-6**, **VI-7** and TRI with target by molecular docking

References

[1] Zhang, Q., Li, Y., Xu, L., Ma, H., Li, X., Wang, M., Synthesis and Fungicidal Activity of Novel Butenolide Compounds Containing Oxime Ether Moiety. *Chin. J. Org. Chem.*, **2022**, 42, 2438-2448.
